# Supplementary material for: Understanding challenges as they impact on hospital-level care for pre-eclampsia in rural Ethiopia: a qualitative study
Source: BMJ Open. 2023 Apr 17;13(4):e061500. doi: 10.1136/bmjopen-2022-061500 (PMC10111927; doi:10.1136/bmjopen-2022-061500)
Supplement: Supplementary data [file bmjopen-2022-061500supp001.pdf]

Codebook

Nodes\\Synthesis 1_TR_TE_combined\\Synthesis 2_themes_V1.0_2\\Synthesis 3

Name	Description	Files	References
1.0 Inconsistencies and inequity_Analysis 1	Inconsistency in how the 'rules' are applied may exacerbate inequities but may also endeavour to ameliorate them	0	0
Clinical factors		0	0
Clinical competence	examples of factors affecting ability to provide safe care. Relates to ability to accurately assess and manage patients. May relate to a lack of awareness, knowledge, training or supervision.	25	48
Clinical uncertainty	Explicit or implicit uncertainty or variance in practice. May relate to decision-making, processes of care, deviance from recommendations in guidelines, complexity in clinical case.	25	43
Recognition of risk or problems	Issues relating to identification of increased risk of complications or need for surveillance/action. Ability to perceive problems.	31	89
Theoretical processes of care_pre-eclampsia	Pathways or processes for how patients with high blood pressure or pre-eclampsia move between levels within or between health facilities, in theory (as opposed to what actually happens/is observed).	11	22
Quality issues		0	0
Adherence to guidelines	Examples of how care relates to national or local guidelines	22	55
Communication between staff	How, when and why staff communicate with each other	23	66
Communication with patients and family	Any communications or verbal interactions with patients or their families, either directly observed or described. The content of, why, when or how, communication with patients and their attendants.	24	89
Conflict between staff	Includes disagreements, differences in opinions or different approaches to problems or situations	10	15

Robbins T, *et al. BMJ Open* 2023; 13:e061500. doi: 10.1136/bmjopen-2022-061500

Name	Description	Files	References
Conscientiousness	Healthcare professionals being diligent and willing to work efficiently	22	41
Consent	Permission to medical treatments, examinations or procedures. May include examples of consent taken/given or not taken/given. May be verbal or written or sometimes implied e.g holding out arm for a blood pressure check.	12	18
Detection or surveillance_pre-eclampsia	Any observations relating to detection of pre-eclampsia including BP monitoring, screening urine or blood tests for end organ damage.	22	54
Quality of documentation	use of written records to note down care given including monitoring (checking vital signs), investigations, treatments, diagnoses, patient details.	27	98
Timely management_pre-eclampsia	Observations or descriptions of process or sequence of care relating to pre-eclampsia, factors relating to reducing or contributing to delays in care for pre-eclampsia or management of high blood pressure.	6	13
Transparency and accountability	Observations or descriptions relating to (un)ethical professional practice, recognition of errors, appropriate action taken. Relates to open communication and good governance. Transparency relates to ensuring information is available to patients and management, to protect patient safety. Accountability to ensure providers/facilities are held responsible for actions or consequences of errors.	19	50
Structural or systems barriers and facilitators		0	0
Access to medications	Issues relating to availability or lack of medications within hospital pharmacy. Relates to organisation with pharmacy, procurement, supply chains and financing.	9	21
Availability and awareness of guidelines	relates to providers/healthcare workers awareness of relevant clinical guidelines and their availability in clinical areas for use	13	26
Availability and utilisation of WASH	Water And Sanitation Hygiene. Availability, access and utilisation of water and sanitation equipment including soap.	14	22
Cleanliness	General cleanliness of rooms and facility. May include factors relating to patient/family behaviour such as disposal of general waste. And/or behaviours or practices of clinical or cleaning staff.	14	30
Electricity supply	Lack of access or supply of electricity	6	13
Inconsistency in directives or care	Failure to standardised procedures. May be due to changes in advice from	16	27

Robbins T, *et al. BMJ Open* 2023; 13:e061500. doi: 10.1136/bmjopen-2022-061500

Name	Description	Files	References
	management, differences in understanding or interpretation of guidance or rules or unclear guidance.		
Organisation of services	Where distinct services are offered and which cadres of staff cover these services.	15	26
Payment for maternal care services	Details of payments required or made by patients or their families for services not provided for free at the point of care. Out of pocket payments.	16	33
Staffing	Details of numbers, cadres of staff providing care, availability out of hours and access to specialists outside daytime hours or on weekends. Includes issues related to understaffing/lack of required staff numbers for safe or effective practice.	23	62
Support and training for staff	References to professional development or learning and support or need for any of these.	16	57
Triage process and initial management	Any observations or descriptions relating to when patient's first arrive in hospital, initial assessment and prioritisation of patients/conditions. Who does this, where, how. Included details of initial place of care, cadres of health work providing care, initial investigations, assessment and any treatments given immediately.	16	48
Workload	Factors influencing the amount of work and productivity of the facility. Related to utilisation of services and workforce structures and numbers.	22	67
2.0 Improvisation_Analysis 1	Making do or tinkering in attempt to mitigate systemic constraints	0	0
Appropriateness of referrals	Factors relating to whether referrals are timely/late AND/OR appropriate or inappropriate in terms of clinical need/level of care at receiving facility AND/OR appropriate communication to receiving hospital in advance	10	16
Availability of clinical investigations	included any lab tests, ultrasound, etc.	12	18
Availability of equipment and materials	Availability of materials and essential equipment e.g gloves, sheets, resuscitation equipment. EXCLUDES medication	28	85
Blood transfusion	Issues relating to availability, access to, planning, supply of blood and blood products including plasma/FFP.	12	29
Clinical decision-making	How, when and where clinicians make decisions about management of patients. e.g whether require admission, involving colleagues/team decision-making	26	53
Decision re mode of delivery	Factors influencing how decisions are made about how to deliver babies e.g	8	12

Name	Description	Files	References
	Caesarean delivery versus vaginal delivery. Also includes spontaneous vaginal delivery (SVD) versus instrumental birth (ventouse or forceps delivery).		
Decision re timing of delivery	Factors influencing when clinicians may recommend birth, if women present with problems or require intervention.	11	22
Efficiency	How resources, time or people are used to reduce waste. Related to provision of quality care.	13	20
Escalation of care	Processes of referral on to other clinicians or higher level facility, where more skills, knowledge or resources are required in making a clinical assessment or giving treatment.	31	89
Family support	Support from partners or wider family, may be emotional or practical support. Relates to enabling access and provision of appropriate, quality care in terms of patient centred care, timely care if support from family etc.	18	45
Fetal or baby care and monitoring	included observed processes of care or descriptions of how fetal monitoring is carried out including assessment of heart rate or obstetric ultrasound	13	42
Observed processes of care_pre-eclampsia or severe hypertension	Includes observed processes of care for women with severe hypertension (160/110mmHg). Examination, ordering investigations, prescribing medication, admission criteria.	16	36
Observed processes of care_magnesium sulphate	More detailed accounts of magnesium sulphate administration or use including dosing, length of treatment, reasons for initiation or discontinuing.	10	15
Reassessment	rechecking vital signs, clinical condition or investigations. Relates to timely detection of problems, detection of worsening problems, need to escalate and provision of quality care.	18	36
Physical organisation of care	Physical locations within the hospital, designated to particular services and how these are organised.	18	41
Patient flow	the movement of patients through the facility/different stages of care from triage/admission to discharge	5	16
Precarity	Uncertainty or insecurity in care given, may be due to miscommunication or lack of adherence to recommended/evidence-based care.	21	34
Prioritisation	Processes of deciding on relative importance of tasks or problems.	9	19

Robbins T, *et al. BMJ Open* 2023; 13:e061500. doi: 10.1136/bmjopen-2022-061500

Name	Description	Files	References
Privacy	Issues relating to provision of respectful care, including offering to cover patients during examination, providing space for using toilet facilities/bed pan in a space without others around.	18	57
Roles and responsibilities	Descriptions or observations relating to professional roles and responsibilities including particular tasks and duties undertaken.	23	74
Safety	Any issues relating to patient or staff safety including structural issues or processes of care. Includes observations or descriptions of unsafe practice relating to administration of drugs, use of medical devices, unsafe injection practices, hygiene and infection control. Includes appropriate disposal of clinical waste.	20	47
Theoretical processes of care_general	Pathways or processes for how patients move between levels within or between health facilities, in theory (as opposed to what actually happens/is observed). RELATING TO ANY CONDITION	11	17
3.0 Trust and support_Analysis 1		0	0
Acceptance of treatment or advice	Discussion or views about whether patients and/or families accept treatments or management as advised by caregivers. May involve decisional conflict.	20	49
Authority structures	Interactions or examples of hierarchy within the care system. Included both health professionals e.g midwives, nurses, IESO, obstetrician, medical director and auxiliary staff e.g cleaners, lab technicians, and also decision-makers e.g women, partners	16	24
Availability of specialist staff	Access and availability. Includes staff with any specialist skills e.g doctors (obstetrician/gynaecologist), laboratory staff	16	33
Barriers and facilitators to follow up	Challenges that hinder mothers attending for follow up either routine or if notice danger signs/have a problem AND/OR factors that can improve attendance at facility, acceptance of advice or treatments	14	36
Communication with patients and family	Any communications or verbal interactions with patients or their families, either directly observed or described. The content of, why, when or how, communication with patients and their attendants.	24	89
Creating community awareness	Health promotion activities to improve knowledge and awareness of danger signs during pregnancy, birth and postnatally.	14	43
Defensiveness	Behaviour observed or described relating to being anxious or to avoid potential	10	18

Name	Description	Files	References
	criticism.		
Feedback to referring health centres	System or processes to allow information to go back to referring facilities including clinical outcomes, appropriateness of referrals, appropriateness of initial triage/management, documentation, staff accompanying on referral	7	15
Inertia	Tendency to do nothing/not take action when required despite lack of barriers.	13	25
Maintenance of equipment	includes charging equipment, replacement of batteries or broken parts, servicing equipment etc.	10	18
Neglect	Failure to provide quality care or abandonment of patient needs	13	20
Patient concerns	Any issues relating to anxieties or problems expressed by patients or described by those caring for them. Relates to access and quality of care.	23	60
Professional satisfaction	Health workers feelings about their roles, how they are valued, thoughts about pay/compensation for work and incentives	7	16
Self-appraisal of skills	Assessment by individuals of themselves/their own skills/ability or of the facilities within which they work. Self-reflection.	8	19
Team working	Collaboration between professionals to perform duties including examples of joint decision making (included examples of good or poor team working)	11	26
Trust in resources	Relating to confidence in either physical/material or human resources.	15	18
Undermining	Observations or descriptions of behaviour that diminishes power of an individual to act/carry out their responsibilities.	7	7
Use of traditional medicines	Any issues relating to pregnant women using traditional medicines and how this relates to their care in health facilities.	7	13
Women's experience of care or services	includes their perceptions about quality of care in hospital, continuity of carer, information provided to them, how professional communicated, if they were counselled appropriately (general impressions that may not fit into other codes)	7	25

Robbins T, *et al. BMJ Open* 2023; 13:e061500. doi: 10.1136/bmjopen-2022-061500
