# Supplementary material for: Understanding challenges as they impact on hospital-level care for pre-eclampsia in rural Ethiopia: a qualitative study
Source: BMJ Open. 2023 Apr 17;13(4):e061500. doi: 10.1136/bmjopen-2022-061500 (PMC10111927; doi:10.1136/bmjopen-2022-061500)
Supplement: Author's manuscript [file bmjopen-2022-061500.draft_revisions.pdf]

BMJ Open is committed to open peer review. As part of this commitment we make the peer review history of every article we publish publicly available.

When an article is published we post the peer reviewers' comments and the authors' responses online. We also post the versions of the paper that were used during peer review. These are the versions that the peer review comments apply to.

The versions of the paper that follow are the versions that were submitted during the peer review process. They are not the versions of record or the final published versions. They should not be cited or distributed as the published version of this manuscript.

BMJ Open is an open access journal and the full, final, typeset and author-corrected version of record of the manuscript is available on our site with no access controls, subscription charges or pay-per-view fees (http://bmjopen.bmj.com).

If you have any questions on BMJ Open's open peer review process please email info.bmjopen@bmj.com

BMJ Open

Understanding challenges as they impact on hospital level care for pre-eclampsia in rural Ethiopia: a qualitative study

Journal:	*BMJ Open*
Manuscript ID	bmjopen-2022-061500
Article Type:	Original research
Date Submitted by the Author:	28-Jan-2022

Complete List of Authors:	Robbins, Tanya; King's College London, Department of Women and Children's Health<br>Shennan, Andrew; King's College London, Department of Women and Children’s Health, School of Life Course Sciences<br>Sandall, Jane; King's College London, Department of Woman and Children's Health<br>Eshetu Guangul , Tigist; Addis Ababa University College of Health Sciences, Centre for Innovative Drug Development and Therapeutic Trials for Africa (CDT-Africa)<br>Demissew, Rahel; Addis Ababa University, Department of Obstetrics and Gynecology<br>Abdella, Ahmed; Addis Ababa University College of Health Sciences, Department of Obstetrics & Gynaecology<br>Mayston, Rosie; King's College London, Department of Global Health and Social Medicine<br>Hanlon, Charlotte; King's College London, Institute of Psychiatry, Psychology and Neuroscience, Health Service and Population Research Department, Centre for Global Mental Health; Addis Ababa University, Centre for Innovative Drug Development and Therapeutic Trials for Africa (CDT-Africa), College of Health Sciences

Keywords:	OBSTETRICS, Hypertension < CARDIOLOGY, QUALITATIVE RESEARCH, Change management < HEALTH SERVICES ADMINISTRATION & MANAGEMENT, Health policy < HEALTH SERVICES ADMINISTRATION & MANAGEMENT, Organisation of health services < HEALTH SERVICES ADMINISTRATION & MANAGEMENT

SCHOLARONE™ Manuscripts

I, the Submitting Author has the right to grant and does grant on behalf of all authors of the Work (as defined in the below author licence), an exclusive licence and/or a non-exclusive licence for contributions from authors who are: i) UK Crown employees; ii) where BMJ has agreed a CC-BY licence shall apply, and/or iii) in accordance with the terms applicable for US Federal Government officers or employees acting as part of their official duties; on a worldwide, perpetual, irrevocable, royalty-free basis to BMJ Publishing Group Ltd ("BMJ") its licensees and where the relevant Journal is co-owned by BMJ to the co-owners of the Journal, to publish the Work in this journal and any other BMJ products and to exploit all rights, as set out in our licence.

The Submitting Author accepts and understands that any supply made under these terms is made by BMJ to the Submitting Author unless you are acting as an employee on behalf of your employer or a postgraduate student of an affiliated institution which is paying any applicable article publishing charge ("APC") for Open Access articles. Where the Submitting Author wishes to make the Work available on an Open Access basis (and intends to pay the relevant APC), the terms of reuse of such Open Access shall be governed by a Creative Commons licence – details of these licences and which Creative Commons licence will apply to this Work are set out in our licence referred to above.

Other than as permitted in any relevant BMJ Author's Self Archiving Policies, I confirm this Work has not been accepted for publication elsewhere, is not being considered for publication elsewhere and does not duplicate material already published. I confirm all authors consent to publication of this Work and authorise the granting of this licence.

For peer review only

Title

Understanding challenges as they impact on hospital level care for pre-eclampsia in rural Ethiopia: a qualitative study

Corresponding author details

Tanya Robbins

Department of Women and Children's Health

School of Life Course Science

King's College London

10th Floor, North Wing

St Thomas' Hospital

Westminster Bridge Road

London SE1 7EH

tanya.robbins@kcl.ac.uk

Authors

Tanya Robbins [a] tanya.robbins@kcl.ac.uk

Andrew H Shennan [a] andrew.shennan@kcl.ac.uk

For peer review only

For peer review only - http://bmjopen.bmj.com/site/about/guidelines.xhtml

Jane Sandall [a] jane.sandall@kcl.ac.uk

Tigist Eshetu Guangul [b] tigi.eshetu@yahoo.com

Rahel Demissew Gebreyohannes [c] raheldemissewgy@gmail.com

Ahmed Abdella [c] ahmedamu@yahoo.com

Rosie Mayston [d] rosie.mayston@kcl.ac.uk

Charlotte Hanlon [e, f] charlotte.hanlon@kcl.ac.uk

Affiliations

[a] Department of Women & Children's Health, School of Life Course Sciences, King's College London, St Thomas' Hospital, London, UK

[b] Centre for Innovative Drug Development and Therapeutic Trials for Africa (CDT-Africa), College of Health Sciences, Addis Ababa University, Addis Ababa, Ethiopia

[c] Department of Obstetrics & Gynaecology, College of Health Sciences, Addis Ababa University, Addis Ababa, Ethiopia

[d] Department of Global Health and Social Medicine, King's College London, London, UK

[e] Centre for Global Mental Health, Health Service and Population Research Department, and WHO Collaborating Centre for Mental Health Research and Training, Institute of Psychiatry, Psychology and Neuroscience, King's College London, London, UK

For peer review only

[f] Department of Psychiatry, WHO Collaborating Centre for Mental Health Research and Capacity-Building, School of Medicine, College of Health Sciences, Addis Ababa University, Addis Ababa, Ethiopia

Word count

4124 words

Abstract

Objective

To explore hospital level care for pre-eclampsia in Ethiopia considering the perspectives of those affected and healthcare providers, in order to understand barriers and facilitators to early detection, care escalation and appropriate management.

Setting

A primary and a general hospital in southern Ethiopia.

Participants

For peer review only

For peer review only - http://bmjopen.bmj.com/site/about/guidelines.xhtml

Women with lived experience of pre-eclampsia care in hospital, families of women deceased due to pre-eclampsia, midwives, doctors, integrated emergency surgical officers and healthcare managers.

Results

This study identified numerous systemic barriers to provision of quality, person-centred care for pre-eclampsia in hospitals. Individual staff efforts to respond to maternal emergencies were undermined by a lack of consistency in availability of resources and support. The ways in which policies were applied exacerbated inequities in care. Staff improvised as a means of managing with limited material or human resources and knowledge. Social hierarchies and punitive cultures challenged adequacy of communication with women, documentation of care given and supportive environments for quality improvement.

Conclusions

Quality care for pre-eclampsia requires organisational change to create a safe space for learning and improvement, alongside efforts to offer patient-centred care and ensure providers are equipped with knowledge, resources and support to adhere to evidence-based practice.

For peer review only

Strengths and limitations of this study

- Observational data, triangulated with in-depth interviews alongside relevant health policy and clinical guidelines, enabled a holistic examination of processes and quality of care and the factors challenging them.
- A rapid ethnographic approach enabled feedback of key practical findings from our observational work to staff and district level stakeholders, in a timely way, informing quality improvement initiatives as part of wider health system strengthening efforts. This also offered an opportunity for member checking, ensuring our observations reflected real-world challenges faced by providers.
- Longer periods of observation may have revealed further useful insights, however recurring themes were evident in field notes.

Introduction

Poor quality maternal care in hospitals contributes significantly to avoidable deaths. Pre-eclampsia is a leading cause of maternal and perinatal mortality and morbidity globally.[1] The greatest burden is borne by low- and middle-income countries (LMICs).[2] There is a dearth of robust national data on incidence, particularly in LMICs. It is estimated that 2-5% of pregnancies globally are affected by pre-eclampsia.[3 4] In Ethiopia, 19% of maternal deaths are attributed to hypertensive disorders of pregnancy (HDP) compared to 14% worldwide.[1 5] Good quality care makes a difference to outcomes; the majority of deaths due to pre-eclampsia are preventable. Less than one in a million women delivering in the UK now dies due to pre-eclampsia, compared to an estimated 783 deaths per million in Ethiopia.[6]

Internationally there is a lack of consensus concerning classification, diagnosis and management of pre-eclampsia.[7] Controversy remains over inpatient versus outpatient management, target blood pressure and timing of delivery for preterm pre-eclampsia.[8] However, there is evidence to support prophylaxis with low-dose aspirin and supplemental calcium if dietary intake is low, treatment of severe hypertension (≥160/100mmHg) and use of magnesium sulphate for eclampsia or pre-eclampsia with severe features.[9-14] Prediction and prevention of pre-eclampsia is an important goal in reducing mortality and morbidity.

For peer review only

However robust health systems and continuity across different levels of the care pathway are needed to support effective implementation of these interventions.

Recent proposals for rethinking models of maternity care to improve quality and equity have called for increased focus on centralised services and shifting all deliveries to or near hospital settings.[15] Justifications included the limitations of obstetric risk stratification, low quality care in primary level settings and challenges of referral if complications do occur. However, to address equity, redesign must be country-led, context specific and co-designed with a focus on listening to women and strengthening human resources.[16] As poor quality care emerges as a greater barrier to reducing mortality than insufficient access to services, understanding challenges in secondary care and the experiences of women cared for in hospital is critical.[17] A large, international prospective dataset showed that 61% (28.1% within a central referral hospital) of eclampsia cases in Ethiopia occurred within facilities, highlighting the need to improve quality of care as well as surveillance, early detection and access to appropriate care.[18]

For peer review only

The objective of this study was to develop rich contextual understanding of the processes and experiences of hospital level care for pre-eclampsia in rural Ethiopia to inform efforts to improve quality.

Methods

Study setting

This study was conducted in a primary and general hospital in the Gurage Zone, Southern Nations, Nationalities and Peoples' Region (SNNPR). Ethiopia has an ethnically diverse population of over 112 million, 79% of whom live in rural areas. Government-run health services are structured into three-tiers: primary, secondary and tertiary care. See figure 1. The general hospital at secondary level care serves as a referral center for the primary hospital. In the study setting, maternity services at the primary hospital were provided by midwives, junior doctors referred to as general practitioners (GPs) and integrated emergency surgical officers (IESOs). IESOs are non-medical clinical staff trained to provide emergency surgical care. In addition to these cadres, the general hospital was also staffed by an obstetrician.

For peer review only

Ethiopia has seen significant reductions in maternal mortality ratios over recent decades from 1030 deaths per 100,000 live births in 2000 to an estimated 401 in 2017.[19] However, institutional delivery rates remain low, estimated at 26% in 2016 and only 43% of pregnant women had four or more antenatal care visits in 2019. The Ethiopian Federal Ministry of Health (FMoH) produces strategies to improve all levels of the health system set out in five-yearly Health Sector Transformation Plans to guide healthcare delivery.[20] These align with current international commitments such as achieving quality, person-centred, respectful and equitable healthcare.

Figure 1. Ethiopian Health System Structure

Data collection

We conducted a qualitative study between January 2019 and January 2020, using unstructured participant observation, semi-structured interviews and document analysis of hospital guidelines and meeting minutes. Our research team included both an Ethiopian (RD) and a British obstetrician (TR) and an Ethiopian social epidemiology researcher (TE). Two researchers conducted rapid ethnographic observation over 16 days between the primary and general hospital.[21] Observers included a clinician and an Amharic speaker at all

For peer review only

times. We observed all aspects of maternity care relevant to pre-eclampsia including spending time on the labour ward, in the emergency department, antenatal clinic, inpatient wards and in the operating theatre. Ethical challenges about when and how to intervene when questionable practices were observed, were discussed between clinical members of the research team. Clinical researchers assisted in life-threatening situations. Where staff explicitly asked for assistance with non-critical cases they were encouraged to continue with routine practice. Contemporaneous field notes were taken and discussed between researchers encouraging reflexive practice. 21 formal in-depth semi-structured interviews were conducted. We purposively sampled 10 staff members (two midwives, three IESOs, four doctors including an obstetrician and a district health manager), eight women with lived experience of pre-eclampsia and three family members of women who had died due to pre-eclampsia. Interviews were conducted in Amharic, audio-recorded, transcribed and translated into English. Transcripts were cross-checked by Ethiopian research co-ordinators for accuracy.

Findings from the participant observation were fed back to staff via a meeting to inform later stages of a wider project on health system strengthening. Minutes from this meeting along

For peer review only - http://bmjopen.bmj.com/site/about/guidelines.xhtml
For peer review only

with national guidelines relating to care for pre-eclampsia were also used as data sources to triangulate findings and provide relevant context.

Clinical care guidelines in use at the time of the study included: FMoH Management Protocol on Selected Obstetric Topics 2010, Standard Treatment Guidelines 2014 (STG) for Primary and General Hospitals and a Comprehensive Emergency Obstetric and Newborn Care Training Manual. The FMoH guidelines advocated for four focused antenatal care (ANC) visits emphasising quality rather than quantity. Usual care for women with low-risk pregnancies was provided at health centres and the other visits conducted by health extension workers (HEWs) at health posts close to women's homes. Pregnant women identified to have risk factors including amongst others, previous pre-eclampsia, chronic hypertension, diabetes or renal disease were, according to national guidelines to be referred to higher level care or offered closer follow up.

For peer review only

Data analysis

We undertook thematic analysis using an inductive approach[22]. Field notes, interview transcripts and the minutes of the meeting were uploaded into NVivo12 software. Data were read and re-read; and initial codes were generated, reviewed and refined by TR, TE and CH.

All data were then recoded by TR and themes generated and reviewed with CH and RD. Themes and subthemes were checked against the data to examine relationships between them.

Ethical approvals

All methods were carried out in accordance with relevant guidelines and regulations including the Declaration of Helsinki. Ethical approvals were granted by Addis Ababa University (026/18/Psy) and King's College London (LRS-17/18-5526). Institutional level consent was given by hospital management for observational data collection. Interview participants were required to give individual consent.

Patient and public involvement

The design of this study focussed on examining the lived experience of women affected by pre-eclampsia and their families. We included formal and informal interviews with women. Our research questions were informed by endeavouring to understand the priorities and experiences of patients. They were not involved in the planning, design or analysis of this study.

For peer review only

Results

Women and healthcare providers described significant systemic barriers to the provision of quality care. Basic structural necessities such a running water, soap and electricity were intermittently unavailable. This was particularly evident at the primary hospital. During four days of observation at the primary hospital there was no running water. There were no sinks in any of the inpatient wards or clinical areas, including the infection prevention room at the primary hospital.

Our analysis revealed contextual challenges that women and professionals faced, within which they sought to receive or provide quality care for pre-eclampsia. We present three main themes: inconsistencies and inequity, improvisation and making do, and trust and support.

Theme I: Inconsistencies and inequity

Key guidelines identified by staff as informing care for pre-eclampsia at hospital level were acknowledged by some professionals as being outdated and were adhered to variably. Women admitted to hospital with pre-eclampsia were often managed according to the national guideline but not consistently. Variability in clinical practice related to incomplete

For peer review only

clinical assessments, including not eliciting or documenting significant risk factors such as a history of eclampsia and previous stillbirth, and a lack of knowledge, for example, of established thresholds of severe hypertension. Guidelines and practice were ambiguous relating to the management of antenatal women presenting with, or at risk of eclampsia, who were preterm and whose blood pressure (BP) normalised. Staff reported that women were discharged prior to delivery if their BP was controlled with antihypertensives. Some medical staff reported they used diazepam to control seizures despite the availability of magnesium sulphate and guidelines stating this as the drug of choice.

Women described concerns about competence and safety. Some were particularly worried about the danger this posed to more vulnerable, less educated women who may be unable to advocate for themselves. A family member of a woman who died described conflicted feelings, both troubled by the care received whilst recognising providers' motivations were honourable.

> *"I have seen a lot there, we or you may read English a little and yes, your parents may read Amharic but how about those women who do not know anything. Yes, mistake a lot there, especially practitioners who provide medicine at resting room*

For peer review only

after surgery if they are not very careful, I do not know who will make them accountable."

(Woman with previous pre-eclampsia, M28)

"In this hospital, they don't cooperate. We are grumbling because of them. We are blaming the hospital for her death…Yes, her body refused to circulate someone else's blood and get swelled….I am not better than the health professionals; they were trying to save her not to kill her…yes…I am thinking this was because I am hurt not because I am better than the health professionals."

(Family of deceased woman, M26)

Relevant to the variation in clinical practice and competence, were inadequate and inequitable access to training opportunities and professional development as described by providers. Some staff linked this to poor support from the hospital administration, whilst others perceived a professional culture lacking in motivation to pursue independent study and continuing professional development.

For peer review only

Inconsistencies in how policies were implemented were also observed and reported. This variation may have been intended to ameliorate inequities in the system but also exacerbated them. National level policy for free maternal care was implemented differentially dependant on clinical situation. In this study setting, women admitted to hospital with complications such as pre-eclampsia, requiring inpatient care but not delivery, were required to pay for services. Women attending purely for delivery received free care. Whereas women with potentially life-threatening complications requiring several medications, longer inpatient stays and at higher risk of delivering preterm infants in need of neonatal care, who were able to, were required to pay. However, women from low socio-economic status households could be considered for exemption by the hospital management or could access free care if community-based health insurance had been rolled out in their area. Nonetheless, out-of-pocket payments for antihypertensive drugs were common. Financial barriers were observed to present tangible challenges for women with potentially serious complications related to pregnancy. Clinicians were not always aware of policy or how to apply it in practice.

Case vignette

For peer review only

A recently postpartum woman presented to the emergency department with significant pitting oedema to bilateral lower limbs. She was reviewed by the doctor on-call and laboratory investigations were requested. She attended the lab but was asked for 160 birr (£2.80) which she was unable to pay. She did not have a community-based health insurance card. The GP asked her to go back to her village, to collect the insurance card, then to visit the local health centre and ask for referral back to the hospital for a full assessment. She did not return to the hospital that day.

> *"It is not free whether she is here or discharged. The hospital will fulfil things required for delivery, like gloves and others. The mothers may not buy the medication since they may not have money; there are mothers who don't have any money. Thus, even if we told them to buy it, they might not buy the medication… like antihypertensive, they have to pay…Yes, she has to purchase it. If methyldopa or so on is prescribed to her at ANC, she has to purchase it from her own pocket… It would have been good if it is possible to make laboratory services and medications prescribed for the mothers free as well. They would have come if it is free"*

(Midwife, M11)

For peer review only

"The problem that we have in this hospital is, sometimes after deciding to admit them, there is some kind of problem on the payment system. For example, if a pregnant woman is close to due date and or going to give birth after some days and is going to be admitted, she is not going to pay. But if she has been admitted not for delivery, she would pay. There are these kinds of unclear rules. If it is free for one person, it should be free for all."

(Senior Clinician, M32)

Additional examples of how inconsistencies and inequity presented are summarised in table 1.

Table 1. Theme I: Inconsistencies and inequity

Inconsistencies and inequity	Verbatim quotes
Clinical knowledge of established thresholds for severe hypertension and appropriate management	*"She will be given magnesium sulphate (MgSO4 if her systolic BP is above 180 or diastolic above 110, we will give her hydralazine to drop the elevated BP."* (Midwife, M11)
A limited understanding of the aetiology of pre-eclampsia reflected in the advice given to women regarding their diagnosis	*"They used to tell me not to drink soft drinks, tea and the like. And that I don't have to use too much salt and need to decrease my fluid intake until the blood pressure goes down."* (Woman with previous pre-eclampsia, M37)
Ambiguous guidelines and practice relating to the management of preterm pre-eclampsia with severe features	*"Q: She came with eclampsia; under what condition you will decide and discharge her?*<br>*A: When her BP become normal, and her protein level reduces. It will become normal when we give*

For peer review only

when the blood pressure normalises following treatment with anti-hypertensive medications	*them antihypertensive. There are many mothers who are discharged."* (Midwife, M11)
Inconsistencies in triage of pregnant women relating to a lack of skills, knowledge or experience	*"One of the things that needs improvement is, developing the health professionals' skill and knowledge. Most of the problems are happening because of lack of knowledge. Most things are happening by not considering as a problem when actually problem has happened to mothers. Therefore, by improving the awareness we can change many things."* (Senior Clinician, M32)
Inappropriate triage may result in failure to recognise risk, escalate and manage appropriately	*"Sometimes triage might not be done appropriately, or due to limited knowledge and skill gap, the general practitioner might miss some cases. But once the surgeon is involved, he will be fully responsible."* (Medical Director, M49)
Inequitable access to training opportunities and support for professional development	*"Most of the time, there are no trainings. The process of informing new information and participating the health professionals on training is very poor. This is one of the problem of administrators. Sometimes there are various types of training opportunities for their health professionals or the administrators will train the health professionals on areas where they believe there is a gap. They may also forbid the health professionals that was requested to get the training by paper from the high-level officials. This is the main problem of the administrators in our health institutions. The training does not have to be for getting the per diem rather it has to be used to updating the knowledge and to apply what you have got from the training. The way of administrators supporting this is very poor."* (IESO M48)

For peer review only - http://bmjopen.bmj.com/site/about/guidelines.xhtml

Perceived lack of professional motivation for self-led learning	*"The first thing is when there are such trainings, our society as a whole have a very poor reading habit. People don't sit down and read the guideline."* (Medical Director, M49)

Theme II: Improvisation and making do

Improvisation emerged as a means of managing or making do with limited resources, both material and human, within both hospitals. This centred around a lack of equipment, medications, staff and support. The barriers and facilitators to early detection, care escalation and appropriate management of pre-eclampsia are described in table 2.

BP monitors were lacking in both hospitals. Staff made-do by sharing, negotiating use between departments or improvising with inadequate equipment such as a plastic training stethoscope used with a sphygmomanometer. At the primary hospital, there was no BP monitor available in the antenatal clinic. If midwives felt a BP check was needed, the woman was escorted to the ward to have this done.

Midwives at the general hospital made do with one semi-functioning cardiotocograph machine between six beds in the "first stage room" and two delivery beds. Midwives could only assess the current heart rate pattern, or sit with women and observe in real-time, as

For peer review only

paper was not available to record. Improvisation with regards to fetal monitoring for high-risk woman was common and related to the lack of resources, but also a lack of knowledge of normal parameters and difficulties interpreting fetal heart rate patterns. Failure to recognise abnormal patterns was observed, which led to delays in taking action, either to expedite delivery or escalate. No fetal monitoring took place in the second stage of labour, regardless of earlier concerns. The reasons for this were difficult to ascertain but this was an established culture of practice. On occasions, the management of suspected fetal distress at the general hospital involved the use of 40% dextrose infusions to the mother. These practices may have been related to a lack of specific guidance for midwives on the management of fetal distress.

Assessment of antenatal women presenting with reduced fetal movements close to term, were inadequate. The fetal heart was auscultated to confirm presence. Women were not offered blood pressure or urine screening, assessment of symphysial fundal height or a growth scan. At the primary hospital no continuous fetal monitoring was available and a diagnosis of presumed fetal distress was made following auscultation of decelerations using a fetoscope.

For peer review only

For peer review only - http://bmjopen.bmj.com/site/about/guidelines.xhtml

Precarious practices were observed such as the use of fundal pressure during the second stage of labour when providers lacked confidence to perform assisted delivery.

Families offered frequent support to plug gaps in the system, collecting patient notes, requesting blood tests, clearing buckets of urine and blood on labour ward, procuring drugs and equipment from private pharmacies when not available in labour, and providing support for women in labour when allowed.

Table 2. Barriers and facilitators to early detection, care escalation and appropriate management of pre-eclampsia in hospital

Hospital level care for pre-eclampsia	Barriers	Facilitators
Early detection and recognition of risk	• A lack of continuity of care and linkage between general outpatient department (OPD) clinics where women with chronic hypertension, diabetes or renal disease may be managed and high-risk ANC clinic<br>• Unclear guidelines for midwives, IESOs and doctors regarding outpatient management of women referred from lower levels of care with risk factors for pre-eclampsia<br>• A lack of BP monitors in clinical areas where pregnant women were cared for	• Regular blood pressure monitoring was observed for women admitted with severe hypertension (although not always documented)

For peer review only

Escalation of care	• Staff responsible for triaging pregnant women were not always competent, experienced or supported enough to escalate care appropriately<br>• Poor documentation of clinical history, risk factors, examination and care given challenged communication between staff and different levels of the care pathway	• Referrals from lower levels of care (health centres and primary hospital) were reported to be frequent (although reported by staff as not always necessary)<br>• A clear professional hierarchy was described by staff, indicating they knew who they could refer on to
Appropriate management	• National guidelines were not evidence-based or up to date or were unclear, recommending precarious or potentially unsafe practices<br>- Management of hypertension (explicit advice not to treat non-severe hypertension ≥140/90 to 159/109mmHg, lack of timely management of severe hypertension -advice to wait one hour)<br>- Timing of delivery (recommend delivery when cervix favourable for “mild pre-eclampsia”, advise avoiding post-term pregnancy but no explicit recommendation for delivery at 37 weeks)<br>• A lack of fluid balance monitoring was observed, and injudicious use of intravenous fluids concurrently with furosemide without a clear indication<br>• Timing of antenatal steroids for fetal lung maturation (given to women with preterm pregnancy and hypertension even when delivery was not planned)	• Magnesium sulphate was available, and doses prescribed according to national and international guidelines<br>• Guidelines recommend delivery within 24 hours of onset of symptoms in “severe pre-eclampsia” and within 12 hours of eclampsia.

For peer review only

	• Unclear and inconsistent processes for induction of labour, increasing the risk of caesarean section.<br>• Poor documentation of clinical care led to a lack of transparency and accountability<br>• Minimal counselling regarding future risks including in subsequent pregnancies, increased risk of cardiovascular disease and stroke, and no guideline to support this practice	

Theme III: Trust and support

Communication skills and styles varied between individuals with examples of kind, compassionate interactions, professionals checking women's understanding and providing reassurance. However, a culture of informed consent and shared decision-making was not widespread. Women were frequently subjected to vaginal examinations in an open ward without privacy. There were observed examples of abrasive interactions and failures to take women's concerns seriously. Decisions about termination of pregnancy for women with early onset pre-eclampsia were made without explanation to women and were not documented in hospital notes. These interactions were described as "oral reviews". Staff were frequently observed carrying out clinical activities without documenting including performing vaginal examinations, ward rounds and administering drugs. Some staff reported this related to care being given outside prescribed protocols, for example, the timing of examinations during

For peer review only

labour. Poor quality documentation was also linked to a punitive culture and fear of being blamed for adverse outcomes. Strategies to improve documentation were in progress including a documentation quality audit.

Allowing women family support during labour was inconsistent and limited by structural constraints such as the lack of privacy and space in the labour ward. However, relating back to the initial theme, inequitable application of the rules left women who were less able to advocate for themselves alone and lacking support, whilst others were allowed family members with them. Staff talked about the challenges they felt affected their ability to provide respectful care including workload and not feeling respected themselves.

There were examples of low staff satisfaction reported by women, affecting quality of care. Staff reported low level of professional support from the hospital administration and a sense of moral duty driving their commitment to work. Suggestions about how to support and retain staff reflected a need to develop more nurturing systems with opportunities for feedback to clinicians, both positive and negative. The theme of trust and support is further illustrated in table 3.

For peer review only

Table 3. Theme III: Trust and support

Theme III: Trust and Support	Verbatim quotes
Women experienced or witnessed lack of respectful care or staff attitudes that presented barriers to quality care	*"But there are also doctors who think they know everything, but they are below doctors. To your surprise, you see it many times, those who insult, who say you did not follow here so go away. But it has no problem. She enters for delivery after signing for the risk, right. She will be responsible for everything that could happen to her. But saying go away while she is about to give birth and because she did not follow there, I have seen a lot."* (Woman with previous pre-eclampsia, M28)
Communication with women affected what they understood about their diagnosis and their trust in professionals and the care received	*"I am still not sure whether I do have or don't have the hypertension at that time. Finally, at the hospital I wasn't so sure whether I have hypertension or not but at the hospital, they said, "It is very much elevated, and she might get into a bad condition if we are waiting more than this". Then they made me sign and performed the surgery and I gave birth. That's all about it. I didn't understand whether they did that purposely to intimidate me or whether I exactly do have the hypertension"* (Woman with previous pre-eclampsia, M34)
Doubt and anxiety about their diagnosis led to some women searching for alternative opinions	*"Yes, it was at the hospital. I went three places to check my blood as a matter of fact… they told me to wait and I went to other places in the meantime to get my BP checked… When I checked there, the number was the same. So, I returned back to the hospital because I was told to remain there. I was very nervous."* (Woman with previous pre-eclampsia, M25)
Staff felt challenged to provide compassionate and respectful care	*"Due to the labour pain labouring mothers are aggressive sometimes which makes our job a little bit complicated while giving the service. At this moment it is difficult to practice the CRC [compassionate respectful care] as we hoped to. But you have to minimize your own issue and try to help the*

For peer review only

For peer review only - http://bmjopen.bmj.com/site/about/guidelines.xhtml

	mother and her child. We should take care of the child regardless of the situation and the protocol sometimes." (Head midwife, M33)
Women reported staff were dissatisfied and this affected quality of care and communication	*"I hated many of the women. I am busy and I work here unwillingly [the midwife said]. It is her work, right. She gets paid, right. No one is doing one's job happily. She has to communicate peacefully even if she doesn't like the woman's blood or waste."* (Woman with previous pre-eclampsia, M28)
Staff did not feel supported but were motivated to work by a sense of duty	*"Support is very low. You work for your conscience. There are a lot of problems on the administration side. You just work because of feeling responsible."* (IESO, M47)
Staff reported the need for more feedback as well as support to improve retention	*"Giving recognition for the tasks that they have accomplished well. Appreciating or congratulating on a job well done and give constructive criticism when the work lacks will be great. If not, that person will be compelled to seek other more satisfying work environments."* (IESO, M47)

Discussion

This study found the provision of quality, person-centred care for pre-eclampsia in two Ethiopian hospitals was challenged by numerous systemic barriers. A lack of consistency in availability of resources, support and how policies were applied exacerbated inequities in care. Variations in clinical competence, knowledge and experience meant staff providing maternity care were not always able to recognise risks or act on them. Outdated, ambiguous guidelines and inequitable opportunities for professional development exacerbated clinical uncertainty and practice not based on evidence. National guidelines specify against the use

For peer review only

of antihypertensives for non-severe hypertension and many women with significant hypertension were not treated. Inadequate documentation did not always reflect clinical risks, drugs administered, or care given. Poor quality documentation was linked to the provision of care divergent from protocol and fear of blame for poor outcomes. A lack of transparency, accountability and systems for support were observed and reported. Improvisation was used to address shortages in resources and a lack of clear guidance. This affected quality of care, particularly with regards to fetal monitoring and neonatal care. Staff reported a need for more support, particularly from hospital management, and training opportunities.

Strengths and Limitations

This study offers an ethnographic account of care for women with pre-eclampsia in hospital in rural Ethiopia. Observational data, triangulated with in-depth interviews alongside relevant health policy and clinical guidelines, enabled a holistic examination of processes and quality of care and the factors challenging them. We explored diverse perspectives including women affected by pre-eclampsia, families of women who did not survive, healthcare providers and managers. This approach allowed a deeper understanding of the context of care including clinical, sociocultural and bureaucratic aspects. Our analysis was informed by

For peer review only

participants descriptions and recollections of care they gave or received, as well as observed explicit and tacit actions and interactions. We used the guidelines to contextualise our observations and the accounts of care.

Conducting a rapid ethnography offered both advantages and disadvantages. We were able to feedback key practical findings from our observational work to staff and district level stakeholders, in a timely way, informing quality improvement initiatives as part of wider health system strengthening efforts. This also offered an opportunity for member checking, ensuring our observations reflected real-world challenges faced by providers. Although longer periods of observation may have revealed further useful insights, recurring themes were evident in field notes. Another limitation was the need for translation for one observer. English was used by staff to document in hospital notes, and all staff spoke English, however interactions with patients and between staff were usually in Amharic. Subtle meaning or nuance may have been lost in translation or interactions misinterpreted. Furthermore, the ‘outsider’ status of a non-Ethiopian team member is likely to have affected behaviour of participants. However, this is a recognised challenge when conducting ethnography and was considered when interpreting findings.[23 24] Staff were friendly,

For peer review only

For peer review only - http://bmjopen.bmj.com/site/about/guidelines.xhtml

cooperative and keen to share their experiences, and often asked observers for advice or feedback.

Interpretation

In our study early detection, care escalation and appropriate management for pre-eclampsia were undermined by a lack of up-to-date guidance based on evidence. Clinical manifestations and conceptualisations of pre-eclampsia are variable. The International Classification of Diseases (ICD-11) has adopted an updated, broader definition of pre-eclampsia to include hypertension and proteinuria or new-onset maternal organ dysfunction, neurological sequelae or fetal growth restriction.[7,25] Inconsistency remains in national and international clinical practice guidelines implementation of the broad definition and the categorisation of ‘severe’ pre-eclampsia or pre-eclampsia with severe features.[26] This is important as guidelines for management and recommendations for interventions are linked to classification of the disorder. These inconsistencies were mirrored in the care we observed in this study.

Early prediction and prophylaxis for pre-eclampsia were not a part of routine care in our study site, nor recommended in Ethiopian guidelines. Most Ethiopian women consume

For peer review only

For peer review only - http://bmjopen.bmj.com/site/about/guidelines.xhtml

insufficient calcium, but large regional variation exists.[27] Implementation of interventions requiring early contact may be challenging in a context where first trimester care is rare, particularly at levels of the system where providers are competent and confident to prescribe. However, policy makers should consider evidence-based guidance and international recommendations to support prophylaxis and reduce burden of disease in at risk women.[9 28 29] The updated 2020 national guideline does not address these discrepancies.

Accurate measurement of BP is fundamental to detecting and managing pre-eclampsia. In our study sites both automated devices and mercury sphygmomanometry, which requires skill and training, were used. Few automated devices have been validated in pregnancy or pre-eclampsia and may lead to significant inaccuracies.[30] Accurate and cost-effective devices have been validated in pre-eclampsia and are useful in health systems where low-skilled staff are involved in maternal care.[31-33]

Triage, recognition of risk and care escalation for pre-eclampsia were identified as areas for improvement in our study. In settings where resources are limited, risk prediction models may offer support to staff to target interventions and decision-making around place of care.[34]

For peer review only - http://bmjopen.bmj.com/site/about/guidelines.xhtml

Our findings concur with previous studies reporting gaps in knowledge and practices required for early detection and management of pre-eclampsia in facilities in low-resource settings.[35] Health systems constraints, training, supervision and motivation of staff affect clinical performance and outcomes.

Improvements in external factors such as guidelines and infrastructure, must be mirrored by changes in cultures of care, that support a shared understanding of quality, focus on team competencies and supportive leadership. Previous quality improvement (QI) initiatives in Ethiopia have failed due to lack of understanding and adaptation to local contexts.[36] Understanding formal and informal power structures, considering spheres of influence and creating social and reputational incentives for change may improve focus and engagement. A study aiming to measure and improve quality of intrapartum care, showed that having lead hospitals demonstrate the feasibility of their assessment tool helped to promote its wider acceptance, as other hospitals sought to achieve the national recognition given to the lead hospitals.[37] Participatory approaches that bring together women, clinical and managerial staff may help to shift power dynamics where unconstructive hierarchies exist. Developing shared goals, strategies to achieve them and clarifying roles early on can improve sustainable and effective implementation.

For peer review only - http://bmjopen.bmj.com/site/about/guidelines.xhtml

Conclusions

Quality care for pre-eclampsia requires organisational change to create safe spaces for learning and improvement, alongside efforts to put women at the centre of care and ensure providers are equipped with knowledge, resources and support to adhere to evidence-based practice.

National guidelines for HDP including pre-eclampsia should recommend treatment of BP consistently ≥140/90mmHg to reduce the risk of developing severe hypertension and other complications, and to urgently treat severe hypertension ≥160/110mmHg.[7] They should also include guidance for professionals on counselling women about their future risks, including of having a subsequent pregnancy affected by pre-eclampsia, cardiovascular disease and stroke, and strategies to reduce these. As the growing burden of non-communicable disease emerges globally, opportunities should be seized whilst women are engaged with health services to optimise longer-term outcomes.

For peer review only

More research is required to understand whether evidence established in high resource settings is applicable in LMICs. This should include implementation research and cost-effectiveness of early prediction and prevention strategies.[29]

It is acknowledged that staff are working under difficult conditions within resource constrained systems. This study allows a focus for where to instigate system change to improve the management of pre-eclampsia for women and support staff to deliver quality care.

Acknowledgements

We gratefully thank all participants for their time and contributions to the study. We wish to thank Sewit Hessebon, Hanna Negussie and Medhanit Getachew.

CH receives support from AMARI as part of the DELTAS Africa Initiative [DEL-15-01. JS is an NIHR Senior Investigator and is supported by the National Institute for Health Research (NIHR) Applied Research Collaboration South London (NIHR ARC South London) at King's College Hospital NHS Foundation Trust.

Competing interests: none

For peer review only

Authorship contributions

TR: Conceptualisation, Methodology, Investigation, Data curation, Formal analysis, Visualisation, Writing - original draft, Writing - reviewing & editing

AHS: Conceptualisation, Funding acquisition, Supervision, Writing - reviewing & editing

JS: Conceptualisation, Funding acquisition, Methodology, Supervision, Writing - reviewing &editing

TEG: Investigation, Formal analysis, Writing - reviewing & editing

RDG: Investigation, Formal analysis, Writing - reviewing & editing

AA: Conceptualisation, Funding acquisition, Writing - reviewing & editing

RM: Conceptualisation, Methodology, Funding acquisition, Supervision, Writing - reviewing& editing

CH: Conceptualisation, Methodology, Funding acquisition, Supervision, Writing - reviewing& editing

Availability of data and materials

For peer review only

The data generated and analysed during this study are not publicly available to protect individual anonymity but may be available from the corresponding author on reasonable request.

Funding

This research was funded by the National Institute of Health Research (NIHR) Global Health Research Unit on Health System Strengthening in sub-Saharan Africa, King's College London (GHRU 16/136/54) and CH through an NIHR RIGHT grant (NIHR200842) using UK aid from the UK Government. The views expressed are those of the authors and not necessarily those of the NHS, the NIHR or the Department of Health and Social Care.

For peer review only

References

1. Say L, Chou D, Gemmill A, et al. Global causes of maternal death: a WHO systematic analysis. *Lancet Glob Health* 2014;2(6):e323-33. doi: 10.1016/S2214-109X(14)70227-X [published Online First: 2014/08/12]

2. Mersha AG, Abegaz TM, Seid MA. Maternal and perinatal outcomes of hypertensive disorders of pregnancy in Ethiopia: systematic review and meta-analysis. *BMC*

Pregnancy Childbirth 2019;19(1):458. doi: 10.1186/s12884-019-2617-8 [published Online First: 2019/12/05]

3. Abalos E, Cuesta C, Grosso AL, et al. Global and regional estimates of preeclampsia and eclampsia: a systematic review. *Eur J Obstet Gynecol Reprod Biol* 2013;170(1):1-7. doi: 10.1016/j.ejogrb.2013.05.005 [published Online First: 2013/06/12]

4. Abalos E, Cuesta C, Carroli G, et al. Pre-eclampsia, eclampsia and adverse maternal and perinatal outcomes: a secondary analysis of the World Health Organization Multicountry Survey on Maternal and Newborn Health. *BJOG* 2014;121 Suppl 1:14-24. doi: 10.1111/1471-0528.12629 [published Online First: 2014/03/20]

5. Berhan Y, Berhan A. Causes of maternal mortality in Ethiopia: a significant decline in abortion related death. *Ethiop J Health Sci* 2014;24 Suppl:15-28. doi: 10.4314/ejhs.v24i0.3s [published Online First: 2014/12/10]

6. Knight M NM, Tuffnell D, Kenyon S, Shakespeare J, Brocklehurst P, Kurinczuk JJ (Eds.) on behalf of MBRRACE-UK. . Saving Lives, Improving Mothers' Care - Surveillance of maternal deaths in the UK 2012-14 and lesson learned to inform maternity care for the UK and Ireland Confidential Enquiries into Maternal Deaths and Morbidity 2009-14.: Oxford: National Perinatal Epidemiology Unity, University of Oxford 2016, 2016.

For peer review only

7. Brown MA, Magee LA, Kenny LC, et al. Hypertensive Disorders of Pregnancy: ISSHP Classification, Diagnosis, and Management Recommendations for International Practice. *Hypertension* 2018;72(1):24-43. doi: 10.1161/HYPERTENSIONAHA.117.10803 [published Online First: 2018/06/15]

8. NICE. Hypertension in pregnancy: diagnosis and management: National Institute for Health and Care Excellence; 2019 [Available from: https://www.nice.org.uk/guidance/ng133.

9. Rolnik DL, Wright D, Poon LC, et al. Aspirin versus Placebo in Pregnancies at High Risk for Preterm Preeclampsia. *N Engl J Med* 2017;377(7):613-22. doi: 10.1056/NEJMoa1704559 [published Online First: 2017/06/29]

10. Hofmeyr GJ, Lawrie TA, Atallah AN, et al. Calcium supplementation during pregnancy for preventing hypertensive disorders and related problems. *Cochrane Database Syst Rev* 2018;10:CD001059. doi: 10.1002/14651858.CD001059.pub5 [published Online First: 2018/10/03]

11. Magee LA, von Dadelszen P, Rey E, et al. Less-tight versus tight control of hypertension in pregnancy. *N Engl J Med* 2015;372(5):407-17. doi: 10.1056/NEJMoa1404595 [published Online First: 2015/01/30]

For peer review only

12. Abalos E, Duley L, Steyn DW, et al. Antihypertensive drug therapy for mild to moderate hypertension during pregnancy. *Cochrane Database Syst Rev* 2018;10:CD002252. doi: 10.1002/14651858.CD002252.pub4 [published Online First: 2018/10/03]

13. Which anticonvulsant for women with eclampsia? Evidence from the Collaborative Eclampsia Trial. *Lancet* 1995;345(8963):1455-63. [published Online First: 1995/06/10]

14. Duley L, Gulmezoglu AM, Henderson-Smart DJ, et al. Magnesium sulphate and other anticonvulsants for women with pre-eclampsia. *Cochrane Database Syst Rev* 2010(11):CD000025. doi: 10.1002/14651858.CD000025.pub2 [published Online First: 2010/11/12]

15. Roder-DeWan S, Nimako K, Twum-Danso NAY, et al. Health system redesign for maternal and newborn survival: rethinking care models to close the global equity gap. *BMJ Glob Health* 2020;5(10) doi: 10.1136/bmjgh-2020-002539 [published Online First: 2020/10/16]

16. Hanson C, Waiswa P, Pembe A, et al. Health system redesign for equity in maternal and newborn health must be codesigned, country led, adapted to context and fit for purpose. *BMJ Glob Health* 2020;5(10) doi: 10.1136/bmjgh-2020-003748 [published Online First: 2020/10/16]

For peer review only

For peer review only - http://bmjopen.bmj.com/site/about/guidelines.xhtml

17. Kruk ME, Gage AD, Arsenault C, et al. High-quality health systems in the Sustainable Development Goals era: time for a revolution. *Lancet Glob Health* 2018;6(11):e1196-e252. doi: 10.1016/S2214-109X(18)30386-3 [published Online First: 2018/09/10]

18. Vousden N, Lawley E, Seed PT, et al. Incidence of eclampsia and related complications across 10 low- and middle-resource geographical regions: Secondary analysis of a cluster randomised controlled trial. *PLoS Med* 2019;16(3):e1002775. doi: 10.1371/journal.pmed.1002775 [published Online First: 2019/03/30]

19. WHO U, UNFPA, World Bank Group and the United Nations Population Division. Maternal mortality in 2000-2017: Internationally comparable MMR estimates by the Maternal Mortality Estimation Inter-Agency Group (MMEIG); 2019 [Available from: https://www.who.int/gho/maternal_health/countries/eth.pdf.

20. Ethiopia FMoH. Health Sector Transformation Plan II. https://www.familyplanning2020.org/sites/default/files/HSTP-II.pdf, 2021.

21. Vindrola-Padros C, Vindrola-Padros B. Quick and dirty? A systematic review of the use of rapid ethnographies in healthcare organisation and delivery. *BMJ Qual Saf* 2018;27(4):321-30. doi: 10.1136/bmjqs-2017-007226 [published Online First: 2017/12/22]

For peer review only

22. Braun V, Clarke V. Using thematic analysis in pyschology. *Qualitative Research in Pyschology* 2006;3:2, 77-101 doi: 10.1191/1478088706qp063oa

23. Dixon-Woods M. What can ethnography do for quality and safety in health care? *Qual Saf Health Care* 2003;12(5):326-7. doi: 10.1136/qhc.12.5.326 [published Online First: 2003/10/09]

24. Cupit C, Mackintosh N, Armstrong N. Using ethnography to study improving healthcare: reflections on the 'ethnographic' label. *BMJ Qual Saf* 2018;27(4):258-60. doi: 10.1136/bmjqs-2017-007599 [published Online First: 2018/02/22]

25. WHO. International Classification of Diseases (ICD-11) [Available from: https://www.who.int/standards/classifications/classification-of-diseases2019.

26. Scott G, Gillon TE, Pels A, et al. Guidelines-similarities and dissimilarities: a systematic review of international clinical practice guidelines for pregnancy hypertension. *Am J Obstet Gynecol* 2020 doi: 10.1016/j.ajog.2020.08.018 [published Online First: 2020/08/24]

27. Tesfaye B, Sinclair K, Wuehler SE, et al. Applying international guidelines for calcium supplementation to prevent pre-eclampsia: simulation of recommended dosages suggests risk of excess intake in Ethiopia. *Public Health Nutr* 2018:1-11. doi: 10.1017/S1368980018002562 [published Online First: 2018/10/16]

For peer review only

For peer review only - http://bmjopen.bmj.com/site/about/guidelines.xhtml

28. WHO recommendation: calcium supplementation during pregnancy for the prevention of pre-eclampsia and its complications. Geneva: World Health Organisation, 2018.

29. Poon LC, Shennan A, Hyett JA, et al. The International Federation of Gynecology and Obstetrics (FIGO) initiative on pre-eclampsia: A pragmatic guide for first-trimester screening and prevention. *Int J Gynaecol Obstet* 2019;145 Suppl 1:1-33. doi: 10.1002/ijgo.12802 [published Online First: 2019/05/22]

30. Gupta M, Shennan AH, Halligan A, et al. Accuracy of oscillometric blood pressure monitoring in pregnancy and pre-eclampsia. *Br J Obstet Gynaecol* 1997;104(3):350-5. doi: 10.1111/j.1471-0528.1997.tb11467.x [published Online First: 1997/03/01]

31. Nathan HL, de Greeff A, Hezelgrave NL, et al. An accurate semiautomated oscillometric blood pressure device for use in pregnancy (including pre-eclampsia) in a low-income and middle-income country population: the Microlife 3AS1-2. *Blood Press Monit* 2015;20(1):52-5. doi: 10.1097/MBP.0000000000000086 [published Online First: 2014/09/23]

32. Nathan HL, Vousden N, Lawley E, et al. Development and evaluation of a novel Vital Signs Alert device for use in pregnancy in low-resource settings. *BMJ Innov* 2018;4(4):192-98. doi: 10.1136/bmjinnov-2017-000235 [published Online First: 2018/10/16]

For peer review only

For peer review only - http://bmjopen.bmj.com/site/about/guidelines.xhtml

33. Nathan HL, Boene H, Munguambe K, et al. The CRADLE vital signs alert: qualitative evaluation of a novel device designed for use in pregnancy by healthcare workers in low-resource settings. *Reprod Health* 2018;15(1):5. doi: 10.1186/s12978-017-0450-y [published Online First: 2018/01/07]

34. Payne BA, Hutcheon JA, Ansermino JM, et al. A risk prediction model for the assessment and triage of women with hypertensive disorders of pregnancy in low-resourced settings: the miniPIERS (Pre-eclampsia Integrated Estimate of RiSk) multi-country prospective cohort study. *PLoS Med* 2014;11(1):e1001589. doi: 10.1371/journal.pmed.1001589 [published Online First: 2014/01/28]

35. Ansari N, Manalai P, Maruf F, et al. Quality of care in early detection and management of pre-eclampsia/eclampsia in health facilities in Afghanistan. *BMC Pregnancy Childbirth* 2019;19(1):36. doi: 10.1186/s12884-018-2143-0 [published Online First: 2019/01/20]

36. Aveling EL, Zegeye DT, Silverman M. Obstacles to implementation of an intervention to improve surgical services in an Ethiopian hospital: a qualitative study of an international health partnership project. *BMC Health Serv Res* 2016;16(1):393. doi: 10.1186/s12913-016-1639-4 [published Online First: 2016/08/18]

For peer review only

37. Canavan ME, Brault MA, Tatek D, et al. Maternal and neonatal services in Ethiopia: measuring and improving quality. *Bull World Health Organ* 2017;95(6):473-77. doi: 10.2471/BLT.16.178806 [published Online First: 2017/06/13]

Figure Legend:

Figure 1. Ethiopian Health System Structure

For peer review only

Figure 1. Ethiopian Health System Structure [20]

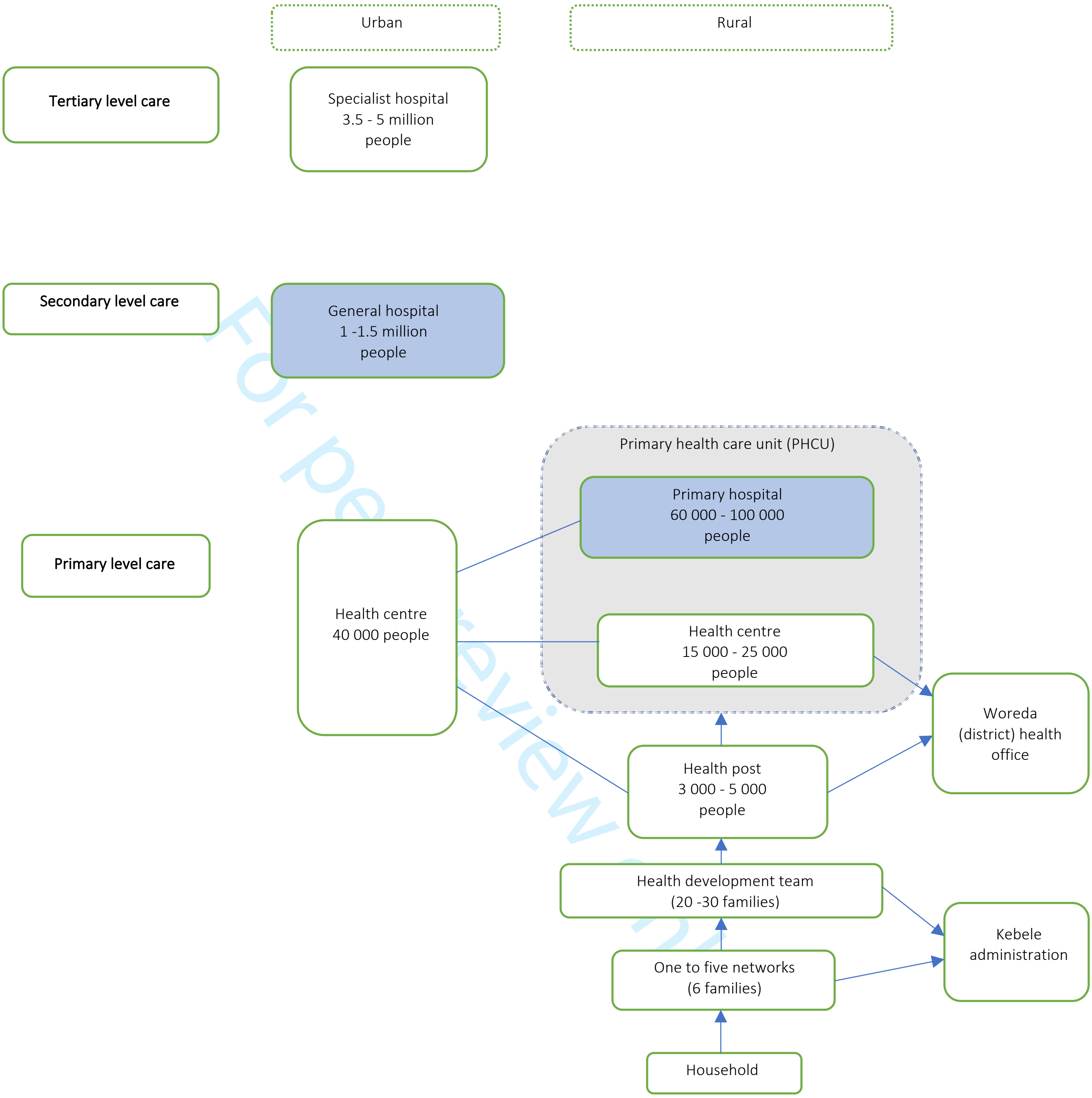

Author reflexivity statement

How does this study address local research and policy priorities?

This study examines hospital level maternity care in rural Ethiopia focusing on pre-eclampsia. This study was nested within a wider project focussing on health system strengthening. The Ethiopian Federal Ministry of Health produces five-yearly strategic plans aiming to drive improvements in the health sector. The Health Sector Transformation Plan for the year 2015/16 to 2019/2020 focussed on quality, equity, respectful and person-centred healthcare. Our study identified numerous systemic barriers to the provision of quality, person-centred care for pre-eclampsia in hospitals.

How were local researcher involved in the study design?

ASSET (Health System Strengthening in sub-Saharan Africa) in Ethiopia builds on a longstanding collaboration between Addis Ababa University and King's College London. The ASSET team within Ethiopia included the Co-Principal Investigator for the maternal healthcare platform (AA), who had input into the study design and protocol development, as well as local research coordinators (TEG, SH, HN and MG) who were involved in data collection and analysis. Additionally, there was a local clinical researcher (RDG) who was involved in the ethnographic observational data collection, data analysis and manuscript reviewing and editing. The overall Principal Investigator (CH) is a UK researcher, living and working long-term in Ethiopia, with extensive experience of organising international research collaborations.

How has funding been used to support the local research team?

This study has been used to support a number of full-time local research staff. The wider ASSET project has supported capacity building work including one PhD candidate and one post-doctoral fellow in Ethiopia.

How are research staff who conducted data collection acknowledged?

The research staff who conducted data collection are either included as authors (TR, TEG and RDG) where they also contributed to data analysis and manuscript development, or are included in the Acknowledgements section (SH, HN and MG).

Do all members of the research partnership have access to the study data?

All members of the partnership have access to data.

For peer review only

How was the data used to develop analytical skills within the partnership?

Team members attended qualitative research workshops facilitated by senior academics in the authorship team. During the participant observation data collection, early career researchers were encouraged to practice reflexivity and were supported to do this by senior researchers, via online meetings and discussions. The analysis for this study was iterative and involved close collaboration between partners based in the UK and Ethiopia.

How have research partners collaborated in interpreting study data?

Research partners collaborated closely in interpreting this study data including contemporaneous discussion and reflection during ethnographic participant observation. The field notes were discussed, amalgamated and later, along with interview transcripts, were coded by TR and TEG. RH and CH collaborated on the development of themes and relationships between them.

How were the research partners supported to develop writing skills?

As part of the ASSET project capacity building work, writing workshops were held in Ethiopia to support early career researchers to develop and refine their writing skills

How will the research products be shared to address local needs?

We aim to publish this study with open access. We also held meetings with local hospital staff to feed back our findings from the participant observations and this has helped to inform ongoing quality improvement within facilities. Findings have also been fed back to policy makers and planners within Ethiopia.

How is the leadership, contribution and ownership of this work by LMIC researchers recognised within the authorship?

We acknowledge the authorship team is predominantly based in high-income countries. The lead author (TR) is a PhD candidate based in London and this study forms part of her thesis. The last author (CH) is based in Ethiopia. We acknowledge the invaluable contributions from our Ethiopian team (TEG, RH, AA).

How have early career researchers across the partnership been included within the authorship team?

There are three early career researchers within the authorship team from both low- and high-income countries. The lead author is an early career researcher.

For peer review only

How has gender balance been addressed within the authorship?

Six authors are female, and two authors are male.

How has the project contributed to training LMIC researchers?

The authorship is composed a mix of senior and early career researchers. The local research team received training on qualitative research methods including conducting ethnography and attended workshops on data analysis and manuscript writing. The ASSET project has supported one PhD fellow and one post-doctoral fellow from Ethiopia.

How has the project contributed to improvements in the local infrastructure?

The sub-study reported in this paper has not directly contributed to improvements in local infrastructure but has highlighted some of the organisational and systemic barriers to the provision of quality care including a lack of consistent access to resources and support. The diagnostic phase of the ASSET project aimed to understand health system bottlenecks to improving quality maternal care, of which this study formed a part. The wider ASSET project is implementing health strengthening interventions including clinical communication skills training, quality improvement and introducing a low cost, accurate and validated blood pressure and heart rate monitor.

What safeguarding procedures were used to protect local study participants and researchers?

All methods were carried out in accordance with relevant guidelines and regulations including the Declaration of Helsinki. The ethnographic observational data was collected by pairs of researchers always including a clinician and Amharic speaker. Ethical challenges about when and how to intervene when questionable practices were observed, were discussed between clinical members of the research team. Clinical researchers assisted in life-threatening situations.

For peer review only

Version 2_Paper 3_Ethnographic perspectives_ASSET

Nodes\\Synthesis 1_TR_TE_combined\\Synthesis 2_themes_V1.0_2\\Synthesis 3

Name	Description	Files	References
1.0 Inconsistencies and inequity_Analysis 1	Inconsistency in how the 'rules' are applied may exacerbate inequities but may also endeavour to ameliorate them	0	0
Clinical factors		0	0
Clinical competence	examples of factors affecting ability to provide safe care. Relates to ability to accurately assess and manage patients. May relate to a lack of awareness, knowledge, training or supervision.	25	48
Clinical uncertainty	Explicit or implicit uncertainty or variance in practice. May relate to decision-making, processes of care, deviance from recommendations in guidelines, complexity in clinical case.	25	43
Recognition of risk or problems	Issues relating to identification of increased risk of complications or need for surveillance/action. Ability to perceive problems.	31	89
Theoretical processes of care_pre-eclampsia	Pathways or processes for how patients with high blood pressure or pre-eclampsia move between levels within or between health facilities, in theory (as opposed to what actually happens/is observed).	11	22
Quality issues		0	0
Adherence to guidelines	Examples of how care relates to national or local guidelines	22	55
Communication between staff	How, when and why staff communicate with each other	23	66
Communication with patients and family	Any communications or verbal interactions with patients or their families, either directly observed or described. The content of, why, when or how, communication with patients and their attendants.	24	89
Conflict between staff	Includes disagreements, differences in opinions or different approaches to problems or situations	10	15
Conscientiousness	Healthcare professionals being diligent and willing to work efficiently	22	41
Consent	Permission to medical treatments, examinations or procedures. May include examples of	12	18

For peer review only - http://bmjopen.bmj.com/site/about/guidelines.xhtml

Name	Description	Files	References
	consent taken/given or not taken/given. May be verbal or written or sometimes implied e.g holding out arm for a blood pressure check.		
Detection or surveillance_pre-eclampsia	Any observations relating to detection of pre-eclampsia including BP monitoring, screening urine or blood tests for end organ damage.	22	54
Quality of documentation	use of written records to note down care given including monitoring (checking vital signs), investigations, treatments, diagnoses, patient details.	27	98
Timely management_pre-eclampsia	Observations or descriptions of process or sequence of care relating to pre-eclampsia, factors relating to reducing or contributing to delays in care for pre-eclampsia or management of high blood pressure.	6	13
Transparency and accountability	Observations or descriptions relating to (un)ethical professional practice, recognition of errors, appropriate action taken. Relates to open communication and good governance. Transparency relates to ensuring information is available to patients and management, to protect patient safety. Accountability to ensure providers/facilities are held responsible for actions or consequences of errors.	19	50
Structural or systems barriers and facilitators		0	0
Access to medications	Issues relating to availability or lack of medications within hospital pharmacy. Relates to organisation with pharmacy, procurement, supply chains and financing.	9	21
Availability and awareness of guidelines	relates to providers/healthcare workers awareness of relevant clinical guidelines and their availability in clinical areas for use	13	26
Availability and utilisation of WASH	Water And Sanitation Hygiene. Availability, access and utilisation of water and sanitation equipment including soap.	14	22
Cleanliness	General cleanliness of rooms and facility. May include factors relating to patient/family behaviour such as disposal of general waste. And/or behaviours or practices of clinical or cleaning staff.	14	30
Electricity supply	Lack of access or supply of electricity	6	13
Inconsistency in directives or care	Failure to standardised procedures. May be due to changes in advice from management, differences in understanding or interpretation of guidance or rules or unclear guidance.	16	27
Organisation of services	Where distinct services are offered and which cadres of staff cover these services.	15	26
Payment for maternal care services	Details of payments required or made by patients or their families for services not provided for	16	33

For peer review only - http://bmjopen.bmj.com/site/about/guidelines.xhtml

Name	Description	Files	References
	free at the point of care. Out of pocket payments.		
Staffing	Details of numbers, cadres of staff providing care, availability out of hours and access to specialists outside daytime hours or on weekends. Includes issues related to understaffing/lack of required staff numbers for safe or effective practice.	23	62
Support and training for staff	References to professional development or learning and support or need for any of these.	16	57
Triage process and initial management	Any observations or descriptions relating to when patient's first arrive in hospital, initial assessment and prioritisation of patients/conditions. Who does this, where, how. Included details of initial place of care, cadres of health work providing care, initial investigations, assessment and any treatments given immediately.	16	48
Workload	Factors influencing the amount of work and productivity of the facility. Related to utilisation of services and workforce structures and numbers.	22	67
2.0 Improvisation_Analysis 1	Making do or tinkering in attempt to mitigate systemic constraints	0	0
Appropriateness of referrals	Factors relating to whether referrals are timely/late AND/OR appropriate or inappropriate in terms of clinical need/level of care at receiving facility AND/OR appropriate communication to receiving hospital in advance	10	16
Availability of clinical investigations	included any lab tests, ultrasound, etc.	12	18
Availability of equipment and materials	Availability of materials and essential equipment e.g gloves, sheets, resuscitation equipment. EXCLUDES medication	28	85
Blood transfusion	Issues relating to availability, access to, planning, supply of blood and blood products including plasma/FFP.	12	29
Clinical decision-making	How, when and where clinicians make decisions about management of patients. e.g whether require admission, involving colleagues/team decision-making	26	53
Decision re mode of delivery	Factors influencing how decisions are made about how to deliver babies e.g Caesarean delivery versus vaginal delivery. Also includes spontaneous vaginal delivery (SVD) versus instrumental birth (ventouse or forceps delivery).	8	12
Decision re timing of delivery	Factors influencing when clinicians may recommend birth, if women present with problems or require intervention.	11	22
Efficiency	How resources, time or people are used to reduce waste. Related to provision of quality care.	13	20
Escalation of care	Processes of referral on to other clinicians or higher level facility, where more skills, knowledge	31	89

For peer review only - http://bmjopen.bmj.com/site/about/guidelines.xhtml

Name	Description	Files	References
	or resources are required in making a clinical assessment or giving treatment.		
Family support	Support from partners or wider family, may be emotional or practical support. Relates to enabling access and provision of appropriate, quality care in terms of patient centred care, timely care if support from family etc.	18	45
Fetal or baby care and monitoring	included observed processes of care or descriptions of how fetal monitoring is carried out including assessment of heart rate or obstetric ultrasound	13	42
Observed processes of care_pre-eclampsia or severe hypertension	Includes observed processes of care for women with severe hypertension (160/110mmHg). Examination, ordering investigations, prescribing medication, admission criteria.	16	36
Observed processes of care_magnesium sulphate	More detailed accounts of magnesium sulphate administration or use including dosing, length of treatment, reasons for initiation or discontinuing.	10	15
Reassessment	rechecking vital signs, clinical condition or investigations. Relates to timely detection of problems, detection of worsening problems, need to escalate and provision of quality care.	18	36
Physical organisation of care	Physical locations within the hospital, designated to particular services and how these are organised.	18	41
Patient flow	the movement of patients through the facility/different stages of care from triage/admission to discharge	5	16
Precarity	Uncertainty or insecurity in care given, may be due to miscommunication or lack of adherence to recommended/evidence-based care.	21	34
Prioritisation	Processes of deciding on relative importance of tasks or problems.	9	19
Privacy	Issues relating to provision of respectful care, including offering to cover patients during examination, providing space for using toilet facilities/bed pan in a space without others around.	18	57
Roles and responsibilities	Descriptions or observations relating to professional roles and responsibilities including particular tasks and duties undertaken.	23	74
Safety	Any issues relating to patient or staff safety including structural issues or processes of care. Includes observations or descriptions of unsafe practice relating to administration of drugs, use of medical devices, unsafe injection practices, hygiene and infection control. Includes appropriate disposal of clinical waste.	20	47
Theoretical processes of care_general	Pathways or processes for how patients move between levels within or between health facilities, in theory (as opposed to what actually happens/is observed). RELATING TO ANY CONDITION	11	17

For peer review only - http://bmjopen.bmj.com/site/about/guidelines.xhtml

Name	Description	Files	References
3.0 Trust and support_Analysis 1		0	0
Acceptance of treatment or advice	Discussion or views about whether patients and/or families accept treatments or management as advised by caregivers. May involve decisional conflict.	20	49
Authority structures	Interactions or examples of hierarchy within the care system. Included both health professionals e.g midwives, nurses, IESO, obstetrician, medical director and auxiliary staff e.g cleaners, lab technicians, and also decision-makers e.g women, partners	16	24
Availability of specialist staff	Access and availability. Includes staff with any specialist skills e.g doctors (obstetrician/gynaecologist), laboratory staff	16	33
Barriers and facilitators to follow up	Challenges that hinder mothers attending for follow up either routine or if notice danger signs/have a problem AND/OR factors that can improve attendance at facility, acceptance of advice or treatments	14	36
Communication with patients and family	Any communications or verbal interactions with patients or their families, either directly observed or described. The content of, why, when or how, communication with patients and their attendants.	24	89
Creating community awareness	Health promotion activities to improve knowledge and awareness of danger signs during pregnancy, birth and postnatally.	14	43
Defensiveness	Behaviour observed or described relating to being anxious or to avoid potential criticism.	10	18
Feedback to referring health centres	System or processes to allow information to go back to referring facilities including clinical outcomes, appropriateness of referrals, appropriateness of initial triage/management, documentation, staff accompanying on referral	7	15
Inertia	Tendency to do nothing/not take action when required despite lack of barriers.	13	25
Maintenance of equipment	includes charging equipment, replacement of batteries or broken parts, servicing equipment etc.	10	18
Neglect	Failure to provide quality care or abandonment of patient needs	13	20
Patient concerns	Any issues relating to anxieties or problems expressed by patients or described by those caring for them. Relates to access and quality of care.	23	60
Professional satisfaction	Health workers feelings about their roles, how they are valued, thoughts about pay/compensation for work and incentives	7	16
Self-appraisal of skills	Assessment by individuals of themselves/their own skills/ability or of the facilities within which they work. Self-reflection.	8	19

For peer review only - http://bmjopen.bmj.com/site/about/guidelines.xhtml

Name	Description	Files	References
Team working	Collaboration between professionals to perform duties including examples of joint decision making (included examples of good or poor team working)	11	26
Trust in resources	Relating to confidence in either physical/material or human resources.	15	18
Undermining	Observations or descriptions of behaviour that diminishes power of an individual to act/carry out their responsibilities.	7	7
Use of traditional medicines	Any issues relating to pregnant women using traditional medicines and how this relates to their care in health facilities.	7	13
Women's experience of care or services	includes their perceptions about quality of care in hospital, continuity of carer, information provided to them, how professional communicated, if they were counselled appropriately (general impressions that may not fit into other codes)	7	25

For peer review only

For peer review only - http://bmjopen.bmj.com/site/about/guidelines.xhtml

Consolidated criteria for reporting qualitative studies (COREQ) checklist

**Number**	**Item**	**Ethiopia**	**Location in manuscript (section, page number)**
**Domain 1: Research team and reflexivity**			
1.	Interviewer/facilitator	RD, TR, TE	Methods, data collection, pg5
2.	Credentials		
3.	Occupation	Ethiopian obstetrician, British obstetrician, Social epidemiology research coordinator	
4.	Gender	F/F/F	
5.	Experience and training	Prior experience of qualitative data collection	
6.	Relationship established	No	
7.	Participant knowledge of the interviewer	No	
8.	Interviewer characteristics	Involved in wider health system strengthening project	
**Domain 2: study design**			

For peer review only

9.	Methodological orientation and theoretical frameworks	Thematic analysis using an inductive approach	Methods, data analysis, pg6
10.	Sampling	Purposive sampling	
11.	Method of approach	Face to face	
12.	Sample size	21 IDI	
13.	Non-participations	0	
14.	Setting of data collection	Primary and general hospital	
15.	Presence of non-participants	No	
16.	Description of sample	Yes	
17.	Topic guide	Example guide included as supplementary file.	
18.	Repeat interviews	No, further sampling where data saturation needed e.g women with previous pre-eclampsia	

For peer review only

19.	Audio recorded	Yes	
20.	Field notes	Yes	
21.	Duration of interviews/FGD	Variable	
22.	Data saturation	Yes	
23.	Transcripts returned for participant checking	No, but member checking via Theory of Change workshops for later stage of wider projects	
**Domain 3: analysis and findings**			
24.	Number of data coders	2	
25.	Description of coding tree	Yes - provided in supplementary file	
26.	Derivation of themes	Derived from data iteratively	
27.	Software	NVivo 12	
28.	Participant checking	Yes. See 23.	

For peer review only

29.	Quotations presented	Yes, participant ID provided	
30.	Data findings consistent	Yes	
31.	Clarity of major themes	Yes, quotations to support in Table 1 & 3	Results, pg 7
32.	Clarity of minor themes	Yes, see coding structure in supplementary file	

For peer review only

BMJ Open

Understanding challenges as they impact on hospital level care for pre-eclampsia in rural Ethiopia: a qualitative study

Journal:	*BMJ Open*
Manuscript ID	bmjopen-2022-061500.R1
Article Type:	Original research
Date Submitted by the Author:	17-Aug-2022
Complete List of Authors:	Robbins, Tanya; King's College London, Department of Women and Children's Health<br>Shennan, Andrew; King's College London, Department of Women and Children’s Health, School of Life Course Sciences<br>Sandall, Jane; King's College London, Department of Woman and Children's Health<br>Eshetu Guangul , Tigist; Addis Ababa University College of Health Sciences, Centre for Innovative Drug Development and Therapeutic Trials for Africa (CDT-Africa)<br>Demissew, Rahel; Addis Ababa University, Department of Obstetrics and Gynecology<br>Abdella, Ahmed; Addis Ababa University College of Health Sciences, Department of Obstetrics & Gynaecology<br>Mayston, Rosie; King's College London, Department of Global Health and Social Medicine<br>Hanlon, Charlotte; King's College London, Institute of Psychiatry, Psychology and Neuroscience, Health Service and Population Research Department, Centre for Global Mental Health; Addis Ababa University, Centre for Innovative Drug Development and Therapeutic Trials for Africa (CDT-Africa), College of Health Sciences
**Primary Subject Heading**:	Obstetrics and gynaecology
Secondary Subject Heading:	Global health, Health policy, Qualitative research, Patient-centred medicine
Keywords:	OBSTETRICS, Hypertension < CARDIOLOGY, QUALITATIVE RESEARCH, Change management < HEALTH SERVICES ADMINISTRATION & MANAGEMENT, Health policy < HEALTH SERVICES ADMINISTRATION & MANAGEMENT, Organisation of health services < HEALTH SERVICES ADMINISTRATION & MANAGEMENT

SCHOLARONE™
Manuscripts

I, the Submitting Author has the right to grant and does grant on behalf of all authors of the Work (as defined in the below author licence), an exclusive licence and/or a non-exclusive licence for contributions from authors who are: i) UK Crown employees; ii) where BMJ has agreed a CC-BY licence shall apply, and/or iii) in accordance with the terms applicable for US Federal Government officers or employees acting as part of their official duties; on a worldwide, perpetual, irrevocable, royalty-free basis to BMJ Publishing Group Ltd ("BMJ") its licensees and where the relevant Journal is co-owned by BMJ to the co-owners of the Journal, to publish the Work in this journal and any other BMJ products and to exploit all rights, as set out in our licence.

The Submitting Author accepts and understands that any supply made under these terms is made by BMJ to the Submitting Author unless you are acting as an employee on behalf of your employer or a postgraduate student of an affiliated institution which is paying any applicable article publishing charge ("APC") for Open Access articles. Where the Submitting Author wishes to make the Work available on an Open Access basis (and intends to pay the relevant APC), the terms of reuse of such Open Access shall be governed by a Creative Commons licence – details of these licences and which Creative Commons licence will apply to this Work are set out in our licence referred to above.

Other than as permitted in any relevant BMJ Author's Self Archiving Policies, I confirm this Work has not been accepted for publication elsewhere, is not being considered for publication elsewhere and does not duplicate material already published. I confirm all authors consent to publication of this Work and authorise the granting of this licence.

For peer review only

Title

Understanding challenges as they impact on hospital level care for pre-eclampsia in rural Ethiopia: a qualitative study

Corresponding author details

Tanya Robbins
Department of Women and Children's Health
School of Life Course Science
King's College London
10th Floor, North Wing
St Thomas' Hospital
Westminster Bridge Road
London SE1 7EH
tanya.robbins@kcl.ac.uk

For peer review only

Authors

Tanya Robbins [a] tanya.robbins@kcl.ac.uk
Andrew H Shennan [a] andrew.shennan@kcl.ac.uk

Jane Sandall [a] jane.sandall@kcl.ac.uk

Tigist Eshetu Guangul [b] tigi.eshetu@yahoo.com

Rahel Demissew [c] raheldemissewgy@gmail.com

Ahmed Abdella [c] ahmedamu@yahoo.com

Rosie Mayston [d] rosie.mayston@kcl.ac.uk

Charlotte Hanlon [e, f] charlotte.hanlon@kcl.ac.uk

Affiliations

[a] Department of Women & Children's Health, School of Life Course Sciences, King's College London, St Thomas' Hospital, London, UK

[b] Centre for Innovative Drug Development and Therapeutic Trials for Africa (CDT-Africa), College of Health Sciences, Addis Ababa University, Addis Ababa, Ethiopia

[c] Department of Obstetrics & Gynaecology, College of Health Sciences, Addis Ababa University, Addis Ababa, Ethiopia

[d] Department of Global Health and Social Medicine, King's College London, London, UK

[e] Centre for Global Mental Health, Health Service and Population Research Department, and WHO Collaborating Centre for Mental Health Research and Training, Institute of Psychiatry, Psychology and Neuroscience, King's College London, London, UK

For peer review only

[f] Department of Psychiatry, WHO Collaborating Centre for Mental Health Research and Capacity-Building, School of Medicine, College of Health Sciences, Addis Ababa University, Addis Ababa, Ethiopia

Word count

4868 words

Abstract

Objective

To explore hospital level care for pre-eclampsia in Ethiopia considering the perspectives of those affected and healthcare providers, in order to understand barriers and facilitators to early detection, care escalation and appropriate management.

Setting

A primary and a general hospital in southern Ethiopia.

Participants

For peer review only

Women with lived experience of pre-eclampsia care in hospital, families of women deceased due to pre-eclampsia, midwives, doctors, integrated emergency surgical officers and healthcare managers.

Results

This study identified numerous systemic barriers to provision of quality, person-centred care for pre-eclampsia in hospitals. Individual staff efforts to respond to maternal emergencies were undermined by a lack of consistency in availability of resources and support. The ways in which policies were applied exacerbated inequities in care. Staff improvised as a means of managing with limited material or human resources and knowledge. Social hierarchies and punitive cultures challenged adequacy of communication with women, documentation of care given and supportive environments for quality improvement.

Conclusions

Quality care for pre-eclampsia requires organisational change to create a safe space for learning and improvement, alongside efforts to offer patient-centred care and ensure providers are equipped with knowledge, resources and support to adhere to evidence-based practice.

For peer review only - http://bmjopen.bmj.com/site/about/guidelines.xhtml

Strengths and limitations of this study

- Observational data, triangulated with in-depth interviews alongside relevant health policy and clinical guidelines, enabled a holistic examination of processes and quality of care and the factors challenging them.
- A rapid ethnographic approach enabled timely feedback of key practical findings from our observational work to staff and district level stakeholders.

For peer review only

For peer review only - http://bmjopen.bmj.com/site/about/guidelines.xhtml

Introduction

Poor quality maternal care in hospitals contributes significantly to avoidable deaths. Pre-eclampsia is a leading cause of maternal and perinatal mortality and morbidity globally.[1] The greatest burden is borne by low- and middle-income countries (LMICs).[2] There is a dearth of robust national data on incidence, particularly in LMICs. It is estimated that 2-5% of pregnancies globally are affected by pre-eclampsia.[2 3] In Ethiopia, 19% of maternal deaths are attributed to hypertensive disorders of pregnancy (HDP) compared to 14% worldwide.[1 4 5] In addition to these uncertain estimates, there is an increasing burden of non-communicable diseases in LMICs. These include chronic hypertension and diabetes which are known to increase the risk of developing pre-eclampsia. Good quality care makes a difference to outcomes; the majority of deaths due to pre-eclampsia are preventable. Less than one in a million women delivering in the UK now dies due to pre-eclampsia, compared to an estimated 783 deaths per million in Ethiopia.[6]

Internationally there is a lack of consensus concerning classification, diagnosis and management of pre-eclampsia.[7] Controversy remains over inpatient versus outpatient management, target blood pressure and timing of delivery for preterm pre-eclampsia.[8] However, there is evidence to support prophylaxis with low-dose aspirin and supplemental

For peer review only

calcium if dietary intake is low, treatment of severe hypertension (≥160/100mmHg) and use of magnesium sulphate for eclampsia or pre-eclampsia with severe features.[9-14] Prediction and prevention of pre-eclampsia is an important goal in reducing mortality and morbidity. However robust health systems and continuity across different levels of the care pathway are needed to support effective implementation of these interventions.

Recent proposals for rethinking models of maternity care to improve quality and equity have called for increased focus on centralised services and shifting all deliveries to or near hospital settings.[15] Justifications included the limitations of obstetric risk stratification, low quality care in primary level settings and challenges of referral if complications do occur. However, to address equity, redesign must be country-led, context specific and co-designed with a focus on listening to women and strengthening human resources.[16] As poor quality care emerges as a greater barrier to reducing mortality than insufficient access to services, understanding challenges in secondary care and the experiences of women cared for in hospital is critical.[17] A large, international prospective dataset showed that 61% (28.1% within a central referral hospital) of eclampsia cases in Ethiopia occurred within facilities, highlighting the need to improve quality of care as well as surveillance, early detection and access to appropriate care.[18]

For peer review only - http://bmjopen.bmj.com/site/about/guidelines.xhtml

The objective of this study was to develop rich contextual understanding of the processes and experiences of hospital level care for pre-eclampsia in rural Ethiopia to inform efforts to improve quality.

Methods

Study setting

This study was conducted in a primary and general hospital in the Gurage Zone, Southern Nations, Nationalities and Peoples' Region (SNNPR). This study setting was selected to include a predominantly rural population, spread across lowland and highland areas. This is to provide generalisability of our findings. Geographical barriers in the area are significant and access to ambulances limited. Ethiopia has an ethnically diverse population of over 112 million, 79% of whom live in rural areas. Government-run health services are structured into three-tiers: primary, secondary and tertiary care. See figure 1. The general hospital at secondary level care serves as a referral center for the primary hospital. In the study setting, maternity services at the primary hospital were provided by midwives, junior doctors referred to as general practitioners (GPs) and integrated emergency surgical officers (IESOs). IESOs

For peer review only

are non-medical clinical staff trained to provide emergency surgical care. In addition to these cadres, the general hospital was also staffed by an obstetrician.

Ethiopia has seen significant reductions in maternal mortality ratios over recent decades from 1030 deaths per 100,000 live births in 2000 to an estimated 401 in 2017.[19] However, institutional delivery rates remain low, estimated at 26% in 2016 and only 43% of pregnant women had four or more antenatal care visits in 2019. The Ethiopian Federal Ministry of Health (FMoH) produces strategies to improve all levels of the health system set out in five-yearly Health Sector Transformation Plans to guide healthcare delivery.[20] These align with current international commitments such as achieving quality, person-centred, respectful and equitable healthcare.

Figure 1. Ethiopian Health System Structure

Data collection

We conducted a qualitative study between January 2019 and January 2020, using unstructured participant observation, semi-structured interviews and document analysis of hospital guidelines and meeting minutes. Our research team included both an Ethiopian

For peer review only

(RD) and a British obstetrician (TR) and an Ethiopian social epidemiology researcher (TE). Two researchers conducted rapid ethnographic observation over 16 days between the primary and general hospital.[21] Observers included a clinician and an Amharic speaker at all times. We observed all aspects of maternity care relevant to pre-eclampsia including spending time on the labour ward, in the emergency department, antenatal clinic, inpatient wards and in the operating theatre. Ethical challenges about when and how to intervene when questionable practices were observed, were discussed between clinical members of the research team. Clinical researchers assisted in life-threatening situations. Where staff explicitly asked for assistance with non-critical cases they were encouraged to continue with routine practice. Informal interviews were conducted with staff to enquire about specific aspects of care or interactions during participant observation where further clarification or context was sought. Contemporaneous field notes were taken and discussed between researchers encouraging reflexive practice. 21 formal in-depth semi-structured interviews were conducted. We purposively sampled 10 staff members (two midwives, three IESOs, four doctors including an obstetrician and a district health manager), eight women with lived experience of pre-eclampsia and three family members of women who had died due to pre-eclampsia. Women affected by pre-eclampsia were identified through routinely collected data. The research team conducting interviews were not known to interviewees. Interviews

For peer review only

173 were conducted in Amharic, audio-recorded, transcribed and translated into English.
174 Transcripts were cross-checked by Ethiopian research co-ordinators for accuracy.
175
176 Findings from the participant observation were fed back to staff via a meeting to inform later
177 stages of a wider project on health system strengthening. Minutes from this meeting along
178 with national guidelines relating to care for pre-eclampsia were also used as data sources to
179 triangulate findings and provide relevant context.
180
181 Clinical care guidelines in use at the time of the study included: FMoH Management Protocol
182 on Selected Obstetric Topics 2010, Standard Treatment Guidelines 2014 (STG) for Primary
183 and General Hospitals and a Comprehensive Emergency Obstetric and Newborn Care
184 Training Manual. The FMoH guidelines advocated for four focused antenatal care (ANC)
185 visits emphasising quality rather than quantity. Usual care for women with low-risk
186 pregnancies was provided at health centres and the other visits conducted by health
187 extension workers (HEWs) at health posts close to women's homes. Pregnant women
188 identified to have risk factors including amongst others, previous pre-eclampsia, chronic
189 hypertension, diabetes or renal disease were, according to national guidelines to be referred
190 to higher level care or offered closer follow up.

For peer review only

Data analysis

We undertook thematic analysis using an inductive approach[22]. Field notes, interview transcripts and the minutes of the meeting were uploaded into NVivo12 software. Data were read and re-read; and initial codes were generated, reviewed and refined by TR, TE and CH (see supplementary file 1). All data were then recoded by TR and themes generated and reviewed with CH and RD. Themes and subthemes were checked against the data to examine relationships between them.

Patient and public involvement

The design of this study focussed on examining the lived experience of women affected by pre-eclampsia and their families. We included formal and informal interviews with women. Our research questions were informed by endeavouring to understand the priorities and experiences of patients. They were not involved in the planning, design or analysis of this study.

Results

For peer review only

Women and healthcare providers described significant systemic barriers to the provision of quality care. Basic structural necessities such a running water, soap and electricity were intermittently unavailable. This was particularly evident at the primary hospital. During four days of observation at the primary hospital there was no running water. There were no sinks in any of the inpatient wards or clinical areas, including the infection prevention room at the primary hospital.

Our analysis revealed contextual challenges that women and professionals faced, within which they sought to receive or provide quality care for pre-eclampsia. We present three main themes: inconsistencies and inequity, improvisation and making do, and trust and support.

Theme I: Inconsistencies and inequity

Key guidelines identified by staff as informing care for pre-eclampsia at hospital level were acknowledged by some professionals as being outdated and were adhered to variably. Women admitted to hospital with pre-eclampsia were often managed according to the national guideline but not consistently. Variability in clinical practice related to incomplete clinical assessments, including not eliciting or documenting significant risk factors such as a

For peer review only

For peer review only - http://bmjopen.bmj.com/site/about/guidelines.xhtml

history of eclampsia and previous stillbirth, and a lack of knowledge, for example, of established thresholds of severe hypertension.

> *"She will be given magnesium sulphate (MgSO4) if her systolic BP is above 180 or diastolic above 110, we will give her hydralazine to drop the elevated BP."*
>
> (Midwife, M11)

Guidelines and practice were ambiguous relating to the management of antenatal women presenting with, or at risk of eclampsia, who were preterm and whose blood pressure (BP) normalised. Staff reported that women were discharged prior to delivery if their BP was controlled with antihypertensives.

> *"Q: She came with eclampsia; under what condition you will decide and discharge her?*
>
> *A: When her BP become normal, and her protein level reduces. It will become normal when we give them antihypertensive. There are many mothers who are discharged."*
>
> (Midwife, M11)

For peer review only

Some medical staff reported they used diazepam to control seizures despite the availability of magnesium sulphate and guidelines stating this as the drug of choice.

Women described concerns about competence and safety. Some were particularly worried about the danger this posed to more vulnerable, less educated women who may be unable to advocate for themselves. A family member of a woman who died described conflicted feelings, both troubled by the care received whilst recognising providers' motivations were honourable.

> *"I have seen a lot there, we or you may read English a little and yes, your parents may read Amharic but how about those women who do not know anything. Yes, mistake a lot there, especially practitioners who provide medicine at resting room after surgery if they are not very careful, I do not know who will make them accountable."*
>
> (Woman with previous pre-eclampsia, M28)

For peer review only - http://bmjopen.bmj.com/site/about/guidelines.xhtml

"In this hospital, they don't cooperate. We are grumbling because of them. We are blaming the hospital for her death…Yes, her body refused to circulate someone else's blood and get swelled….I am not better than the health professionals; they were trying to save her not to kill her…yes…I am thinking this was because I am hurt not because I am better than the health professionals."

(Family of deceased woman, M26)

A limited understanding of the aetiology of pre-eclampsia was reflected in the advice given to women regarding their diagnosis.

"They used to tell me not to drink soft drinks, tea and the like. And that I don't have to use too much salt and need to decrease my fluid intake until the blood pressure goes down."

(Woman with previous pre-eclampsia, M37)

Inconsistencies in triage of pregnant women related to a lack of skills, knowledge or experience of staff. Inappropriate triage was reported to result in a failure to recognise risk, escalate and manage complications appropriately.

For peer review only

"One of the things that needs improvement is developing the health professionals' skill and knowledge. Most of the problems are happening because of lack of knowledge. Most things are happening by not considering as a problem when actually problem has happened to mothers. Therefore, by improving the awareness we can change many things."

(Senior Clinician, M32)

"Sometimes triage might not be done appropriately, or due to limited knowledge and skill gap, the general practitioner might miss some cases. But once the surgeon is involved, he will be fully responsible."

(Medical Director, M49)

Relevant to the variation in clinical practice and competence, were inadequate and inequitable access to training opportunities and professional development as described by providers. Some staff linked this to poor support from the hospital administration, whilst a senior clinician perceived a professional culture lacking in motivation to pursue independent study and continuing professional development.

For peer review only

"Most of the time, there are no trainings. The process of informing new information and participating the health professionals on training is very poor. This is one of the problem of administrators. Sometimes there are various types of training opportunities for their health professionals or the administrators will train the health professionals on areas where they believe there is a gap. They may also forbid the health professionals that was requested to get the training by paper from the high-level officials. This is the main problem of the administrators in our health institutions. The training does not have to be for getting the per diem rather it has to be used to updating the knowledge and to apply what you have got from the training. The way of administrators supporting this is very poor."

(IESO, M48)

"The first thing is when there are such trainings, our society as a whole have a very poor reading habit. People don't sit down and read the guideline."

(Medical Director, M49)

For peer review only

Inconsistencies in how policies were implemented were also observed and reported. This variation may have been intended to ameliorate inequities in the system but also exacerbated them. National level policy for free maternal care was implemented differentially dependant on clinical situation. In this study setting, women admitted to hospital with complications such as pre-eclampsia, requiring inpatient care but not delivery, were required to pay for services. Women attending purely for delivery received free care. Whereas women with potentially life-threatening complications requiring several medications, longer inpatient stays and at higher risk of delivering preterm infants in need of neonatal care, who were able to, were required to pay. However, women from low socio-economic status households could be considered for exemption by the hospital management or could access free care if community-based health insurance had been rolled out in their area. Nonetheless, out-of-pocket payments for antihypertensive drugs were common. Financial barriers were observed to present tangible challenges for women with potentially serious complications related to pregnancy. Clinicians were not always aware of policy or how to apply it in practice.

Case vignette

For peer review only

A recently postpartum woman presented to the emergency department with significant pitting oedema to bilateral lower limbs. She was reviewed by the doctor on-call and laboratory investigations were requested. She attended the lab but was asked for 160 birr (£2.80) which she was unable to pay. She did not have a community-based health insurance card. The GP asked her to go back to her village, to collect the insurance card, then to visit the local health centre and ask for referral back to the hospital for a full assessment. She did not return to the hospital that day.

> *"It is not free whether she is here or discharged. The hospital will fulfil things required for delivery, like gloves and others. The mothers may not buy the medication since they may not have money; there are mothers who don't have any money. Thus, even if we told them to buy it, they might not buy the medication… like antihypertensive, they have to pay…Yes, she has to purchase it. If methyldopa or so on is prescribed to her at ANC, she has to purchase it from her own pocket… It would have been good if it is possible to make laboratory services and medications prescribed for the mothers free as well. They would have come if it is free"*
>
> (Midwife, M11)

For peer review only

For peer review only - http://bmjopen.bmj.com/site/about/guidelines.xhtml

"The problem that we have in this hospital is, sometimes after deciding to admit them, there is some kind of problem on the payment system. For example, if a pregnant woman is close to due date and or going to give birth after some days and is going to be admitted, she is not going to pay. But if she has been admitted not for delivery, she would pay. There are these kinds of unclear rules. If it is free for one person, it should be free for all."

(Senior Clinician, M32)

Theme II: Improvisation and making do

Improvisation emerged as a means of managing or making do with limited resources, both material and human, within both hospitals. This centred around a lack of equipment, medications, staff and support. The barriers and facilitators to early detection, care escalation and appropriate management of pre-eclampsia are described in table 1.

BP monitors were lacking in both hospitals. Staff made-do by sharing, negotiating use between departments or improvising with inadequate equipment such as a plastic training stethoscope used with a sphygmomanometer. At the primary hospital, there was no BP

For peer review only

monitor available in the antenatal clinic. If midwives felt a BP check was needed, the woman was escorted to the ward to have this done.

Midwives at the general hospital made do with one semi-functioning cardiotocograph machine between six beds in the “first stage room” and two delivery beds. Midwives could only assess the current heart rate pattern, or sit with women and observe in real-time, as paper was not available to record. Improvisation with regards to fetal monitoring for high-risk woman was common and related to the lack of resources, but also a lack of knowledge of normal parameters and difficulties interpreting fetal heart rate patterns. Failure to recognise abnormal patterns was observed, which led to delays in taking action, either to expedite delivery or escalate. No fetal monitoring took place in the second stage of labour, regardless of earlier concerns. The reasons for this were difficult to ascertain but this was an established culture of practice. On occasions, the management of suspected fetal distress at the general hospital involved the use of 40% dextrose infusions to the mother. These practices may have been related to a lack of specific guidance for midwives on the management of fetal distress.

For peer review only

Assessment of antenatal women presenting with reduced fetal movements close to term, were inadequate. The fetal heart was auscultated to confirm presence. Women were not offered blood pressure or urine screening, assessment of symphysial fundal height or a growth scan. At the primary hospital no continuous fetal monitoring was available and a diagnosis of presumed fetal distress was made following auscultation of decelerations using a fetoscope.

Precarious practices were observed such as the use of fundal pressure during the second stage of labour when providers lacked confidence, the necessary skills or support to perform assisted delivery.

Families offered frequent support to plug gaps in the system, collecting patient notes, requesting blood tests, clearing buckets of urine and blood on labour ward, procuring drugs and equipment from private pharmacies when not available in labour, and providing support for women in labour when allowed.

Table 1. Barriers and facilitators to early detection, care escalation and appropriate management of pre-eclampsia in hospital

For peer review only

Hospital level care for pre-eclampsia	Barriers	Facilitators
Early detection and recognition of risk	• A lack of continuity of care and linkage between general outpatient department (OPD) clinics where women with chronic hypertension, diabetes or renal disease may be managed and high-risk ANC clinic<br>• Unclear guidelines for midwives, IESOs and doctors regarding outpatient management of women referred from lower levels of care with risk factors for pre-eclampsia<br>• A lack of BP monitors in clinical areas where pregnant women were cared for	• Regular blood pressure monitoring was observed for women admitted with severe hypertension (although not always documented)
Escalation of care	• Staff responsible for triaging pregnant women were not always competent, experienced or supported enough to escalate care appropriately<br>• Poor documentation of clinical history, risk factors, examination and care given challenged communication between staff and different levels of the care pathway	• Referrals from lower levels of care (health centres and primary hospital) were reported to be frequent (although reported by staff as not always necessary)<br>• A clear professional hierarchy was described by staff, indicating they knew who they could refer on to
Appropriate management	• National guidelines were not evidence-based or up to date or were unclear, recommending precarious or potentially unsafe practices<br>- Management of hypertension (explicit advice not to treat non-severe hypertension ≥140/90 to 159/109mmHg, lack of timely management of severe hypertension -advice to wait one hour)	• Magnesium sulphate was available, and doses prescribed according to national and international guidelines<br>• Guidelines recommend delivery within 24 hours of onset of symptoms in “severe pre-eclampsia” and within 12 hours of eclampsia.

For peer review only

	- Timing of delivery (recommend delivery when cervix favourable for “mild pre-eclampsia”, advise avoiding post-term pregnancy but no explicit recommendation for delivery at 37 weeks)<br>• A lack of fluid balance monitoring was observed, and injudicious use of intravenous fluids concurrently with furosemide without a clear indication<br>• Timing of antenatal steroids for fetal lung maturation (given to women with preterm pregnancy and hypertension even when delivery was not planned)<br>• Unclear and inconsistent processes for induction of labour, increasing the risk of caesarean section.<br>• Poor documentation of clinical care led to a lack of transparency and accountability<br>• Minimal counselling regarding future risks including in subsequent pregnancies, increased risk of cardiovascular disease and stroke, and no guideline to support this practice	

Theme III: Trust and support

Communication skills and styles varied between individuals with examples of kind, compassionate interactions, professionals checking women’s understanding and providing reassurance. However, a culture of informed consent and shared decision-making was not widespread. Women were frequently subjected to vaginal examinations in an open ward

For peer review only

without privacy. There were observed examples of abrasive interactions and failures to take women's concerns seriously. Decisions about termination of pregnancy for women with early onset pre-eclampsia were made without explanation to women and were not documented in hospital notes. These interactions were described as "oral reviews". Staff were frequently observed carrying out clinical activities without documenting including performing vaginal examinations, ward rounds and administering drugs. Some staff reported this related to care being given outside prescribed protocols, for example, the timing of examinations during labour. Poor quality documentation was also linked to a punitive culture and fear of being blamed for adverse outcomes. Strategies to improve documentation were in progress including a documentation quality audit.

Allowing women family support during labour was inconsistent and limited by structural constraints such as the lack of privacy and space in the labour ward. However, relating back to the initial theme, inequitable application of the rules left women who were less able to advocate for themselves alone and lacking support, whilst others were allowed family members with them. Staff talked about the challenges they felt affected their ability to provide respectful care including workload and not feeling respected themselves.

For peer review only

There were examples of low staff satisfaction reported by women, affecting quality of care. Staff reported low level of professional support from the hospital administration and a sense of moral duty driving their commitment to work. Suggestions about how to support and retain staff reflected a need to develop more nurturing systems with opportunities for feedback to clinicians, both positive and negative. The theme of trust and support is further illustrated in table 2.

Table 2. Theme III: Trust and support

Theme III: Trust and Support	Verbatim quotes
Women experienced or witnessed lack of respectful care or staff attitudes that presented barriers to quality care	*"But there are also doctors who think they know everything, but they are below doctors. To your surprise, you see it many times, those who insult, who say you did not follow here so go away. But it has no problem. She enters for delivery after signing for the risk, right. She will be responsible for everything that could happen to her. But saying go away while she is about to give birth and because she did not follow there, I have seen a lot."* (Woman with previous pre-eclampsia, M28)
Communication with women affected what they understood about their diagnosis and their trust in professionals and the care received	*"I am still not sure whether I do have or don't have the hypertension at that time. Finally, at the hospital I wasn't so sure whether I have hypertension or not but at the hospital, they said, "It is very much elevated, and she might get into a bad condition if we are waiting more than this". Then they made me sign and performed the surgery and I gave birth. That's all about it. I didn't understand whether they did that purposely to intimidate me or whether I exactly do have the hypertension"*

For peer review only

	(Woman with previous pre-eclampsia, M34)
Doubt and anxiety about their diagnosis led to some women searching for alternative opinions	*"Yes, it was at the hospital. I went three places to check my blood as a matter of fact… they told me to wait and I went to other places in the meantime to get my BP checked… When I checked there, the number was the same. So, I returned back to the hospital because I was told to remain there. I was very nervous."* (Woman with previous pre-eclampsia, M25)
Staff felt challenged to provide compassionate and respectful care	*"Due to the labour pain labouring mothers are aggressive sometimes which makes our job a little bit complicated while giving the service. At this moment it is difficult to practice the CRC [compassionate respectful care] as we hoped to. But you have to minimize your own issue and try to help the mother and her child. We should take care of the child regardless of the situation and the protocol sometimes."* (Head midwife, M33)
Women reported staff were dissatisfied and this affected quality of care and communication	*"I hated many of the women. I am busy and I work here unwillingly [the midwife said]. It is her work, right. She gets paid, right. No one is doing one's job happily. She has to communicate peacefully even if she doesn't like the woman's blood or waste."* (Woman with previous pre-eclampsia, M28)
Staff did not feel supported but were motivated to work by a sense of duty	*"Support is very low. You work for your conscience. There are a lot of problems on the administration side. You just work because of feeling responsible."* (IESO, M47)
Staff reported the need for more feedback as well as support to improve retention	*"Giving recognition for the tasks that they have accomplished well. Appreciating or congratulating on a job well done and give constructive criticism when the work lacks will be great. If not, that person will be compelled to seek other more satisfying work environments."* (IESO, M47)

Discussion

For peer review only

This study found the provision of quality, person-centred care for pre-eclampsia in two Ethiopian hospitals was challenged by numerous systemic barriers. A lack of consistency in availability of resources, support and how policies were applied exacerbated inequities in care. This was particularly evident in our examples where women required admission and investigations for complications during pregnancy or the postnatal period but were unable to pay. Understanding structural drivers of poor-quality maternity care and mistreatment is vital to ameliorating the detrimental impact on future help-seeking.[23 24] There is a need to strengthen education and support for staff to provide respectful, person-centred care as well as addressing resource shortages.[25-27]

Variations in clinical competence, knowledge and experience meant staff providing maternity care were not always able to recognise risks or act on them. Outdated, ambiguous guidelines and inequitable opportunities for professional development exacerbated clinical uncertainty and practice not based on evidence. National guidelines specify against the use of antihypertensives for non-severe hypertension and many women with significant hypertension were not treated. Inadequate documentation did not always reflect clinical risks, drugs administered, or care given. Poor quality documentation was linked to the provision of care divergent from protocol and fear of blame for poor outcomes. A lack of

For peer review only

transparency, accountability and systems for support were observed and reported. Improvisation was used to address shortages in resources and a lack of clear guidance. This affected quality of care, particularly with regards to fetal monitoring and neonatal care. Staff reported a need for more support, particularly from hospital management, and training opportunities.

Strengths and Limitations

This study offers an ethnographic account of care for women with pre-eclampsia in hospital in rural Ethiopia. Observational data, triangulated with in-depth interviews alongside relevant health policy and clinical guidelines, enabled a holistic examination of processes and quality of care and the factors challenging them. We explored diverse perspectives including women affected by pre-eclampsia, families of women who did not survive, healthcare providers and managers. This approach allowed a deeper understanding of the context of care including clinical, sociocultural and bureaucratic aspects. Our analysis was informed by participants descriptions and recollections of care they gave or received, as well as observed explicit and tacit actions and interactions. We used the guidelines to contextualise our observations and the accounts of care.

For peer review only

For peer review only - http://bmjopen.bmj.com/site/about/guidelines.xhtml

Conducting a rapid ethnography offered both advantages and disadvantages. We were able to feedback key practical findings from our observational work to staff and district level stakeholders, in a timely way, informing quality improvement initiatives as part of wider health system strengthening efforts. This also offered an opportunity for member checking, ensuring our observations reflected real-world challenges faced by providers. Although longer periods of observation may have revealed further useful insights, recurring themes were evident in field notes. Another limitation was the need for translation for one observer. English was used by staff to document in hospital notes, and all staff spoke English, however interactions with patients and between staff were usually in Amharic. Subtle meaning or nuance may have been lost in translation or interactions misinterpreted. Furthermore, the ‘outsider’ status of a non-Ethiopian team member is likely to have affected behaviour of participants. However, this is a recognised challenge when conducting ethnography and was considered when interpreting findings.[28 29] Staff were friendly, cooperative and keen to share their experiences, and often asked observers for advice or feedback.

Interpretation

For peer review only

In our study early detection, care escalation and appropriate management for pre-eclampsia were undermined by a lack of up-to-date guidance based on evidence. Clinical manifestations and conceptualisations of pre-eclampsia are variable. The International Classification of Diseases (ICD-11) has adopted an updated, broader definition of pre-eclampsia to include hypertension and proteinuria or new-onset maternal organ dysfunction, neurological sequelae or fetal growth restriction.[7 30] Inconsistency remains in national and international clinical practice guidelines implementation of the broad definition and the categorisation of 'severe' pre-eclampsia or pre-eclampsia with severe features.[31] This is important as guidelines for management and recommendations for interventions are linked to classification of the disorder. These inconsistencies were mirrored in the care we observed in this study.

Early prediction and prophylaxis for pre-eclampsia were not a part of routine care in our study site, nor recommended in Ethiopian guidelines. Most Ethiopian women consume insufficient calcium, but large regional variation exists.[32] Implementation of interventions requiring early contact may be challenging in a context where first trimester care is rare, particularly at levels of the system where providers are competent and confident to prescribe. However, policy makers should consider evidence-based guidance and

For peer review only

international recommendations to support prophylaxis and reduce burden of disease in at risk women.[9 33 34] The updated 2020 national guideline does not address these discrepancies.

Accurate measurement of BP is fundamental to detecting and managing pre-eclampsia. In our study sites both fully automated devices and mercury sphygmomanometry, were used. The latter requires skills and training to ensure accuracy. The haemodynamic adaptations in pregnancy and pre-eclampsia affect the oscillometric measurement used in automated devices. Few automated devices have been validated in pregnancy or pre-eclampsia. The use of unvalidated or uncalibrated automated devices may lead to significant inaccuracies in blood pressure measurement.[35] Accurate and cost-effective devices have been validated in pre-eclampsia and are useful in health systems where low-skilled staff are involved in maternal care.[36-38]

Triage, recognition of risk and care escalation for pre-eclampsia were identified as areas for improvement in our study. In settings where resources are limited, risk prediction models may offer support to staff to target interventions and decision-making around place of care.[39] Our findings concur with previous studies reporting gaps in knowledge and practices

For peer review only - http://bmjopen.bmj.com/site/about/guidelines.xhtml

required for early detection and management of pre-eclampsia in facilities in low-resource settings.[40] Health systems constraints, training, supervision and motivation of staff affect clinical performance and outcomes.

Health planners must focus on ensuring that funding in maternity services and the allocation of resources work to address current challenges to equity. This requires understanding contexts of care and how policies are implemented at a local level. Improvements in external factors such as guidelines and infrastructure, must be mirrored by changes in cultures of care, that support a shared understanding of quality, focus on team competencies and supportive leadership. Previous quality improvement (QI) initiatives in Ethiopia have failed due to lack of understanding and adaptation to local contexts.[41] Understanding formal and informal power structures, considering spheres of influence and creating social and reputational incentives for change may improve focus and engagement. A study aiming to measure and improve quality of intrapartum care, showed that having lead hospitals demonstrate the feasibility of their assessment tool helped to promote its wider acceptance, as other hospitals sought to achieve the national recognition given to the lead hospitals.[42] Participatory approaches that bring together women, clinical and managerial staff may help to shift power dynamics where unconstructive hierarchies exist. Developing shared goals,

For peer review only

strategies to achieve them and clarifying roles early on can improve sustainable and effective implementation.

Conclusions

Quality care for pre-eclampsia requires organisational change to create safe spaces for learning and improvement, alongside efforts to put women at the centre of care and ensure providers are equipped with knowledge, resources and support to adhere to evidence-based practice.

National guidelines for HDP including pre-eclampsia should recommend treatment of BP consistently ≥140/90mmHg to reduce the risk of developing severe hypertension and other complications, and to urgently treat severe hypertension ≥160/110mmHg.[7] They should also include guidance for professionals on counselling women about their future risks, including of having a subsequent pregnancy affected by pre-eclampsia, cardiovascular disease and stroke, and strategies to reduce these. As the growing burden of non-communicable disease emerges globally, opportunities should be seized whilst women are engaged with health services to optimise longer-term outcomes.

For peer review only

More research is required to understand whether evidence established in high resource settings is applicable in LMICs. This should include implementation research and cost-effectiveness of early prediction and prevention strategies.[34]

It is acknowledged that staff are working under difficult conditions within resource constrained systems. This study allows a focus for where to instigate system change to improve the management of pre-eclampsia for women and support staff to deliver quality care. This should include improvements in antenatal screening and detection of hypertension.

Acknowledgements

We gratefully thank all participants for their time and contributions to the study. We wish to thank Sewit Hessebon, Hanna Negussie and Medhanit Getachew.

CH receives support from AMARI as part of the DELTAS Africa Initiative [DEL-15-01. JS is an NIHR Senior Investigator and is supported by the National Institute for Health Research (NIHR) Applied Research Collaboration South London (NIHR ARC South London) at King's College Hospital NHS Foundation Trust.

For peer review only

Competing interests: none

Authorship contributions

TR: Conceptualisation, Methodology, Investigation, Data curation, Formal analysis, Visualisation, Writing - original draft, Writing - reviewing & editing

AHS: Conceptualisation, Funding acquisition, Supervision, Writing - reviewing & editing

JS: Conceptualisation, Funding acquisition, Methodology, Supervision, Writing - reviewing &editing

TEG: Investigation, Formal analysis, Writing - reviewing & editing

RDG: Investigation, Formal analysis, Writing - reviewing & editing

AA: Conceptualisation, Funding acquisition, Writing - reviewing & editing

RM: Conceptualisation, Methodology, Funding acquisition, Supervision, Writing - reviewing& editing

CH: Conceptualisation, Methodology, Funding acquisition, Supervision, Writing - reviewing& editing

Availability of data and materials

For peer review only

The data generated and analysed during this study are not publicly available to protect individual anonymity but may be available from the corresponding author on reasonable request.

Ethical approvals

All methods were carried out in accordance with relevant guidelines and regulations including the Declaration of Helsinki. Ethical approvals were granted by Addis Ababa University (026/18/Psy) and King's College London (LRS-17/18-5526). Institutional level consent was given by hospital management for observational data collection. Interview participants were required to give individual consent.

Funding

This research was funded by the National Institute of Health Research (NIHR) Global Health Research Unit on Health System Strengthening in sub-Saharan Africa, King's College London (GHRU 16/136/54) and CH through an NIHR RIGHT grant (NIHR200842) using UK aid from the UK Government. The views expressed are those of the authors and not necessarily those of the NHS, the NIHR or the Department of Health and Social Care.

For peer review only

For peer review only - http://bmjopen.bmj.com/site/about/guidelines.xhtml

References

1. Say L, Chou D, Gemmill A, et al. Global causes of maternal death: a WHO systematic analysis. *Lancet Glob Health* 2014;2(6):e323-33. doi: 10.1016/S2214-109X(14)70227-X [published Online First: 2014/08/12]

2. Abalos E, Cuesta C, Grosso AL, et al. Global and regional estimates of preeclampsia and eclampsia: a systematic review. *Eur J Obstet Gynecol Reprod Biol* 2013;170(1):1-7. doi: 10.1016/j.ejogrb.2013.05.005 [published Online First: 2013/06/12]

3. Abalos E, Cuesta C, Carroli G, et al. Pre-eclampsia, eclampsia and adverse maternal and perinatal outcomes: a secondary analysis of the World Health Organization Multicountry Survey on Maternal and Newborn Health. *BJOG* 2014;121 Suppl 1:14-24. doi: 10.1111/1471-0528.12629 [published Online First: 2014/03/20]

4. Berhan Y, Berhan A. Causes of maternal mortality in Ethiopia: a significant decline in abortion related death. *Ethiop J Health Sci* 2014;24 Suppl:15-28. doi: 10.4314/ejhs.v24i0.3s [published Online First: 2014/12/10]

5. Mersha AG, Abegaz TM, Seid MA. Maternal and perinatal outcomes of hypertensive disorders of pregnancy in Ethiopia: systematic review and meta-analysis. *BMC Pregnancy Childbirth* 2019;19(1):458. doi: 10.1186/s12884-019-2617-8 [published Online First: 2019/12/05]

For peer review only

For peer review only - http://bmjopen.bmj.com/site/about/guidelines.xhtml

6. Knight M NM, Tuffnell D, Kenyon S, Shakespeare J, Brocklehurst P, Kurinczuk JJ (Eds.) on behalf of MBRRACE-UK. . Saving Lives, Improving Mothers' Care - Surveillance of maternal deaths in the UK 2012-14 and lesson learned to inform maternity care for the UK and Ireland Confidential Enquiries into Maternal Deaths and Morbidity 2009-14.: Oxford: National Perinatal Epidemiology Unity, University of Oxford 2016, 2016.

7. Brown MA, Magee LA, Kenny LC, et al. Hypertensive Disorders of Pregnancy: ISSHP Classification, Diagnosis, and Management Recommendations for International Practice. *Hypertension* 2018;72(1):24-43. doi: 10.1161/HYPERTENSIONAHA.117.10803 [published Online First: 2018/06/15]

8. NICE. Hypertension in pregnancy: diagnosis and management: National Institute for Health and Care Excellence; 2019 [Available from: https://www.nice.org.uk/guidance/ng133.

9. Rolnik DL, Wright D, Poon LC, et al. Aspirin versus Placebo in Pregnancies at High Risk for Preterm Preeclampsia. *N Engl J Med* 2017;377(7):613-22. doi: 10.1056/NEJMoa1704559 [published Online First: 2017/06/29]

10. Hofmeyr GJ, Lawrie TA, Atallah AN, et al. Calcium supplementation during pregnancy for preventing hypertensive disorders and related problems. *Cochrane Database*

For peer review only

For peer review only - http://bmjopen.bmj.com/site/about/guidelines.xhtml

Syst Rev 2018;10:CD001059. doi: 10.1002/14651858.CD001059.pub5 [published Online First: 2018/10/03]

11. Magee LA, von Dadelszen P, Rey E, et al. Less-tight versus tight control of hypertension in pregnancy. *N Engl J Med* 2015;372(5):407-17. doi: 10.1056/NEJMoa1404595 [published Online First: 2015/01/30]

12. Abalos E, Duley L, Steyn DW, et al. Antihypertensive drug therapy for mild to moderate hypertension during pregnancy. *Cochrane Database Syst Rev* 2018;10:CD002252. doi: 10.1002/14651858.CD002252.pub4 [published Online First: 2018/10/03]

13. Which anticonvulsant for women with eclampsia? Evidence from the Collaborative Eclampsia Trial. *Lancet* 1995;345(8963):1455-63. [published Online First: 1995/06/10]

14. Duley L, Gulmezoglu AM, Henderson-Smart DJ, et al. Magnesium sulphate and other anticonvulsants for women with pre-eclampsia. *Cochrane Database Syst Rev* 2010(11):CD000025. doi: 10.1002/14651858.CD000025.pub2 [published Online First: 2010/11/12]

15. Roder-DeWan S, Nimako K, Twum-Danso NAY, et al. Health system redesign for maternal and newborn survival: rethinking care models to close the global equity

For peer review only

gap. *BMJ Glob Health* 2020;5(10) doi: 10.1136/bmjgh-2020-002539 [published Online First: 2020/10/16]

16. Hanson C, Waiswa P, Pembe A, et al. Health system redesign for equity in maternal and newborn health must be codesigned, country led, adapted to context and fit for purpose. *BMJ Glob Health* 2020;5(10) doi: 10.1136/bmjgh-2020-003748 [published Online First: 2020/10/16]

17. Kruk ME, Gage AD, Arsenault C, et al. High-quality health systems in the Sustainable Development Goals era: time for a revolution. *Lancet Glob Health* 2018;6(11):e1196-e252. doi: 10.1016/S2214-109X(18)30386-3 [published Online First: 2018/09/10]

18. Vousden N, Lawley E, Seed PT, et al. Incidence of eclampsia and related complications across 10 low- and middle-resource geographical regions: Secondary analysis of a cluster randomised controlled trial. *PLoS Med* 2019;16(3):e1002775. doi: 10.1371/journal.pmed.1002775 [published Online First: 2019/03/30]

19. WHO U, UNFPA, World Bank Group and the United Nations Population Division. Maternal mortality in 2000-2017: Internationally comparable MMR estimates by the Maternal Mortality Estimation Inter-Agency Group (MMEIG); 2019 [Available from: https://www.who.int/gho/maternal_health/countries/eth.pdf.

For peer review only - http://bmjopen.bmj.com/site/about/guidelines.xhtml

20. Ethiopia FMoH. Health Sector Transformation Plan II. https://www.familyplanning2020.org/sites/default/files/HSTP-II.pdf, 2021.

21. Vindrola-Padros C, Vindrola-Padros B. Quick and dirty? A systematic review of the use of rapid ethnographies in healthcare organisation and delivery. *BMJ Qual Saf* 2018;27(4):321-30. doi: 10.1136/bmjqs-2017-007226 [published Online First: 2017/12/22]

22. Braun V, Clarke V. Using thematic analysis in pyschology. *Qualitative Research in Pyschology* 2006;3:2, 77-101 doi: 10.1191/1478088706qp063oa

23. Bohren MA, Hunter EC, Munthe-Kaas HM, et al. Facilitators and barriers to facility-based delivery in low- and middle-income countries: a qualitative evidence synthesis. *Reprod Health* 2014;11(1):71. doi: 10.1186/1742-4755-11-71 [published Online First: 2014/09/23]

24. Bohren MA, Mehrtash H, Fawole B, et al. How women are treated during facility-based childbirth in four countries: a cross-sectional study with labour observations and community-based surveys. *Lancet* 2019;394(10210):1750-63. doi: 10.1016/S0140-6736(19)31992-0 [published Online First: 2019/10/13]

25. Bradley S, McCourt C, Rayment J, et al. Disrespectful intrapartum care during facility-based delivery in sub-Saharan Africa: A qualitative systematic review and thematic

For peer review only

For peer review only - http://bmjopen.bmj.com/site/about/guidelines.xhtml

synthesis of women's perceptions and experiences. *Soc Sci Med* 2016;169:157-70. doi: 10.1016/j.socscimed.2016.09.039 [published Online First: 2016/10/23]

26. Burrowes S, Holcombe SJ, Jara D, et al. Midwives' and patients' perspectives on disrespect and abuse during labor and delivery care in Ethiopia: a qualitative study. *BMC Pregnancy Childbirth* 2017;17(1):263. doi: 10.1186/s12884-017-1442-1 [published Online First: 2017/08/24]

27. Knight HE, Self A, Kennedy SH. Why are women dying when they reach hospital on time? A systematic review of the 'third delay'. *PLoS One* 2013;8(5):e63846. doi: 10.1371/journal.pone.0063846 [published Online First: 2013/05/25]

28. Dixon-Woods M. What can ethnography do for quality and safety in health care? *Qual Saf Health Care* 2003;12(5):326-7. doi: 10.1136/qhc.12.5.326 [published Online First: 2003/10/09]

29. Cupit C, Mackintosh N, Armstrong N. Using ethnography to study improving healthcare: reflections on the 'ethnographic' label. *BMJ Qual Saf* 2018;27(4):258-60. doi: 10.1136/bmjqs-2017-007599 [published Online First: 2018/02/22]

30. WHO. International Classification of Diseases (ICD-11) [Available from: https://www.who.int/standards/classifications/classification-of-diseases2019.

For peer review only - http://bmjopen.bmj.com/site/about/guidelines.xhtml

31. Scott G, Gillon TE, Pels A, et al. Guidelines-similarities and dissimilarities: a systematic review of international clinical practice guidelines for pregnancy hypertension. *Am J Obstet Gynecol* 2020 doi: 10.1016/j.ajog.2020.08.018 [published Online First: 2020/08/24]

32. Tesfaye B, Sinclair K, Wuehler SE, et al. Applying international guidelines for calcium supplementation to prevent pre-eclampsia: simulation of recommended dosages suggests risk of excess intake in Ethiopia. *Public Health Nutr* 2018:1-11. doi: 10.1017/S1368980018002562 [published Online First: 2018/10/16]

33. WHO recommendation: calcium supplementation during pregnancy for the prevention of pre-eclampsia and its complications. Geneva: World Health Organisation, 2018.

34. Poon LC, Shennan A, Hyett JA, et al. The International Federation of Gynecology and Obstetrics (FIGO) initiative on pre-eclampsia: A pragmatic guide for first-trimester screening and prevention. *Int J Gynaecol Obstet* 2019;145 Suppl 1:1-33. doi: 10.1002/ijgo.12802 [published Online First: 2019/05/22]

35. Gupta M, Shennan AH, Halligan A, et al. Accuracy of oscillometric blood pressure monitoring in pregnancy and pre-eclampsia. *Br J Obstet Gynaecol* 1997;104(3):350-5. doi: 10.1111/j.1471-0528.1997.tb11467.x [published Online First: 1997/03/01]

For peer review only

36. Nathan HL, de Greeff A, Hezelgrave NL, et al. An accurate semiautomated oscillometric blood pressure device for use in pregnancy (including pre-eclampsia) in a low-income and middle-income country population: the Microlife 3AS1-2. *Blood Press Monit* 2015;20(1):52-5. doi: 10.1097/MBP.0000000000000086 [published Online First: 2014/09/23]

37. Nathan HL, Vousden N, Lawley E, et al. Development and evaluation of a novel Vital Signs Alert device for use in pregnancy in low-resource settings. *BMJ Innov* 2018;4(4):192-98. doi: 10.1136/bmjinnov-2017-000235 [published Online First: 2018/10/16]

38. Nathan HL, Boene H, Munguambe K, et al. The CRADLE vital signs alert: qualitative evaluation of a novel device designed for use in pregnancy by healthcare workers in low-resource settings. *Reprod Health* 2018;15(1):5. doi: 10.1186/s12978-017-0450-y [published Online First: 2018/01/07]

39. Payne BA, Hutcheon JA, Ansermino JM, et al. A risk prediction model for the assessment and triage of women with hypertensive disorders of pregnancy in low-resourced settings: the miniPIERS (Pre-eclampsia Integrated Estimate of RiSk) multi-country prospective cohort study. *PLoS Med* 2014;11(1):e1001589. doi: 10.1371/journal.pmed.1001589 [published Online First: 2014/01/28]

For peer review only

40. Ansari N, Manalai P, Maruf F, et al. Quality of care in early detection and management of pre-eclampsia/eclampsia in health facilities in Afghanistan. *BMC Pregnancy Childbirth* 2019;19(1):36. doi: 10.1186/s12884-018-2143-0 [published Online First: 2019/01/20]

41. Aveling EL, Zegeye DT, Silverman M. Obstacles to implementation of an intervention to improve surgical services in an Ethiopian hospital: a qualitative study of an international health partnership project. *BMC Health Serv Res* 2016;16(1):393. doi: 10.1186/s12913-016-1639-4 [published Online First: 2016/08/18]

42. Canavan ME, Brault MA, Tatek D, et al. Maternal and neonatal services in Ethiopia: measuring and improving quality. *Bull World Health Organ* 2017;95(6):473-77. doi: 10.2471/BLT.16.178806 [published Online First: 2017/06/13]

Figure Legend:

Figure 1. Ethiopian Health System Structure

For peer review only

Figure 1. Ethiopian Health System Structure [20]

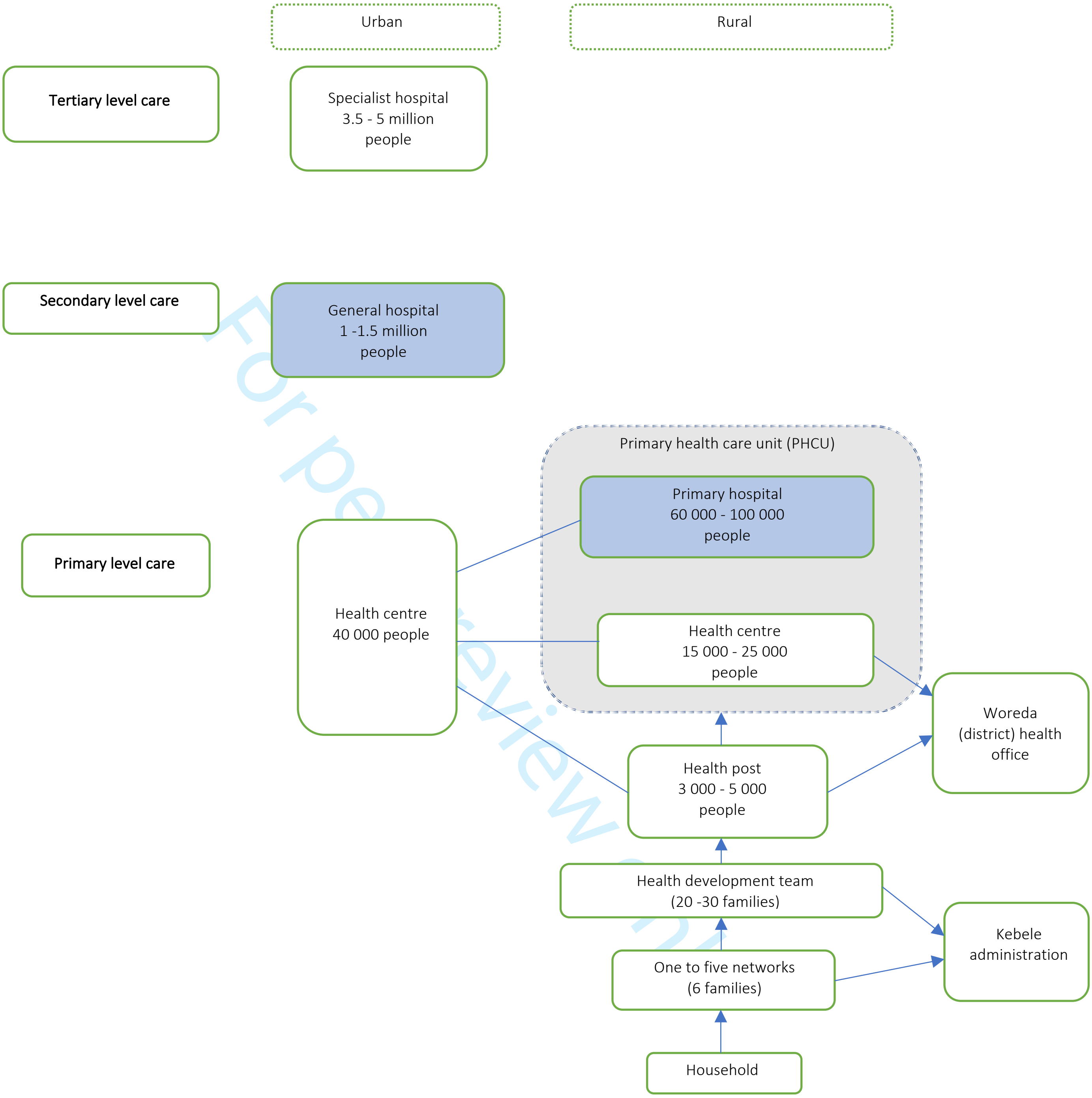

Codebook

Nodes\\Synthesis 1_TR_TE_combined\\Synthesis 2_themes_V1.0_2\\Synthesis 3

Name	Description	Files	References
1.0 Inconsistencies and inequity_Analysis 1	Inconsistency in how the 'rules' are applied may exacerbate inequities but may also endeavour to ameliorate them	0	0
Clinical factors		0	0
Clinical competence	examples of factors affecting ability to provide safe care. Relates to ability to accurately assess and manage patients. May relate to a lack of awareness, knowledge, training or supervision.	25	48
Clinical uncertainty	Explicit or implicit uncertainty or variance in practice. May relate to decision-making, processes of care, deviance from recommendations in guidelines, complexity in clinical case.	25	43
Recognition of risk or problems	Issues relating to identification of increased risk of complications or need for surveillance/action. Ability to perceive problems.	31	89
Theoretical processes of care_pre-eclampsia	Pathways or processes for how patients with high blood pressure or pre-eclampsia move between levels within or between health facilities, in theory (as opposed to what actually happens/is observed).	11	22
Quality issues		0	0
Adherence to guidelines	Examples of how care relates to national or local guidelines	22	55
Communication between staff	How, when and why staff communicate with each other	23	66
Communication with patients and family	Any communications or verbal interactions with patients or their families, either directly observed or described. The content of, why, when or how, communication with patients and their attendants.	24	89
Conflict between staff	Includes disagreements, differences in opinions or different approaches to problems or situations	10	15

Name	Description	Files	References
Conscientiousness	Healthcare professionals being diligent and willing to work efficiently	22	41
Consent	Permission to medical treatments, examinations or procedures. May include examples of consent taken/given or not taken/given. May be verbal or written or sometimes implied e.g holding out arm for a blood pressure check.	12	18
Detection or surveillance_pre-eclampsia	Any observations relating to detection of pre-eclampsia including BP monitoring, screening urine or blood tests for end organ damage.	22	54
Quality of documentation	use of written records to note down care given including monitoring (checking vital signs), investigations, treatments, diagnoses, patient details.	27	98
Timely management_pre-eclampsia	Observations or descriptions of process or sequence of care relating to pre-eclampsia, factors relating to reducing or contributing to delays in care for pre-eclampsia or management of high blood pressure.	6	13
Transparency and accountability	Observations or descriptions relating to (un)ethical professional practice, recognition of errors, appropriate action taken. Relates to open communication and good governance. Transparency relates to ensuring information is available to patients and management, to protect patient safety. Accountability to ensure providers/facilities are held responsible for actions or consequences of errors.	19	50
Structural or systems barriers and facilitators		0	0
Access to medications	Issues relating to availability or lack of medications within hospital pharmacy. Relates to organisation with pharmacy, procurement, supply chains and financing.	9	21
Availability and awareness of guidelines	relates to providers/healthcare workers awareness of relevant clinical guidelines and their availability in clinical areas for use	13	26
Availability and utilisation of WASH	Water And Sanitation Hygiene. Availability, access and utilisation of water and sanitation equipment including soap.	14	22
Cleanliness	General cleanliness of rooms and facility. May include factors relating to patient/family behaviour such as disposal of general waste. And/or behaviours or practices of clinical or cleaning staff.	14	30
Electricity supply	Lack of access or supply of electricity	6	13
Inconsistency in directives or care	Failure to standardised procedures. May be due to changes in advice from	16	27

For peer review only - http://bmjopen.bmj.com/site/about/guidelines.xhtml

Name	Description	Files	References
	management, differences in understanding or interpretation of guidance or rules or unclear guidance.		
Organisation of services	Where distinct services are offered and which cadres of staff cover these services.	15	26
Payment for maternal care services	Details of payments required or made by patients or their families for services not provided for free at the point of care. Out of pocket payments.	16	33
Staffing	Details of numbers, cadres of staff providing care, availability out of hours and access to specialists outside daytime hours or on weekends. Includes issues related to understaffing/lack of required staff numbers for safe or effective practice.	23	62
Support and training for staff	References to professional development or learning and support or need for any of these.	16	57
Triage process and initial management	Any observations or descriptions relating to when patient's first arrive in hospital, initial assessment and prioritisation of patients/conditions. Who does this, where, how. Included details of initial place of care, cadres of health work providing care, initial investigations, assessment and any treatments given immediately.	16	48
Workload	Factors influencing the amount of work and productivity of the facility. Related to utilisation of services and workforce structures and numbers.	22	67
2.0 Improvisation_Analysis 1	Making do or tinkering in attempt to mitigate systemic constraints	0	0
Appropriateness of referrals	Factors relating to whether referrals are timely/late AND/OR appropriate or inappropriate in terms of clinical need/level of care at receiving facility AND/OR appropriate communication to receiving hospital in advance	10	16
Availability of clinical investigations	included any lab tests, ultrasound, etc.	12	18
Availability of equipment and materials	Availability of materials and essential equipment e.g gloves, sheets, resuscitation equipment. EXCLUDES medication	28	85
Blood transfusion	Issues relating to availability, access to, planning, supply of blood and blood products including plasma/FFP.	12	29
Clinical decision-making	How, when and where clinicians make decisions about management of patients. e.g whether require admission, involving colleagues/team decision-making	26	53
Decision re mode of delivery	Factors influencing how decisions are made about how to deliver babies e.g	8	12

For peer review only - http://bmjopen.bmj.com/site/about/guidelines.xhtml

Name	Description	Files	References
	Caesarean delivery versus vaginal delivery. Also includes spontaneous vaginal delivery (SVD) versus instrumental birth (ventouse or forceps delivery).		
Decision re timing of delivery	Factors influencing when clinicians may recommend birth, if women present with problems or require intervention.	11	22
Efficiency	How resources, time or people are used to reduce waste. Related to provision of quality care.	13	20
Escalation of care	Processes of referral on to other clinicians or higher level facility, where more skills, knowledge or resources are required in making a clinical assessment or giving treatment.	31	89
Family support	Support from partners or wider family, may be emotional or practical support. Relates to enabling access and provision of appropriate, quality care in terms of patient centred care, timely care if support from family etc.	18	45
Fetal or baby care and monitoring	included observed processes of care or descriptions of how fetal monitoring is carried out including assessment of heart rate or obstetric ultrasound	13	42
Observed processes of care_pre-eclampsia or severe hypertension	Includes observed processes of care for women with severe hypertension (160/110mmHg). Examination, ordering investigations, prescribing medication, admission criteria.	16	36
Observed processes of care_magnesium sulphate	More detailed accounts of magnesium sulphate administration or use including dosing, length of treatment, reasons for initiation or discontinuing.	10	15
Reassessment	rechecking vital signs, clinical condition or investigations. Relates to timely detection of problems, detection of worsening problems, need to escalate and provision of quality care.	18	36
Physical organisation of care	Physical locations within the hospital, designated to particular services and how these are organised.	18	41
Patient flow	the movement of patients through the facility/different stages of care from triage/admission to discharge	5	16
Precarity	Uncertainty or insecurity in care given, may be due to miscommunication or lack of adherence to recommended/evidence-based care.	21	34
Prioritisation	Processes of deciding on relative importance of tasks or problems.	9	19

For peer review only - http://bmjopen.bmj.com/site/about/guidelines.xhtml

Name	Description	Files	References
Privacy	Issues relating to provision of respectful care, including offering to cover patients during examination, providing space for using toilet facilities/bed pan in a space without others around.	18	57
Roles and responsibilities	Descriptions or observations relating to professional roles and responsibilities including particular tasks and duties undertaken.	23	74
Safety	Any issues relating to patient or staff safety including structural issues or processes of care. Includes observations or descriptions of unsafe practice relating to administration of drugs, use of medical devices, unsafe injection practices, hygiene and infection control. Includes appropriate disposal of clinical waste.	20	47
Theoretical processes of care_general	Pathways or processes for how patients move between levels within or between health facilities, in theory (as opposed to what actually happens/is observed). RELATING TO ANY CONDITION	11	17
3.0 Trust and support_Analysis 1		0	0
Acceptance of treatment or advice	Discussion or views about whether patients and/or families accept treatments or management as advised by caregivers. May involve decisional conflict.	20	49
Authority structures	Interactions or examples of hierarchy within the care system. Included both health professionals e.g midwives, nurses, IESO, obstetrician, medical director and auxiliary staff e.g cleaners, lab technicians, and also decision-makers e.g women, partners	16	24
Availability of specialist staff	Access and availability. Includes staff with any specialist skills e.g doctors (obstetrician/gynaecologist), laboratory staff	16	33
Barriers and facilitators to follow up	Challenges that hinder mothers attending for follow up either routine or if notice danger signs/have a problem AND/OR factors that can improve attendance at facility, acceptance of advice or treatments	14	36
Communication with patients and family	Any communications or verbal interactions with patients or their families, either directly observed or described. The content of, why, when or how, communication with patients and their attendants.	24	89
Creating community awareness	Health promotion activities to improve knowledge and awareness of danger signs during pregnancy, birth and postnatally.	14	43
Defensiveness	Behaviour observed or described relating to being anxious or to avoid potential	10	18

Name	Description	Files	References
	criticism.		
Feedback to referring health centres	System or processes to allow information to go back to referring facilities including clinical outcomes, appropriateness of referrals, appropriateness of initial triage/management, documentation, staff accompanying on referral	7	15
Inertia	Tendency to do nothing/not take action when required despite lack of barriers.	13	25
Maintenance of equipment	includes charging equipment, replacement of batteries or broken parts, servicing equipment etc.	10	18
Neglect	Failure to provide quality care or abandonment of patient needs	13	20
Patient concerns	Any issues relating to anxieties or problems expressed by patients or described by those caring for them. Relates to access and quality of care.	23	60
Professional satisfaction	Health workers feelings about their roles, how they are valued, thoughts about pay/compensation for work and incentives	7	16
Self-appraisal of skills	Assessment by individuals of themselves/their own skills/ability or of the facilities within which they work. Self-reflection.	8	19
Team working	Collaboration between professionals to perform duties including examples of joint decision making (included examples of good or poor team working)	11	26
Trust in resources	Relating to confidence in either physical/material or human resources.	15	18
Undermining	Observations or descriptions of behaviour that diminishes power of an individual to act/carry out their responsibilities.	7	7
Use of traditional medicines	Any issues relating to pregnant women using traditional medicines and how this relates to their care in health facilities.	7	13
Women's experience of care or services	includes their perceptions about quality of care in hospital, continuity of carer, information provided to them, how professional communicated, if they were counselled appropriately (general impressions that may not fit into other codes)	7	25

For peer review only - http://bmjopen.bmj.com/site/about/guidelines.xhtml

Consolidated criteria for reporting qualitative studies (COREQ) checklist

Number	Item	Ethiopia	Location in manuscript (section, page number)
**Domain 1: Research team and reflexivity**			
1.	Interviewer/facilitator	RD, TR, TE	Methods, data collection, pg7
2.	Credentials		Methods, data collection, pg7
3.	Occupation	Ethiopian obstetrician, British obstetrician, Social epidemiology research coordinator	Methods, data collection, pg7
4.	Gender	F/F/F	Not included
5.	Experience and training	Prior experience of qualitative data collection	Not included
6.	Relationship established	No	Methods, data collection, pg 8
7.	Participant knowledge of the interviewer	No	Methods, data collection, pg 8
8.	Interviewer characteristics	Involved in wider health system strengthening project	Methods, data collection, pg 7
**Domain 2: study design**			

For peer review only

9.	Methodological orientation and theoretical frameworks	Thematic analysis using an inductive approach	Methods, data analysis, pg 8,9
10.	Sampling	Purposive sampling from routinely collected data	Methods, data collection, pg7
11.	Method of approach	Face to face	Not included
12.	Sample size	21 IDI	Methods, data collection, pg7,8
13.	Non-participations	0	n/a
14.	Setting of data collection	Primary and general hospital	Methods, study setting, pg 6
15.	Presence of non-participants	No	n/a
16.	Description of sample	Yes	Methods, data collection, pg7,8
17.	Topic guide	Example guide included as supplementary file.	n/a
18.	Repeat interviews	No, further sampling where data saturation needed e.g women with previous pre-eclampsia	Not included

For peer review only

19.	Audio recorded	Yes	Methods, data collection, pg 8
20.	Field notes	Yes	Methods, data collection, pg 7
21.	Duration of interviews/FGD	Variable	Not included
22.	Data saturation	Yes	Not included
23.	Transcripts returned for participant checking	No, but member checking via Theory of Change workshops for later stage of wider projects	Methods, data collection, pg 8
**Domain 3: analysis and findings**			
24.	Number of data coders	2	Methods, data analysis, pg9
25.	Description of coding tree	Yes - provided in supplementary file	Not included in main manuscript.
26.	Derivation of themes	Derived from data iteratively	Methods, data analysis, pg 8,9
27.	Software	NVivo 12	Methods, data analysis, pg 8
28.	Participant checking	Yes. See 23.	Methods, data collection, pg 8

For peer review only

29.	Quotations presented	Yes, participant ID provided	Results, pg 9 - 19
30.	Data findings consistent	Yes	Strengths and Limitations, pg4
31.	Clarity of major themes	Yes, quotations to support throughout results section and in Table 3	Results, pg 9 - 19
32.	Clarity of minor themes	Yes, see coding structure in supplementary file	Not included in main manuscript.

For peer review only

BMJ Open

Understanding challenges as they impact on hospital level care for pre-eclampsia in rural Ethiopia: a qualitative study

Journal:	*BMJ Open*
Manuscript ID	bmjopen-2022-061500.R2
Article Type:	Original research
Date Submitted by the Author:	20-Jan-2023
Complete List of Authors:	Robbins, Tanya; King's College London, Department of Women and Children's Health<br>Shennan, Andrew; King's College London, Department of Women and Children's Health, School of Life Course Sciences<br>Sandall, Jane; King's College London, Department of Woman and Children's Health<br>Eshetu Guangul , Tigist; Addis Ababa University College of Health Sciences, Centre for Innovative Drug Development and Therapeutic Trials for Africa (CDT-Africa)<br>Demissew, Rahel; Addis Ababa University, Department of Obstetrics and Gynecology<br>Abdella, Ahmed; Addis Ababa University College of Health Sciences, Department of Obstetrics & Gynaecology<br>Mayston, Rosie; King's College London, Department of Global Health and Social Medicine<br>Hanlon, Charlotte; King's College London, Institute of Psychiatry, Psychology and Neuroscience, Health Service and Population Research Department, Centre for Global Mental Health; Addis Ababa University, Centre for Innovative Drug Development and Therapeutic Trials for Africa (CDT-Africa), College of Health Sciences
**Primary Subject Heading**:	Obstetrics and gynaecology
Secondary Subject Heading:	Global health, Health policy, Qualitative research, Patient-centred medicine
Keywords:	OBSTETRICS, Hypertension < CARDIOLOGY, QUALITATIVE RESEARCH, Change management < HEALTH SERVICES ADMINISTRATION & MANAGEMENT, Health policy < HEALTH SERVICES ADMINISTRATION & MANAGEMENT, Organisation of health services < HEALTH SERVICES ADMINISTRATION & MANAGEMENT

SCHOLARONE™
Manuscripts

I, the Submitting Author has the right to grant and does grant on behalf of all authors of the Work (as defined in the below author licence), an exclusive licence and/or a non-exclusive licence for contributions from authors who are: i) UK Crown employees; ii) where BMJ has agreed a CC-BY licence shall apply, and/or iii) in accordance with the terms applicable for US Federal Government officers or employees acting as part of their official duties; on a worldwide, perpetual, irrevocable, royalty-free basis to BMJ Publishing Group Ltd ("BMJ") its licensees and where the relevant Journal is co-owned by BMJ to the co-owners of the Journal, to publish the Work in this journal and any other BMJ products and to exploit all rights, as set out in our licence.

The Submitting Author accepts and understands that any supply made under these terms is made by BMJ to the Submitting Author unless you are acting as an employee on behalf of your employer or a postgraduate student of an affiliated institution which is paying any applicable article publishing charge ("APC") for Open Access articles. Where the Submitting Author wishes to make the Work available on an Open Access basis (and intends to pay the relevant APC), the terms of reuse of such Open Access shall be governed by a Creative Commons licence – details of these licences and which Creative Commons licence will apply to this Work are set out in our licence referred to above.

Other than as permitted in any relevant BMJ Author's Self Archiving Policies, I confirm this Work has not been accepted for publication elsewhere, is not being considered for publication elsewhere and does not duplicate material already published. I confirm all authors consent to publication of this Work and authorise the granting of this licence.

For peer review only

Title

Understanding challenges as they impact on hospital level care for pre-eclampsia in rural Ethiopia: a qualitative study

Corresponding author details

Tanya Robbins

Department of Women and Children's Health

School of Life Course Science

King's College London

10th Floor, North Wing

St Thomas' Hospital

Westminster Bridge Road

London SE1 7EH

tanya.robbins@kcl.ac.uk

Authors

Tanya Robbins [a] tanya.robbins@kcl.ac.uk

Andrew H Shennan [a] andrew.shennan@kcl.ac.uk

For peer review only

Jane Sandall [a] jane.sandall@kcl.ac.uk

Tigist Eshetu Guangul [b] tigi.eshetu@yahoo.com

Rahel Demissew [c] raheldemissewgy@gmail.com

Ahmed Abdella [c] ahmedamu@yahoo.com

Rosie Mayston [d] rosie.mayston@kcl.ac.uk

Charlotte Hanlon [e, f] charlotte.hanlon@kcl.ac.uk

Affiliations

[a] Department of Women & Children's Health, School of Life Course Sciences, King's College London, St Thomas' Hospital, London, UK

[b] Centre for Innovative Drug Development and Therapeutic Trials for Africa (CDT-Africa), College of Health Sciences, Addis Ababa University, Addis Ababa, Ethiopia

[c] Department of Obstetrics & Gynaecology, College of Health Sciences, Addis Ababa University, Addis Ababa, Ethiopia

[d] Department of Global Health and Social Medicine, King's College London, London, UK

[e] Centre for Global Mental Health, Health Service and Population Research Department, and WHO Collaborating Centre for Mental Health Research and Training, Institute of Psychiatry, Psychology and Neuroscience, King's College London, London, UK

For peer review only

[f] Department of Psychiatry, WHO Collaborating Centre for Mental Health Research and Capacity-Building, School of Medicine, College of Health Sciences, Addis Ababa University, Addis Ababa, Ethiopia

Word count

4868 words

Abstract

Objective

To explore hospital level care for pre-eclampsia in Ethiopia considering the perspectives of those affected and healthcare providers, in order to understand barriers and facilitators to early detection, care escalation and appropriate management.

Setting

A primary and a general hospital in southern Ethiopia.

Participants

For peer review only

Women with lived experience of pre-eclampsia care in hospital, families of women deceased due to pre-eclampsia, midwives, doctors, integrated emergency surgical officers and healthcare managers.

Results

This study identified numerous systemic barriers to provision of quality, person-centred care for pre-eclampsia in hospitals. Individual staff efforts to respond to maternal emergencies were undermined by a lack of consistency in availability of resources and support. The ways in which policies were applied exacerbated inequities in care. Staff improvised as a means of managing with limited material or human resources and knowledge. Social hierarchies and punitive cultures challenged adequacy of communication with women, documentation of care given and supportive environments for quality improvement.

Conclusions

Quality care for pre-eclampsia requires organisational change to create a safe space for learning and improvement, alongside efforts to offer patient-centred care and ensure providers are equipped with knowledge, resources and support to adhere to evidence-based practice.

For peer review only

Strengths and limitations of this study

- Observational data, triangulated with in-depth interviews alongside relevant health policy and clinical guidelines, enabled a holistic examination of processes and quality of care and the factors challenging them.
- A rapid ethnographic approach enabled timely feedback of key practical findings from our observational work to staff and district level stakeholders.

For peer review only - http://bmjopen.bmj.com/site/about/guidelines.xhtml

Introduction

Poor quality maternal care in hospitals contributes significantly to avoidable deaths. Pre-eclampsia is a leading cause of maternal and perinatal mortality and morbidity globally.[1] The greatest burden is borne by low- and middle-income countries (LMICs).[2] There is a dearth of robust national data on incidence, particularly in LMICs. It is estimated that 2-5% of pregnancies globally are affected by pre-eclampsia.[2 3] In Ethiopia, 19% of maternal deaths are attributed to hypertensive disorders of pregnancy (HDP) compared to 14% worldwide.[1 4 5] In addition to these uncertain estimates, there is an increasing burden of non-communicable diseases in LMICs. [6 7] These include chronic hypertension and diabetes which are known to increase the risk of developing pre-eclampsia. Good quality care makes a difference to outcomes; the majority of deaths due to pre-eclampsia are preventable. Less than one in a million women delivering in the UK now dies due to pre-eclampsia, compared to an estimated 783 deaths per million in Ethiopia.[8]

Internationally there is a lack of consensus concerning classification, diagnosis and management of pre-eclampsia.[9] Controversy remains over inpatient versus outpatient management, target blood pressure and timing of delivery for preterm pre-eclampsia.[10] However, there is evidence to support prophylaxis with low-dose aspirin and supplemental

For peer review only - http://bmjopen.bmj.com/site/about/guidelines.xhtml

calcium if dietary intake is low, treatment of severe hypertension (≥160/100mmHg) and use of magnesium sulphate for eclampsia or pre-eclampsia with severe features.[11-16] Prediction and prevention of pre-eclampsia is an important goal in reducing mortality and morbidity. However robust health systems and continuity across different levels of the care pathway are needed to support effective implementation of these interventions.

Recent proposals for rethinking models of maternity care to improve quality and equity have called for increased focus on centralised services and shifting all deliveries to or near hospital settings.[17] Justifications included the limitations of obstetric risk stratification, low quality care in primary level settings and challenges of referral if complications do occur. However, to address equity, redesign must be country-led, context specific and co-designed with a focus on listening to women and strengthening human resources.[18] As poor quality care emerges as a greater barrier to reducing mortality than insufficient access to services, understanding challenges in secondary care and the experiences of women cared for in hospital is critical.[19] A large, international prospective dataset showed that 61% (28.1% within a central referral hospital) of eclampsia cases in Ethiopia occurred within facilities, highlighting the need to improve quality of care as well as surveillance, early detection and access to appropriate care.[20]

For peer review only

The objective of this study was to develop rich contextual understanding of the processes and experiences of hospital level care for pre-eclampsia in rural Ethiopia to inform efforts to improve quality.

Methods

Study setting

This study was conducted in a primary and general hospital in the Gurage Zone, Southern Nations, Nationalities and Peoples' Region (SNNPR). This study setting was selected to include a predominantly rural population, spread across lowland and highland areas. This is to provide generalisability of our findings. Geographical barriers in the area are significant and access to ambulances limited. Ethiopia has an ethnically diverse population of over 112 million, 79% of whom live in rural areas. Government-run health services are structured into three-tiers: primary, secondary and tertiary care. See figure 1. The general hospital at secondary level care serves as a referral center for the primary hospital. In the study setting, maternity services at the primary hospital were provided by midwives, junior doctors referred to as general practitioners (GPs) and integrated emergency surgical officers (IESOs). IESOs

For peer review only

are non-medical clinical staff trained to provide emergency surgical care. In addition to these cadres, the general hospital was also staffed by an obstetrician.

Ethiopia has seen significant reductions in maternal mortality ratios over recent decades from 1030 deaths per 100,000 live births in 2000 to an estimated 401 in 2017.[21] However, institutional delivery rates remain low, estimated at 26% in 2016 and only 43% of pregnant women had four or more antenatal care visits in 2019. The Ethiopian Federal Ministry of Health (FMoH) produces strategies to improve all levels of the health system set out in five-yearly Health Sector Transformation Plans to guide healthcare delivery.[22] These align with current international commitments such as achieving quality, person-centred, respectful and equitable healthcare.

Figure 1. Ethiopian Health System Structure

Data collection

We conducted a qualitative study between January 2019 and January 2020, using unstructured participant observation, semi-structured interviews and document analysis of hospital guidelines and meeting minutes. Our research team included both an Ethiopian

For peer review only

(RD) and a British obstetrician (TR) and an Ethiopian social epidemiology researcher (TE). Two researchers conducted rapid ethnographic observation over 16 days between the primary and general hospital.[23] Observers included a clinician and an Amharic speaker at all times. We observed all aspects of maternity care relevant to pre-eclampsia including spending time on the labour ward, in the emergency department, antenatal clinic, inpatient wards and in the operating theatre. Ethical challenges about when and how to intervene when questionable practices were observed, were discussed between clinical members of the research team. Clinical researchers assisted in life-threatening situations. Where staff explicitly asked for assistance with non-critical cases they were encouraged to continue with routine practice. Informal interviews were conducted with staff to enquire about specific aspects of care or interactions during participant observation where further clarification or context was sought. Contemporaneous field notes were taken and discussed between researchers encouraging reflexive practice. 21 formal in-depth semi-structured interviews were conducted. We purposively sampled 10 staff members (two midwives, three IESOs, four doctors including an obstetrician and a district health manager), eight women with lived experience of pre-eclampsia and three family members of women who had died due to pre-eclampsia. Women affected by pre-eclampsia were identified through routinely collected data. The research team conducting interviews were not known to interviewees. Interviews

For peer review only

For peer review only - http://bmjopen.bmj.com/site/about/guidelines.xhtml

were conducted in Amharic, audio-recorded, transcribed and translated into English. Transcripts were cross-checked by Ethiopian research co-ordinators for accuracy.

Findings from the participant observation were fed back to staff via a meeting to inform later stages of a wider project on health system strengthening. Minutes from this meeting along with national guidelines relating to care for pre-eclampsia were also used as data sources to triangulate findings and provide relevant context.

Clinical care guidelines in use at the time of the study included: FMoH Management Protocol on Selected Obstetric Topics 2010, Standard Treatment Guidelines 2014 (STG) for Primary and General Hospitals and a Comprehensive Emergency Obstetric and Newborn Care Training Manual. The FMoH guidelines advocated for four focused antenatal care (ANC) visits emphasising quality rather than quantity. Usual care for women with low-risk pregnancies was provided at health centres and the other visits conducted by health extension workers (HEWs) at health posts close to women's homes. Pregnant women identified to have risk factors including amongst others, previous pre-eclampsia, chronic hypertension, diabetes or renal disease were, according to national guidelines to be referred to higher level care or offered closer follow up.

For peer review only

Data analysis

We undertook thematic analysis using an inductive approach[24]. Field notes, interview transcripts and the minutes of the meeting were uploaded into NVivo12 software. Data were read and re-read; and initial codes were generated, reviewed and refined by TR, TE and CH (see supplementary file 1). All data were then recoded by TR and themes generated and reviewed with CH and RD. Themes and subthemes were checked against the data to examine relationships between them.

Patient and public involvement

The design of this study focussed on examining the lived experience of women affected by pre-eclampsia and their families. We included formal and informal interviews with women. Our research questions were informed by endeavouring to understand the priorities and experiences of patients. They were not involved in the planning, design or analysis of this study.

Results

For peer review only

Women and healthcare providers described significant systemic barriers to the provision of quality care. Basic structural necessities such a running water, soap and electricity were intermittently unavailable. This was particularly evident at the primary hospital. During four days of observation at the primary hospital there was no running water. There were no sinks in any of the inpatient wards or clinical areas, including the infection prevention room at the primary hospital.

Our analysis revealed contextual challenges that women and professionals faced, within which they sought to receive or provide quality care for pre-eclampsia. We present three main themes: inconsistencies and inequity, improvisation and making do, and trust and support.

Theme I: Inconsistencies and inequity

Key guidelines identified by staff as informing care for pre-eclampsia at hospital level were acknowledged by some professionals as being outdated and were adhered to variably. Women admitted to hospital with pre-eclampsia were often managed according to the national guideline but not consistently. Variability in clinical practice related to incomplete clinical assessments, including not eliciting or documenting significant risk factors such as a

For peer review only

history of eclampsia and previous stillbirth, and a lack of knowledge, for example, of established thresholds of severe hypertension.

> *"She will be given magnesium sulphate (MgSO4) if her systolic BP is above 180 or diastolic above 110, we will give her hydralazine to drop the elevated BP."*
>
> (Midwife, M11)

Guidelines and practice were ambiguous relating to the management of antenatal women presenting with, or at risk of eclampsia, who were preterm and whose blood pressure (BP) normalised. Staff reported that women were discharged prior to delivery if their BP was controlled with antihypertensives.

> *"Q: She came with eclampsia; under what condition you will decide and discharge her?*
>
> *A: When her BP become normal, and her protein level reduces. It will become normal when we give them antihypertensive. There are many mothers who are discharged."*
>
> (Midwife, M11)

For peer review only

Some medical staff reported they used diazepam to control seizures despite the availability of magnesium sulphate and guidelines stating this as the drug of choice.

Women described concerns about competence and safety. Some were particularly worried about the danger this posed to more vulnerable, less educated women who may be unable to advocate for themselves. A family member of a woman who died described conflicted feelings, both troubled by the care received whilst recognising providers' motivations were honourable.

> *"I have seen a lot there, we or you may read English a little and yes, your parents may read Amharic but how about those women who do not know anything. Yes, mistake a lot there, especially practitioners who provide medicine at resting room after surgery if they are not very careful, I do not know who will make them accountable."*

(Woman with previous pre-eclampsia, M28)

For peer review only

"In this hospital, they don't cooperate. We are grumbling because of them. We are blaming the hospital for her death…Yes, her body refused to circulate someone else's blood and get swelled.…I am not better than the health professionals; they were trying to save her not to kill her…yes…I am thinking this was because I am hurt not because I am better than the health professionals."

(Family of deceased woman, M26)

A limited understanding of the aetiology of pre-eclampsia was reflected in the advice given to women regarding their diagnosis.

"They used to tell me not to drink soft drinks, tea and the like. And that I don't have to use too much salt and need to decrease my fluid intake until the blood pressure goes down."

(Woman with previous pre-eclampsia, M37)

Inconsistencies in triage of pregnant women related to a lack of skills, knowledge or experience of staff. Inappropriate triage was reported to result in a failure to recognise risk, escalate and manage complications appropriately.

For peer review only

For peer review only - http://bmjopen.bmj.com/site/about/guidelines.xhtml

"One of the things that needs improvement is developing the health professionals' skill and knowledge. Most of the problems are happening because of lack of knowledge. Most things are happening by not considering as a problem when actually problem has happened to mothers. Therefore, by improving the awareness we can change many things."

(Senior Clinician, M32)

"Sometimes triage might not be done appropriately, or due to limited knowledge and skill gap, the general practitioner might miss some cases. But once the surgeon is involved, he will be fully responsible."

(Medical Director, M49)

Relevant to the variation in clinical practice and competence, were inadequate and inequitable access to training opportunities and professional development as described by providers. Some staff linked this to poor support from the hospital administration, whilst a senior clinician perceived a professional culture lacking in motivation to pursue independent study and continuing professional development.

For peer review only

"Most of the time, there are no trainings. The process of informing new information and participating the health professionals on training is very poor. This is one of the problem of administrators. Sometimes there are various types of training opportunities for their health professionals or the administrators will train the health professionals on areas where they believe there is a gap. They may also forbid the health professionals that was requested to get the training by paper from the high-level officials. This is the main problem of the administrators in our health institutions. The training does not have to be for getting the per diem rather it has to be used to updating the knowledge and to apply what you have got from the training. The way of administrators supporting this is very poor."

(IESO, M48)

"The first thing is when there are such trainings, our society as a whole have a very poor reading habit. People don't sit down and read the guideline."

(Medical Director, M49)

For peer review only

Inconsistencies in how policies were implemented were also observed and reported. This variation may have been intended to ameliorate inequities in the system but also exacerbated them. National level policy for free maternal care was implemented differentially dependant on clinical situation. In this study setting, women admitted to hospital with complications such as pre-eclampsia, requiring inpatient care but not delivery, were required to pay for services. Women attending purely for delivery received free care. Whereas women with potentially life-threatening complications requiring several medications, longer inpatient stays and at higher risk of delivering preterm infants in need of neonatal care, who were able to, were required to pay. However, women from low socio-economic status households could be considered for exemption by the hospital management or could access free care if community-based health insurance had been rolled out in their area. Nonetheless, out-of-pocket payments for antihypertensive drugs were common. Financial barriers were observed to present tangible challenges for women with potentially serious complications related to pregnancy. Clinicians were not always aware of policy or how to apply it in practice.

Case vignette

For peer review only

A recently postpartum woman presented to the emergency department with significant pitting oedema to bilateral lower limbs. She was reviewed by the doctor on-call and laboratory investigations were requested. She attended the lab but was asked for 160 birr (£2.80) which she was unable to pay. She did not have a community-based health insurance card. The GP asked her to go back to her village, to collect the insurance card, then to visit the local health centre and ask for referral back to the hospital for a full assessment. She did not return to the hospital that day.

> *"It is not free whether she is here or discharged. The hospital will fulfil things required for delivery, like gloves and others. The mothers may not buy the medication since they may not have money; there are mothers who don't have any money. Thus, even if we told them to buy it, they might not buy the medication… like antihypertensive, they have to pay…Yes, she has to purchase it. If methyldopa or so on is prescribed to her at ANC, she has to purchase it from her own pocket… It would have been good if it is possible to make laboratory services and medications prescribed for the mothers free as well. They would have come if it is free"*

(Midwife, M11)

For peer review only

"The problem that we have in this hospital is, sometimes after deciding to admit them, there is some kind of problem on the payment system. For example, if a pregnant woman is close to due date and or going to give birth after some days and is going to be admitted, she is not going to pay. But if she has been admitted not for delivery, she would pay. There are these kinds of unclear rules. If it is free for one person, it should be free for all."

(Senior Clinician, M32)

Theme II: Improvisation and making do

Improvisation emerged as a means of managing or making do with limited resources, both material and human, within both hospitals. This centred around a lack of equipment, medications, staff and support. The barriers and facilitators to early detection, care escalation and appropriate management of pre-eclampsia are described in table 1.

BP monitors were lacking in both hospitals. Staff made-do by sharing, negotiating use between departments or improvising with inadequate equipment such as a plastic training stethoscope used with a sphygmomanometer. At the primary hospital, there was no BP

For peer review only

monitor available in the antenatal clinic. If midwives felt a BP check was needed, the woman was escorted to the ward to have this done.

Midwives at the general hospital made do with one semi-functioning cardiotocograph machine between six beds in the “first stage room” and two delivery beds. Midwives could only assess the current heart rate pattern, or sit with women and observe in real-time, as paper was not available to record. Improvisation with regards to fetal monitoring for high-risk woman was common and related to the lack of resources, but also a lack of knowledge of normal parameters and difficulties interpreting fetal heart rate patterns. Failure to recognise abnormal patterns was observed, which led to delays in taking action, either to expedite delivery or escalate. No fetal monitoring took place in the second stage of labour, regardless of earlier concerns. The reasons for this were difficult to ascertain but this was an established culture of practice. On occasions, the management of suspected fetal distress at the general hospital involved the use of 40% dextrose infusions to the mother. These practices may have been related to a lack of specific guidance for midwives on the management of fetal distress.

For peer review only

Assessment of antenatal women presenting with reduced fetal movements close to term, were inadequate. The fetal heart was auscultated to confirm presence. Women were not offered blood pressure or urine screening, assessment of symphysial fundal height or a growth scan. At the primary hospital no continuous fetal monitoring was available and a diagnosis of presumed fetal distress was made following auscultation of decelerations using a fetoscope.

Precarious practices were observed such as the use of fundal pressure during the second stage of labour when providers lacked confidence, the necessary skills or support to perform assisted delivery.

Families offered frequent support to plug gaps in the system, collecting patient notes, requesting blood tests, clearing buckets of urine and blood on labour ward, procuring drugs and equipment from private pharmacies when not available in labour, and providing support for women in labour when allowed.

Table 1. Barriers and facilitators to early detection, care escalation and appropriate management of pre-eclampsia in hospital

For peer review only

Hospital level care for pre-eclampsia	Barriers	Facilitators
Early detection and recognition of risk	• A lack of continuity of care and linkage between general outpatient department (OPD) clinics where women with chronic hypertension, diabetes or renal disease may be managed and high-risk ANC clinic<br>• Unclear guidelines for midwives, IESOs and doctors regarding outpatient management of women referred from lower levels of care with risk factors for pre-eclampsia<br>• A lack of BP monitors in clinical areas where pregnant women were cared for	• Regular blood pressure monitoring was observed for women admitted with severe hypertension (although not always documented)
Escalation of care	• Staff responsible for triaging pregnant women were not always competent, experienced or supported enough to escalate care appropriately<br>• Poor documentation of clinical history, risk factors, examination and care given challenged communication between staff and different levels of the care pathway	• Referrals from lower levels of care (health centres and primary hospital) were reported to be frequent (although reported by staff as not always necessary)<br>• A clear professional hierarchy was described by staff, indicating they knew who they could refer on to
Appropriate management	• National guidelines were not evidence-based or up to date or were unclear, recommending precarious or potentially unsafe practices<br>- Management of hypertension (explicit advice not to treat non-severe hypertension ≥140/90 to 159/109mmHg, lack of timely management of severe hypertension -advice to wait one hour)	• Magnesium sulphate was available, and doses prescribed according to national and international guidelines<br>• Guidelines recommend delivery within 24 hours of onset of symptoms in “severe pre-eclampsia” and within 12 hours of eclampsia.

For peer review only

	- Timing of delivery (recommend delivery when cervix favourable for “mild pre-eclampsia”, advise avoiding post-term pregnancy but no explicit recommendation for delivery at 37 weeks)<br>• A lack of fluid balance monitoring was observed, and injudicious use of intravenous fluids concurrently with furosemide without a clear indication<br>• Timing of antenatal steroids for fetal lung maturation (given to women with preterm pregnancy and hypertension even when delivery was not planned)<br>• Unclear and inconsistent processes for induction of labour, increasing the risk of caesarean section.<br>• Poor documentation of clinical care led to a lack of transparency and accountability<br>• Minimal counselling regarding future risks including in subsequent pregnancies, increased risk of cardiovascular disease and stroke, and no guideline to support this practice	

Theme III: Trust and support

Communication skills and styles varied between individuals with examples of kind, compassionate interactions, professionals checking women’s understanding and providing reassurance. However, a culture of informed consent and shared decision-making was not widespread. Women were frequently subjected to vaginal examinations in an open ward

For peer review only

without privacy. There were observed examples of abrasive interactions and failures to take women's concerns seriously. Decisions about termination of pregnancy for women with early onset pre-eclampsia were made without explanation to women and were not documented in hospital notes. These interactions were described as "oral reviews". Staff were frequently observed carrying out clinical activities without documenting including performing vaginal examinations, ward rounds and administering drugs. Some staff reported this related to care being given outside prescribed protocols, for example, the timing of examinations during labour. Poor quality documentation was also linked to a punitive culture and fear of being blamed for adverse outcomes. Strategies to improve documentation were in progress including a documentation quality audit.

Allowing women family support during labour was inconsistent and limited by structural constraints such as the lack of privacy and space in the labour ward. However, relating back to the initial theme, inequitable application of the rules left women who were less able to advocate for themselves alone and lacking support, whilst others were allowed family members with them. Staff talked about the challenges they felt affected their ability to provide respectful care including workload and not feeling respected themselves.

For peer review only

There were examples of low staff satisfaction reported by women, affecting quality of care. Staff reported low level of professional support from the hospital administration and a sense of moral duty driving their commitment to work. Suggestions about how to support and retain staff reflected a need to develop more nurturing systems with opportunities for feedback to clinicians, both positive and negative. The theme of trust and support is further illustrated in table 2.

Table 2. Theme III: Trust and support

Theme III: Trust and Support	Verbatim quotes
Women experienced or witnessed lack of respectful care or staff attitudes that presented barriers to quality care	*"But there are also doctors who think they know everything, but they are below doctors. To your surprise, you see it many times, those who insult, who say you did not follow here so go away. But it has no problem. She enters for delivery after signing for the risk, right. She will be responsible for everything that could happen to her. But saying go away while she is about to give birth and because she did not follow there, I have seen a lot."* (Woman with previous pre-eclampsia, M28)
Communication with women affected what they understood about their diagnosis and their trust in professionals and the care received	*"I am still not sure whether I do have or don't have the hypertension at that time. Finally, at the hospital I wasn't so sure whether I have hypertension or not but at the hospital, they said, "It is very much elevated, and she might get into a bad condition if we are waiting more than this". Then they made me sign and performed the surgery and I gave birth. That's all about it. I didn't understand whether they did that purposely to intimidate me or whether I exactly do have the hypertension"*

For peer review only

	(Woman with previous pre-eclampsia, M34)
Doubt and anxiety about their diagnosis led to some women searching for alternative opinions	*"Yes, it was at the hospital. I went three places to check my blood as a matter of fact… they told me to wait and I went to other places in the meantime to get my BP checked… When I checked there, the number was the same. So, I returned back to the hospital because I was told to remain there. I was very nervous."* (Woman with previous pre-eclampsia, M25)
Staff felt challenged to provide compassionate and respectful care	*"Due to the labour pain labouring mothers are aggressive sometimes which makes our job a little bit complicated while giving the service. At this moment it is difficult to practice the CRC [compassionate respectful care] as we hoped to. But you have to minimize your own issue and try to help the mother and her child. We should take care of the child regardless of the situation and the protocol sometimes."* (Head midwife, M33)
Women reported staff were dissatisfied and this affected quality of care and communication	*"I hated many of the women. I am busy and I work here unwillingly [the midwife said]. It is her work, right. She gets paid, right. No one is doing one's job happily. She has to communicate peacefully even if she doesn't like the woman's blood or waste."* (Woman with previous pre-eclampsia, M28)
Staff did not feel supported but were motivated to work by a sense of duty	*"Support is very low. You work for your conscience. There are a lot of problems on the administration side. You just work because of feeling responsible."* (IESO, M47)
Staff reported the need for more feedback as well as support to improve retention	*"Giving recognition for the tasks that they have accomplished well. Appreciating or congratulating on a job well done and give constructive criticism when the work lacks will be great. If not, that person will be compelled to seek other more satisfying work environments."* (IESO, M47)

433

434 **Discussion**

For peer review only

This study found the provision of quality, person-centred care for pre-eclampsia in two Ethiopian hospitals was challenged by numerous systemic barriers. A lack of consistency in availability of resources, support and how policies were applied exacerbated inequities in care. This was particularly evident in our examples where women required admission and investigations for complications during pregnancy or the postnatal period but were unable to pay. Understanding structural drivers of poor-quality maternity care and mistreatment is vital to ameliorating the detrimental impact on future help-seeking.[25 26] There is a need to strengthen education and support for staff to provide respectful, person-centred care as well as addressing resource shortages.[27-29]

Variations in clinical competence, knowledge and experience meant staff providing maternity care were not always able to recognise risks or act on them. Outdated, ambiguous guidelines and inequitable opportunities for professional development exacerbated clinical uncertainty and practice not based on evidence. National guidelines specify against the use of antihypertensives for non-severe hypertension and many women with significant hypertension were not treated. Inadequate documentation did not always reflect clinical risks, drugs administered, or care given. Poor quality documentation was linked to the provision of care divergent from protocol and fear of blame for poor outcomes. A lack of

For peer review only - http://bmjopen.bmj.com/site/about/guidelines.xhtml

transparency, accountability and systems for support were observed and reported. Improvisation was used to address shortages in resources and a lack of clear guidance. This affected quality of care, particularly with regards to fetal monitoring and neonatal care. Staff reported a need for more support, particularly from hospital management, and training opportunities.

Strengths and Limitations

This study offers an ethnographic account of care for women with pre-eclampsia in hospital in rural Ethiopia. Observational data, triangulated with in-depth interviews alongside relevant health policy and clinical guidelines, enabled a holistic examination of processes and quality of care and the factors challenging them. We explored diverse perspectives including women affected by pre-eclampsia, families of women who did not survive, healthcare providers and managers. This approach allowed a deeper understanding of the context of care including clinical, sociocultural and bureaucratic aspects. Our analysis was informed by participants descriptions and recollections of care they gave or received, as well as observed explicit and tacit actions and interactions. We used the guidelines to contextualise our observations and the accounts of care.

For peer review only

Conducting a rapid ethnography offered both advantages and disadvantages. We were able to feedback key practical findings from our observational work to staff and district level stakeholders, in a timely way, informing quality improvement initiatives as part of wider health system strengthening efforts. This also offered an opportunity for member checking, ensuring our observations reflected real-world challenges faced by providers. Although longer periods of observation may have revealed further useful insights, recurring themes were evident in field notes. Another limitation was the need for translation for one observer. English was used by staff to document in hospital notes, and all staff spoke English, however interactions with patients and between staff were usually in Amharic. Subtle meaning or nuance may have been lost in translation or interactions misinterpreted. Furthermore, the 'outsider' status of a non-Ethiopian team member is likely to have affected behaviour of participants. However, this is a recognised challenge when conducting ethnography and was considered when interpreting findings.[30 31] Staff were friendly, cooperative and keen to share their experiences, and often asked observers for advice or feedback.

Interpretation

For peer review only

In our study early detection, care escalation and appropriate management for pre-eclampsia were undermined by a lack of up-to-date guidance based on evidence. Clinical manifestations and conceptualisations of pre-eclampsia are variable. The International Classification of Diseases (ICD-11) has adopted an updated, broader definition of pre-eclampsia to include hypertension and proteinuria or new-onset maternal organ dysfunction, neurological sequelae or fetal growth restriction.[9 32] Inconsistency remains in national and international clinical practice guidelines implementation of the broad definition and the categorisation of ‘severe’ pre-eclampsia or pre-eclampsia with severe features.[33] This is important as guidelines for management and recommendations for interventions are linked to classification of the disorder. These inconsistencies were mirrored in the care we observed in this study.

Early prediction and prophylaxis for pre-eclampsia were not a part of routine care in our study site, nor recommended in Ethiopian guidelines. Most Ethiopian women consume insufficient calcium, but large regional variation exists.[34] Implementation of interventions requiring early contact may be challenging in a context where first trimester care is rare, particularly at levels of the system where providers are competent and confident to prescribe. However, policy makers should consider evidence-based guidance and

For peer review only

international recommendations to support prophylaxis and reduce burden of disease in at risk women.[11 35 36] The updated 2020 national guideline does not address these discrepancies.

Accurate measurement of BP is fundamental to detecting and managing pre-eclampsia. In our study sites both fully automated devices and mercury sphygmomanometry, were used. The latter requires skills and training to ensure accuracy. The haemodynamic adaptations in pregnancy and pre-eclampsia affect the oscillometric measurement used in automated devices. Few automated devices have been validated in pregnancy or pre-eclampsia. The use of unvalidated or uncalibrated automated devices may lead to significant inaccuracies in blood pressure measurement.[37] Accurate and cost-effective devices have been validated in pre-eclampsia and are useful in health systems where low-skilled staff are involved in maternal care.[38-40]

For peer review only

Triage, recognition of risk and care escalation for pre-eclampsia were identified as areas for improvement in our study. In settings where resources are limited, risk prediction models may offer support to staff to target interventions and decision-making around place of care.[41] Our findings concur with previous studies reporting gaps in knowledge and practices

required for early detection and management of pre-eclampsia in facilities in low-resource settings.[42] Health systems constraints, training, supervision and motivation of staff affect clinical performance and outcomes.

Health planners must focus on ensuring that funding in maternity services and the allocation of resources work to address current challenges to equity. This requires understanding contexts of care and how policies are implemented at a local level. Improvements in external factors such as guidelines and infrastructure, must be mirrored by changes in cultures of care, that support a shared understanding of quality, focus on team competencies and supportive leadership. Previous quality improvement (QI) initiatives in Ethiopia have failed due to lack of understanding and adaptation to local contexts.[43] Understanding formal and informal power structures, considering spheres of influence and creating social and reputational incentives for change may improve focus and engagement. A study aiming to measure and improve quality of intrapartum care, showed that having lead hospitals demonstrate the feasibility of their assessment tool helped to promote its wider acceptance, as other hospitals sought to achieve the national recognition given to the lead hospitals.[44] Participatory approaches that bring together women, clinical and managerial staff may help to shift power dynamics where unconstructive hierarchies exist. Developing shared goals,

For peer review only

strategies to achieve them and clarifying roles early on can improve sustainable and effective implementation.

Conclusions

Quality care for pre-eclampsia requires organisational change to create safe spaces for learning and improvement, alongside efforts to put women at the centre of care and ensure providers are equipped with knowledge, resources and support to adhere to evidence-based practice.

National guidelines for HDP including pre-eclampsia should recommend treatment of BP consistently ≥140/90mmHg to reduce the risk of developing severe hypertension and other complications, and to urgently treat severe hypertension ≥160/110mmHg.[9] They should also include guidance for professionals on counselling women about their future risks, including of having a subsequent pregnancy affected by pre-eclampsia, cardiovascular disease and stroke, and strategies to reduce these. As the growing burden of non-communicable disease emerges globally, opportunities should be seized whilst women are engaged with health services to optimise longer-term outcomes.

For peer review only - http://bmjopen.bmj.com/site/about/guidelines.xhtml

More research is required to understand whether evidence established in high resource settings is applicable in LMICs. This should include implementation research and cost-effectiveness of early prediction and prevention strategies.[36]

It is acknowledged that staff are working under difficult conditions within resource constrained systems. This study allows a focus for where to instigate system change to improve the management of pre-eclampsia for women and support staff to deliver quality care. This should include improvements in antenatal screening and detection of hypertension.

Acknowledgements

We gratefully thank all participants for their time and contributions to the study. We wish to thank Sewit Hessebon, Hanna Negussie and Medhanit Getachew.

CH receives support from AMARI as part of the DELTAS Africa Initiative [DEL-15-01. JS is an NIHR Senior Investigator and is supported by the National Institute for Health Research (NIHR) Applied Research Collaboration South London (NIHR ARC South London) at King's College Hospital NHS Foundation Trust.

For peer review only

Competing interests: none

Authorship contributions

TR: Conceptualisation, Methodology, Investigation, Data curation, Formal analysis, Visualisation, Writing - original draft, Writing - reviewing & editing

AHS: Conceptualisation, Funding acquisition, Supervision, Writing - reviewing & editing

JS: Conceptualisation, Funding acquisition, Methodology, Supervision, Writing - reviewing &editing

TEG: Investigation, Formal analysis, Writing - reviewing & editing

RDG: Investigation, Formal analysis, Writing - reviewing & editing

AA: Conceptualisation, Funding acquisition, Writing - reviewing & editing

RM: Conceptualisation, Methodology, Funding acquisition, Supervision, Writing - reviewing& editing

CH: Conceptualisation, Methodology, Funding acquisition, Supervision, Writing - reviewing& editing

Availability of data and materials

For peer review only

The data generated and analysed during this study are not publicly available to protect individual anonymity but may be available from the corresponding author on reasonable request.

Ethical approvals

All methods were carried out in accordance with relevant guidelines and regulations including the Declaration of Helsinki. Ethical approvals were granted by Addis Ababa University (026/18/Psy) and King's College London (LRS-17/18-5526). Institutional level consent was given by hospital management for observational data collection. Interview participants were required to give individual consent.

Funding

This research was funded by the National Institute of Health Research (NIHR) Global Health Research Unit on Health System Strengthening in sub-Saharan Africa, King's College London (GHRU 16/136/54) and CH through an NIHR RIGHT grant (NIHR200842) using UK aid from the UK Government. The views expressed are those of the authors and not necessarily those of the NHS, the NIHR or the Department of Health and Social Care.

For peer review only

References

1. Say L, Chou D, Gemmill A, et al. Global causes of maternal death: a WHO systematic analysis. *Lancet Glob Health* 2014;2(6):e323-33. doi: 10.1016/S2214-109X(14)70227-X [published Online First: 2014/08/12]
2. Abalos E, Cuesta C, Grosso AL, et al. Global and regional estimates of preeclampsia and eclampsia: a systematic review. *Eur J Obstet Gynecol Reprod Biol* 2013;170(1):1-7. doi: 10.1016/j.ejogrb.2013.05.005 [published Online First: 2013/06/12]
3. Abalos E, Cuesta C, Carroli G, et al. Pre-eclampsia, eclampsia and adverse maternal and perinatal outcomes: a secondary analysis of the World Health Organization Multicountry Survey on Maternal and Newborn Health. *BJOG* 2014;121 Suppl 1:14-24. doi: 10.1111/1471-0528.12629 [published Online First: 2014/03/20]
4. Berhan Y, Berhan A. Causes of maternal mortality in Ethiopia: a significant decline in abortion related death. *Ethiop J Health Sci* 2014;24 Suppl:15-28. doi: 10.4314/ejhs.v24i0.3s [published Online First: 2014/12/10]
5. Mersha AG, Abegaz TM, Seid MA. Maternal and perinatal outcomes of hypertensive disorders of pregnancy in Ethiopia: systematic review and meta-analysis. *BMC Pregnancy Childbirth* 2019;19(1):458. doi: 10.1186/s12884-019-2617-8 [published Online First: 2019/12/05]
6. WHO. Global Health Estimates 2019: Deaths by Cause, Age, Sex, by Country and by Region, 2000-2019. https://www.who.int/data/gho/data/themes/mortality-and-global-health-estimates2019 [accessed 20th January 2023.
7. Souza JP, Tuncalp O, Vogel JP, et al. Obstetric transition: the pathway towards ending preventable maternal deaths. *BJOG* 2014;121 Suppl 1:1-4. doi: 10.1111/1471-0528.12735 [published Online First: 2014/03/20]
8. Knight M NM, Tuffnell D, Kenyon S, Shakespeare J, Brocklehurst P, Kurinczuk JJ (Eds.) on behalf of MBRRACE-UK. . Saving Lives, Improving Mothers' Care - Surveillance of maternal deaths in the UK 2012-14 and lesson learned to inform maternity care for the UK and Ireland Confidential Enquiries into Maternal Deaths and Morbidity 2009-14.: Oxford: National Perinatal Epidemiology Unity, University of Oxford 2016, 2016.
9. Brown MA, Magee LA, Kenny LC, et al. Hypertensive Disorders of Pregnancy: ISSHP Classification, Diagnosis, and Management Recommendations for International Practice. *Hypertension* 2018;72(1):24-43. doi: 10.1161/HYPERTENSIONAHA.117.10803 [published Online First: 2018/06/15]
10. NICE. Hypertension in pregnancy: diagnosis and management: National Institute for Health and Care Excellence; 2019 [Available from: https://www.nice.org.uk/guidance/ng133.
11. Rolnik DL, Wright D, Poon LC, et al. Aspirin versus Placebo in Pregnancies at High Risk for Preterm Preeclampsia. *N Engl J Med* 2017;377(7):613-22. doi: 10.1056/NEJMoa1704559 [published Online First: 2017/06/29]
12. Hofmeyr GJ, Lawrie TA, Atallah AN, et al. Calcium supplementation during pregnancy for preventing hypertensive disorders and related problems. *Cochrane Database Syst Rev* 2018;10:CD001059. doi: 10.1002/14651858.CD001059.pub5 [published Online First: 2018/10/03]
13. Magee LA, von Dadelszen P, Rey E, et al. Less-tight versus tight control of hypertension in pregnancy. *N Engl J Med* 2015;372(5):407-17. doi: 10.1056/NEJMoa1404595 [published Online First: 2015/01/30]

For peer review only - http://bmjopen.bmj.com/site/about/guidelines.xhtml

14. Abalos E, Duley L, Steyn DW, et al. Antihypertensive drug therapy for mild to moderate hypertension during pregnancy. *Cochrane Database Syst Rev* 2018;10:CD002252. doi: 10.1002/14651858.CD002252.pub4 [published Online First: 2018/10/03]

15. Which anticonvulsant for women with eclampsia? Evidence from the Collaborative Eclampsia Trial. *Lancet* 1995;345(8963):1455-63. [published Online First: 1995/06/10]

16. Duley L, Gulmezoglu AM, Henderson-Smart DJ, et al. Magnesium sulphate and other anticonvulsants for women with pre-eclampsia. *Cochrane Database Syst Rev* 2010(11):CD000025. doi: 10.1002/14651858.CD000025.pub2 [published Online First: 2010/11/12]

17. Roder-DeWan S, Nimako K, Twum-Danso NAY, et al. Health system redesign for maternal and newborn survival: rethinking care models to close the global equity gap. *BMJ Glob Health* 2020;5(10) doi: 10.1136/bmjgh-2020-002539 [published Online First: 2020/10/16]

18. Hanson C, Waiswa P, Pembe A, et al. Health system redesign for equity in maternal and newborn health must be codesigned, country led, adapted to context and fit for purpose. *BMJ Glob Health* 2020;5(10) doi: 10.1136/bmjgh-2020-003748 [published Online First: 2020/10/16]

19. Kruk ME, Gage AD, Arsenault C, et al. High-quality health systems in the Sustainable Development Goals era: time for a revolution. *Lancet Glob Health* 2018;6(11):e1196-e252. doi: 10.1016/S2214-109X(18)30386-3 [published Online First: 2018/09/10]

20. Vousden N, Lawley E, Seed PT, et al. Incidence of eclampsia and related complications across 10 low- and middle-resource geographical regions: Secondary analysis of a cluster randomised controlled trial. *PLoS Med* 2019;16(3):e1002775. doi: 10.1371/journal.pmed.1002775 [published Online First: 2019/03/30]

21. WHO U, UNFPA, World Bank Group and the United Nations Population Division. Maternal mortality in 2000-2017: Internationally comparable MMR estimates by the Maternal Mortality Estimation Inter-Agency Group (MMEIG); 2019 [Available from: https://www.who.int/gho/maternal_health/countries/eth.pdf.

22. Ethiopia FMoH. Health Sector Transformation Plan II. https://www.familyplanning2020.org/sites/default/files/HSTP-II.pdf, 2021.

23. Vindrola-Padros C, Vindrola-Padros B. Quick and dirty? A systematic review of the use of rapid ethnographies in healthcare organisation and delivery. *BMJ Qual Saf* 2018;27(4):321-30. doi: 10.1136/bmjqs-2017-007226 [published Online First: 2017/12/22]

24. Braun V, Clarke V. Using thematic analysis in pyschology. *Qualitative Research in Pyschology* 2006;3:2, 77-101 doi: 10.1191/1478088706qp063oa

25. Bohren MA, Hunter EC, Munthe-Kaas HM, et al. Facilitators and barriers to facility-based delivery in low- and middle-income countries: a qualitative evidence synthesis. *Reprod Health* 2014;11(1):71. doi: 10.1186/1742-4755-11-71 [published Online First: 2014/09/23]

26. Bohren MA, Mehrtash H, Fawole B, et al. How women are treated during facility-based childbirth in four countries: a cross-sectional study with labour observations and community-based surveys. *Lancet* 2019;394(10210):1750-63. doi: 10.1016/S0140-6736(19)31992-0 [published Online First: 2019/10/13]

27. Bradley S, McCourt C, Rayment J, et al. Disrespectful intrapartum care during facility-based delivery in sub-Saharan Africa: A qualitative systematic review and thematic

For peer review only - http://bmjopen.bmj.com/site/about/guidelines.xhtml

synthesis of women's perceptions and experiences. *Soc Sci Med* 2016;169:157-70. doi: 10.1016/j.socscimed.2016.09.039 [published Online First: 2016/10/23]

28. Burrowes S, Holcombe SJ, Jara D, et al. Midwives' and patients' perspectives on disrespect and abuse during labor and delivery care in Ethiopia: a qualitative study. *BMC Pregnancy Childbirth* 2017;17(1):263. doi: 10.1186/s12884-017-1442-1 [published Online First: 2017/08/24]
29. Knight HE, Self A, Kennedy SH. Why are women dying when they reach hospital on time? A systematic review of the 'third delay'. *PLoS One* 2013;8(5):e63846. doi: 10.1371/journal.pone.0063846 [published Online First: 2013/05/25]
30. Dixon-Woods M. What can ethnography do for quality and safety in health care? *Qual Saf Health Care* 2003;12(5):326-7. doi: 10.1136/qhc.12.5.326 [published Online First: 2003/10/09]
31. Cupit C, Mackintosh N, Armstrong N. Using ethnography to study improving healthcare: reflections on the 'ethnographic' label. *BMJ Qual Saf* 2018;27(4):258-60. doi: 10.1136/bmjqs-2017-007599 [published Online First: 2018/02/22]
32. WHO. International Classification of Diseases (ICD-11) [Available from: https://www.who.int/standards/classifications/classification-of-diseases2019.
33. Scott G, Gillon TE, Pels A, et al. Guidelines-similarities and dissimilarities: a systematic review of international clinical practice guidelines for pregnancy hypertension. *Am J Obstet Gynecol* 2020 doi: 10.1016/j.ajog.2020.08.018 [published Online First: 2020/08/24]
34. Tesfaye B, Sinclair K, Wuehler SE, et al. Applying international guidelines for calcium supplementation to prevent pre-eclampsia: simulation of recommended dosages suggests risk of excess intake in Ethiopia. *Public Health Nutr* 2018:1-11. doi: 10.1017/S1368980018002562 [published Online First: 2018/10/16]
35. WHO recommendation: calcium supplementation during pregnancy for the prevention of pre-eclampsia and its complications. Geneva: World Health Organisation, 2018.
36. Poon LC, Shennan A, Hyett JA, et al. The International Federation of Gynecology and Obstetrics (FIGO) initiative on pre-eclampsia: A pragmatic guide for first-trimester screening and prevention. *Int J Gynaecol Obstet* 2019;145 Suppl 1:1-33. doi: 10.1002/ijgo.12802 [published Online First: 2019/05/22]
37. Gupta M, Shennan AH, Halligan A, et al. Accuracy of oscillometric blood pressure monitoring in pregnancy and pre-eclampsia. *Br J Obstet Gynaecol* 1997;104(3):350-5. doi: 10.1111/j.1471-0528.1997.tb11467.x [published Online First: 1997/03/01]
38. Nathan HL, de Greeff A, Hezelgrave NL, et al. An accurate semiautomated oscillometric blood pressure device for use in pregnancy (including pre-eclampsia) in a low-income and middle-income country population: the Microlife 3AS1-2. *Blood Press Monit* 2015;20(1):52-5. doi: 10.1097/MBP.0000000000000086 [published Online First: 2014/09/23]
39. Nathan HL, Vousden N, Lawley E, et al. Development and evaluation of a novel Vital Signs Alert device for use in pregnancy in low-resource settings. *BMJ Innov* 2018;4(4):192-98. doi: 10.1136/bmjinnov-2017-000235 [published Online First: 2018/10/16]
40. Nathan HL, Boene H, Munguambe K, et al. The CRADLE vital signs alert: qualitative evaluation of a novel device designed for use in pregnancy by healthcare workers in low-resource settings. *Reprod Health* 2018;15(1):5. doi: 10.1186/s12978-017-0450-y [published Online First: 2018/01/07]

For peer review only

41. Payne BA, Hutcheon JA, Ansermino JM, et al. A risk prediction model for the assessment and triage of women with hypertensive disorders of pregnancy in low-resourced settings: the miniPIERS (Pre-eclampsia Integrated Estimate of RiSk) multi-country prospective cohort study. *PLoS Med* 2014;11(1):e1001589. doi: 10.1371/journal.pmed.1001589 [published Online First: 2014/01/28]

42. Ansari N, Manalai P, Maruf F, et al. Quality of care in early detection and management of pre-eclampsia/eclampsia in health facilities in Afghanistan. *BMC Pregnancy Childbirth* 2019;19(1):36. doi: 10.1186/s12884-018-2143-0 [published Online First: 2019/01/20]

43. Aveling EL, Zegeye DT, Silverman M. Obstacles to implementation of an intervention to improve surgical services in an Ethiopian hospital: a qualitative study of an international health partnership project. *BMC Health Serv Res* 2016;16(1):393. doi: 10.1186/s12913-016-1639-4 [published Online First: 2016/08/18]

44. Canavan ME, Brault MA, Tatek D, et al. Maternal and neonatal services in Ethiopia: measuring and improving quality. *Bull World Health Organ* 2017;95(6):473-77. doi: 10.2471/BLT.16.178806 [published Online First: 2017/06/13]

Figure Legend:

Figure 1. Ethiopian Health System Structure

For peer review only

Figure 1. Ethiopian Health System Structure [20]

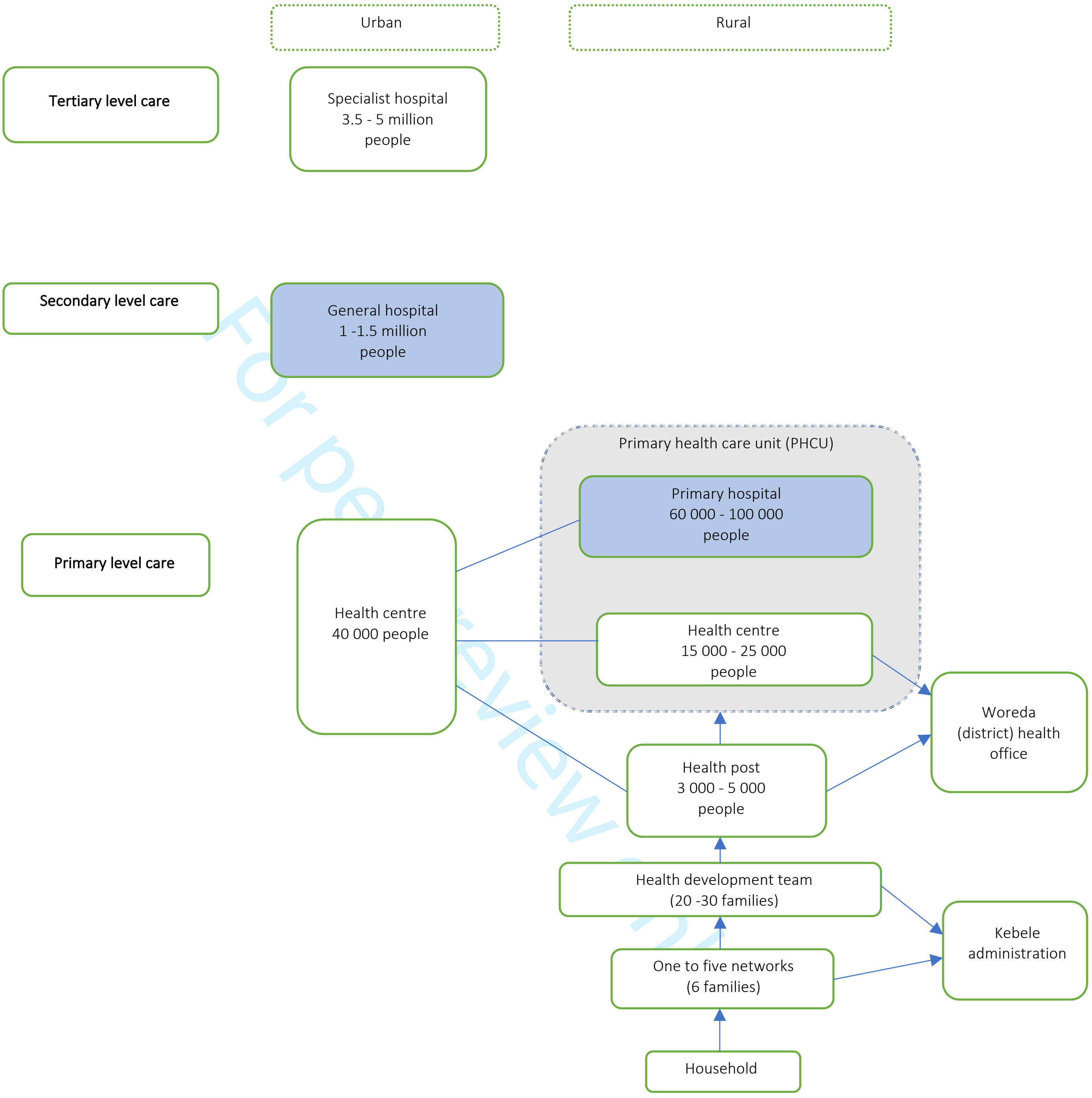

Codebook

Nodes\\Synthesis 1_TR_TE_combined\\Synthesis 2_themes_V1.0_2\\Synthesis 3

Name	Description	Files	References
1.0 Inconsistencies and inequity_Analysis 1	Inconsistency in how the ‘rules' are applied may exacerbate inequities but may also endeavour to ameliorate them	0	0
Clinical factors		0	0
Clinical competence	examples of factors affecting ability to provide safe care. Relates to ability to accurately assess and manage patients. May relate to a lack of awareness, knowledge, training or supervision.	25	48
Clinical uncertainty	Explicit or implicit uncertainty or variance in practice. May relate to decision-making, processes of care, deviance from recommendations in guidelines, complexity in clinical case.	25	43
Recognition of risk or problems	Issues relating to identification of increased risk of complications or need for surveillance/action. Ability to perceive problems.	31	89
Theoretical processes of care_pre-eclampsia	Pathways or processes for how patients with high blood pressure or pre-eclampsia move between levels within or between health facilities, in theory (as opposed to what actually happens/is observed).	11	22
Quality issues		0	0
Adherence to guidelines	Examples of how care relates to national or local guidelines	22	55
Communication between staff	How, when and why staff communicate with each other	23	66
Communication with patients and family	Any communications or verbal interactions with patients or their families, either directly observed or described. The content of, why, when or how, communication with patients and their attendants.	24	89
Conflict between staff	Includes disagreements, differences in opinions or different approaches to problems or situations	10	15

For peer review only - http://bmjopen.bmj.com/site/about/guidelines.xhtml

Name	Description	Files	References
Conscientiousness	Healthcare professionals being diligent and willing to work efficiently	22	41
Consent	Permission to medical treatments, examinations or procedures. May include examples of consent taken/given or not taken/given. May be verbal or written or sometimes implied e.g holding out arm for a blood pressure check.	12	18
Detection or surveillance_pre-eclampsia	Any observations relating to detection of pre-eclampsia including BP monitoring, screening urine or blood tests for end organ damage.	22	54
Quality of documentation	use of written records to note down care given including monitoring (checking vital signs), investigations, treatments, diagnoses, patient details.	27	98
Timely management_pre-eclampsia	Observations or descriptions of process or sequence of care relating to pre-eclampsia, factors relating to reducing or contributing to delays in care for pre-eclampsia or management of high blood pressure.	6	13
Transparency and accountability	Observations or descriptions relating to (un)ethical professional practice, recognition of errors, appropriate action taken. Relates to open communication and good governance. Transparency relates to ensuring information is available to patients and management, to protect patient safety. Accountability to ensure providers/facilities are held responsible for actions or consequences of errors.	19	50
Structural or systems barriers and facilitators		0	0
Access to medications	Issues relating to availability or lack of medications within hospital pharmacy. Relates to organisation with pharmacy, procurement, supply chains and financing.	9	21
Availability and awareness of guidelines	relates to providers/healthcare workers awareness of relevant clinical guidelines and their availability in clinical areas for use	13	26
Availability and utilisation of WASH	Water And Sanitation Hygiene. Availability, access and utilisation of water and sanitation equipment including soap.	14	22
Cleanliness	General cleanliness of rooms and facility. May include factors relating to patient/family behaviour such as disposal of general waste. And/or behaviours or practices of clinical or cleaning staff.	14	30
Electricity supply	Lack of access or supply of electricity	6	13
Inconsistency in directives or care	Failure to standardised procedures. May be due to changes in advice from	16	27

For peer review only - http://bmjopen.bmj.com/site/about/guidelines.xhtml

Name	Description	Files	References
	management, differences in understanding or interpretation of guidance or rules or unclear guidance.		
Organisation of services	Where distinct services are offered and which cadres of staff cover these services.	15	26
Payment for maternal care services	Details of payments required or made by patients or their families for services not provided for free at the point of care. Out of pocket payments.	16	33
Staffing	Details of numbers, cadres of staff providing care, availability out of hours and access to specialists outside daytime hours or on weekends. Includes issues related to understaffing/lack of required staff numbers for safe or effective practice.	23	62
Support and training for staff	References to professional development or learning and support or need for any of these.	16	57
Triage process and initial management	Any observations or descriptions relating to when patient's first arrive in hospital, initial assessment and prioritisation of patients/conditions. Who does this, where, how. Included details of initial place of care, cadres of health work providing care, initial investigations, assessment and any treatments given immediately.	16	48
Workload	Factors influencing the amount of work and productivity of the facility. Related to utilisation of services and workforce structures and numbers.	22	67
2.0 Improvisation_Analysis 1	Making do or tinkering in attempt to mitigate systemic constraints	0	0
Appropriateness of referrals	Factors relating to whether referrals are timely/late AND/OR appropriate or inappropriate in terms of clinical need/level of care at receiving facility AND/OR appropriate communication to receiving hospital in advance	10	16
Availability of clinical investigations	included any lab tests, ultrasound, etc.	12	18
Availability of equipment and materials	Availability of materials and essential equipment e.g gloves, sheets, resuscitation equipment. EXCLUDES medication	28	85
Blood transfusion	Issues relating to availability, access to, planning, supply of blood and blood products including plasma/FFP.	12	29
Clinical decision-making	How, when and where clinicians make decisions about management of patients. e.g whether require admission, involving colleagues/team decision-making	26	53
Decision re mode of delivery	Factors influencing how decisions are made about how to deliver babies e.g	8	12

For peer review only - http://bmjopen.bmj.com/site/about/guidelines.xhtml

Name	Description	Files	References
	Caesarean delivery versus vaginal delivery. Also includes spontaneous vaginal delivery (SVD) versus instrumental birth (ventouse or forceps delivery).		
Decision re timing of delivery	Factors influencing when clinicians may recommend birth, if women present with problems or require intervention.	11	22
Efficiency	How resources, time or people are used to reduce waste. Related to provision of quality care.	13	20
Escalation of care	Processes of referral on to other clinicians or higher level facility, where more skills, knowledge or resources are required in making a clinical assessment or giving treatment.	31	89
Family support	Support from partners or wider family, may be emotional or practical support. Relates to enabling access and provision of appropriate, quality care in terms of patient centred care, timely care if support from family etc.	18	45
Fetal or baby care and monitoring	included observed processes of care or descriptions of how fetal monitoring is carried out including assessment of heart rate or obstetric ultrasound	13	42
Observed processes of care_pre-eclampsia or severe hypertension	Includes observed processes of care for women with severe hypertension (160/110mmHg). Examination, ordering investigations, prescribing medication, admission criteria.	16	36
Observed processes of care_magnesium sulphate	More detailed accounts of magnesium sulphate administration or use including dosing, length of treatment, reasons for initiation or discontinuing.	10	15
Reassessment	rechecking vital signs, clinical condition or investigations. Relates to timely detection of problems, detection of worsening problems, need to escalate and provision of quality care.	18	36
Physical organisation of care	Physical locations within the hospital, designated to particular services and how these are organised.	18	41
Patient flow	the movement of patients through the facility/different stages of care from triage/admission to discharge	5	16
Precarity	Uncertainty or insecurity in care given, may be due to miscommunication or lack of adherence to recommended/evidence-based care.	21	34
Prioritisation	Processes of deciding on relative importance of tasks or problems.	9	19

For peer review only - http://bmjopen.bmj.com/site/about/guidelines.xhtml

Name	Description	Files	References
Privacy	Issues relating to provision of respectful care, including offering to cover patients during examination, providing space for using toilet facilities/bed pan in a space without others around.	18	57
Roles and responsibilities	Descriptions or observations relating to professional roles and responsibilities including particular tasks and duties undertaken.	23	74
Safety	Any issues relating to patient or staff safety including structural issues or processes of care. Includes observations or descriptions of unsafe practice relating to administration of drugs, use of medical devices, unsafe injection practices, hygiene and infection control. Includes appropriate disposal of clinical waste.	20	47
Theoretical processes of care_general	Pathways or processes for how patients move between levels within or between health facilities, in theory (as opposed to what actually happens/is observed). RELATING TO ANY CONDITION	11	17
3.0 Trust and support_Analysis 1		0	0
Acceptance of treatment or advice	Discussion or views about whether patients and/or families accept treatments or management as advised by caregivers. May involve decisional conflict.	20	49
Authority structures	Interactions or examples of hierarchy within the care system. Included both health professionals e.g midwives, nurses, IESO, obstetrician, medical director and auxiliary staff e.g cleaners, lab technicians, and also decision-makers e.g women, partners	16	24
Availability of specialist staff	Access and availability. Includes staff with any specialist skills e.g doctors (obstetrician/gynaecologist), laboratory staff	16	33
Barriers and facilitators to follow up	Challenges that hinder mothers attending for follow up either routine or if notice danger signs/have a problem AND/OR factors that can improve attendance at facility, acceptance of advice or treatments	14	36
Communication with patients and family	Any communications or verbal interactions with patients or their families, either directly observed or described. The content of, why, when or how, communication with patients and their attendants.	24	89
Creating community awareness	Health promotion activities to improve knowledge and awareness of danger signs during pregnancy, birth and postnatally.	14	43
Defensiveness	Behaviour observed or described relating to being anxious or to avoid potential	10	18

For peer review only - http://bmjopen.bmj.com/site/about/guidelines.xhtml

Name	Description	Files	References
	criticism.		
Feedback to referring health centres	System or processes to allow information to go back to referring facilities including clinical outcomes, appropriateness of referrals, appropriateness of initial triage/management, documentation, staff accompanying on referral	7	15
Inertia	Tendency to do nothing/not take action when required despite lack of barriers.	13	25
Maintenance of equipment	includes charging equipment, replacement of batteries or broken parts, servicing equipment etc.	10	18
Neglect	Failure to provide quality care or abandonment of patient needs	13	20
Patient concerns	Any issues relating to anxieties or problems expressed by patients or described by those caring for them. Relates to access and quality of care.	23	60
Professional satisfaction	Health workers feelings about their roles, how they are valued, thoughts about pay/compensation for work and incentives	7	16
Self-appraisal of skills	Assessment by individuals of themselves/their own skills/ability or of the facilities within which they work. Self-reflection.	8	19
Team working	Collaboration between professionals to perform duties including examples of joint decision making (included examples of good or poor team working)	11	26
Trust in resources	Relating to confidence in either physical/material or human resources.	15	18
Undermining	Observations or descriptions of behaviour that diminishes power of an individual to act/carry out their responsibilities.	7	7
Use of traditional medicines	Any issues relating to pregnant women using traditional medicines and how this relates to their care in health facilities.	7	13
Women's experience of care or services	includes their perceptions about quality of care in hospital, continuity of carer, information provided to them, how professional communicated, if they were counselled appropriately (general impressions that may not fit into other codes)	7	25

For peer review only - http://bmjopen.bmj.com/site/about/guidelines.xhtml

Consolidated criteria for reporting qualitative studies (COREQ) checklist

**Number**	**Item**	**Ethiopia**	**Location in manuscript (section, page number)**
**Domain 1: Research team and reflexivity**			
1.	Interviewer/facilitator	RD, TR, TE	Methods, data collection, pg7
2.	Credentials		Methods, data collection, pg7
3.	Occupation	Ethiopian obstetrician, British obstetrician, Social epidemiology research coordinator	Methods, data collection, pg7
4.	Gender	F/F/F	Not included
5.	Experience and training	Prior experience of qualitative data collection	Not included
6.	Relationship established	No	Methods, data collection, pg 8
7.	Participant knowledge of the interviewer	No	Methods, data collection, pg 8
8.	Interviewer characteristics	Involved in wider health system strengthening project	Methods, data collection, pg 7
**Domain 2: study design**			

For peer review only

9.	Methodological orientation and theoretical frameworks	Thematic analysis using an inductive approach	Methods, data analysis, pg 8,9
10.	Sampling	Purposive sampling from routinely collected data	Methods, data collection, pg7
11.	Method of approach	Face to face	Not included
12.	Sample size	21 IDI	Methods, data collection, pg7,8
13.	Non-participations	0	n/a
14.	Setting of data collection	Primary and general hospital	Methods, study setting, pg 6
15.	Presence of non-participants	No	n/a
16.	Description of sample	Yes	Methods, data collection, pg7,8
17.	Topic guide	Example guide included as supplementary file.	n/a
18.	Repeat interviews	No, further sampling where data saturation needed e.g women with previous pre-eclampsia	Not included

For peer review only

19.	Audio recorded	Yes	Methods, data collection, pg 8
20.	Field notes	Yes	Methods, data collection, pg 7
21.	Duration of interviews/FGD	Variable	Not included
22.	Data saturation	Yes	Not included
23.	Transcripts returned for participant checking	No, but member checking via Theory of Change workshops for later stage of wider projects	Methods, data collection, pg 8
**Domain 3: analysis and findings**			
24.	Number of data coders	2	Methods, data analysis, pg9
25.	Description of coding tree	Yes - provided in supplementary file	Not included in main manuscript.
26.	Derivation of themes	Derived from data iteratively	Methods, data analysis, pg 8,9
27.	Software	NVivo 12	Methods, data analysis, pg 8
28.	Participant checking	Yes. See 23.	Methods, data collection, pg 8

For peer review only

29.	Quotations presented	Yes, participant ID provided	Results, pg 9 - 19
30.	Data findings consistent	Yes	Strengths and Limitations, pg4
31.	Clarity of major themes	Yes, quotations to support throughout results section and in Table 3	Results, pg 9 - 19
32.	Clarity of minor themes	Yes, see coding structure in supplementary file	Not included in main manuscript.

For peer review only
